# Research Progress on Biomaterials with Immunomodulatory Effects in Bone Regeneration

**DOI:** 10.1002/advs.202501209

**Published:** 2025-08-13

**Authors:** Jianan Li, Ying Qu, Bingyang Chu, Tingkui Wu, Meng Pan, Dong Mo, Lang Li, Yang Ming, Yun Yang, Meng Wang, Xinlong He, Zhiyong Qian

**Affiliations:** ^1^ Department of Biotherapy Cancer Center and State Key Laboratory of Biotherapy West China Hospital Sichuan University Chengdu 610041 China; ^2^ Department of Hematology and Institute of Hematology West China Hospital Sichuan University Chengdu 610041 China; ^3^ Department of Pediatric Surgery West China Hospital Sichuan University Chengdu 610041 China; ^4^ Department of Orthopedics West China Hospital Sichuan University Chengdu 610041 China; ^5^ Departments of Diagnostic Radiology Yong Loo Lin School of Medicine National University of Singapore Singapore 119074 Singapore

**Keywords:** bioactive molecule delivery, biomaterial properties, bone regeneration, immune modulation, macrophages

## Abstract

The immune system plays a pivotal role in bone regeneration, and biomaterials engineered to modulate immune responses present a promising strategy for the treatment of extensive bone defects and osteolytic conditions. This review critically evaluates recent advancements in immunomodulatory biomaterials for bone repair, integrating perspectives from both immunology and materials science. It offers a comprehensive analysis of key design strategies and the underlying principles guiding the development of these biomaterials, with a particular focus on their physical and chemical properties, bioactive molecule delivery systems, cell transplantation techniques, and responses to external stimuli. Additionally, this review examines the interactions between these biomaterials and immune cells, particularly macrophages, within various pathological contexts. Finally, the paper discusses current challenges and future directions, aiming to provide researchers and clinicians with a valuable resource for advancing this rapidly evolving field.

## Graphical abstract

This review highlights recent advanced in immunomodulatory biomaterials for bone repair, focusing on design strategies, immune interactions, and clinical potential.

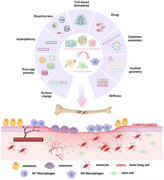

## 1 Introduction

The human skeleton is a multifunctional organ system that provides structural support, protection, facilitation of movement, hematopoiesis, and mineral storage. While these materials possess intrinsic capacities for self‐repair and remodeling, they are generally limited to defects below a critical size. Under complex pathological conditions,^[^ ^1^ ^]^ such as extensive bone defects, certain metabolic disorders, inflammation, or bone loss associated with autoimmune diseases, the natural regenerative capacity of bone is often insufficient.^[^ ^2^ ^]^ This limitation highlights the pressing need for advancements in bone regeneration strategies. In this context, bone tissue engineering has evolved as a crucial research frontier, aiming to develop advanced methodologies for effective bone repair and reconstruction.^[^ ^3^, ^4^ ^]^

Bone reconstruction involves a sophisticated biological program that requires coordinated interplay between multiple cellular populations and molecular signaling cascades.^[^ ^5^ ^]^ Central to this process is the immune system, which not only functions as a defense mechanism by identifying and eliminating foreign entities such as pathogens, necrotic tissues, or xenogeneic cells but also plays an active role in regulating bone remodeling.^[^ ^6^ ^]^ In addition to its defensive role, the immune system significantly influences bone remodeling outcomes by mediating inflammatory responses,^[^ ^7^ ^]^ promoting angiogenesis, and regulating the activities of osteoblasts and osteoclasts through diverse mechanisms and signaling pathways.^[^ ^8^, ^9^ ^]^ Notably, modulation of the immune microenvironment has emerged as a critical factor shaping the success of bone regeneration. This understanding offers novel therapeutic opportunities for addressing bone‐related disorders and enhancing regenerative strategies.^[^ ^10^ ^]^

Current research on bone tissue engineering emphasizes the stimulation of osteogenic differentiation and vascularization in stem cells.^[^ ^11^, ^12^ ^]^ Increasing evidence suggests that immune modulation plays a pivotal role in bone regeneration.^[^ ^13^ ^]^ In this context, biomaterials are predominantly utilized as carriers or scaffolds, providing essential physical support, chemical cues, and biological signals that increase both the rate and quality of bone regeneration.^[^ ^14^, ^15^, ^16^ ^]^ Upon implantation, biomaterials are recognized by the host as foreign bodies, triggering an immune response.^[^ ^17^ ^]^ These findings underscore the critical importance of integrating the immune system's role into the design and application of biomaterials for bone regeneration. Understanding how these materials influence the immune microenvironment to facilitate bone repair and reconstruction remains a crucial focus of ongoing investigations.^[^ ^18^, ^19^, ^20^ ^]^

This review examines the immunomodulatory strategies essential forbone healing, focusing on how biomaterials can facilitate this process by modulating immune functions from various perspectives and at multiple levels. First, we provide an overview of the bone regeneration process, emphasizing the role and mechanisms of the immune system in this context. Next, we summarize the primary approaches through which biomaterials contribute to immune modulation, including their physical properties, chemical characteristics, bioactive molecule delivery systems, cell transplantation techniques, and responsiveness to external stimuli. We subsequently discuss the regulation of the immune microenvironment by biomaterials under diverse pathological conditions, highlighting their mechanisms and effects on bone regeneration. Finally, we address the current challenges in the field of biomaterial‐driven immune modulation for bone regeneration and explore future directions. This review aims to offer valuable insights to advance the application of biomaterials in bone repair and regeneration.

## 2 Bone Regeneration and Immunity

Bone is a vital organ within the human body,^[^ ^21^ ^]^ serving as a structural framework that protects internal organs and facilitates essential bodily functions.^[^ ^22^ ^]^ It plays a crucial role in metabolic regulation, independent mobility, hematopoiesis, and immune system modulation.^[^ ^23^, ^24^ ^]^ Fractures, the most common form of major organ trauma, not only diminish quality of life but also impose a significant economic burden on healthcare systems.

Bone possesses a natural ability to self‐heal in small‐sized injuries under healthy conditions. However, this regenerative capacity is markedly diminished in complex scenarios, such as infections or when defects exceed critical dimensions, often leading to incomplete regeneration and nonuniform bone formation. Furthermore, aging‐related factors further compromise the regenerative potential of bone. Conditions such as chronic inflammation, obesity, metabolic diseases, and immunodeficiency disorders significantly impair natural repair processes, posing substantial challenges to effective bone healing.

Bone grafting is widely regarded as the clinical “gold standard” for fracture treatment. However, its effectiveness is constrained by several factors, including donor site morbidity, immune rejection, and infection risk. In response, bone tissue engineering has emerged as a promising alternative therapeutic strategy.^[^ ^25^ ^]^ Recent advancements in bone repair biomaterials have focused primarily on enhancing osteoblast differentiation and promoting angiogenesis. However, the presence of immune cells and a well‐regulated immune microenvironment are equally critical for effective bone tissue repair. Therefore, the ability to modulate and manipulate immune processes via biomaterials is essential for achieving successful bone regeneration and facilitating their translation into clinical applications.

### 2.1 Bone Immunomodulatory Microenvironment

The bone immunomodulatory microenvironment is a highly coordinated network in which immune cells, skeletal cells, and molecular mediators dynamically interact to regulate bone homeostasis, repair, and remodeling. This microenvironment is characterized by temporal and spatial crosstalk between inflammatory and regenerative signals, which affects the outcome of bone healing. This dysregulation of the microenvironment underlies a variety of bone pathologies, making it a key target for biomaterial‐based intervention.^[^ ^26^, ^27^ ^]^ Bone immunomodulation involves intricate interactions among diverse cell types, cytokines, and their regulatory mechanisms.^[^ ^28^ ^]^ Cellular components within this niche include resident and recruited immune cells, with **macrophages** serving as central orchestrators that dynamically balance inflammation and regeneration through phenotypic polarization. **M1 macrophages**, which are activated by pathogens or tissue damage‐associated molecular patterns (DAMPs), secrete proinflammatory cytokines to eliminate debris but simultaneously exacerbate bone resorption by upregulating receptor activator of nuclear factor kappa‐Β ligand (RANKL) expression. In contrast, **M2 macrophages** resolve inflammation and promote osteogenesis by releasing anti‐inflammatory mediators and osteoinductive factors, while enhancing angiogenesis to support bone repair. **Tlymphocytes** regulate bone metabolism through subset‐specific mechanisms: **Th17 cells** drive osteoclastogenesis via IL‐17‐mediated RANKL upregulation, whereas **regulatory T cells (Tregs)** suppress inflammatory cascades through interleukin‐10 (IL‐10) secretion and direct cellular interactions with mesenchymal stem cells (MSCs) to increase osteoblast activity. **B lymphocytes** exert dual roles by producing osteoprotegerin (OPG), a decoy receptor that inhibits RANKL‐induced osteoclast differentiation, and autoantibodies that may paradoxically exacerbate bone loss in autoimmune disorders.^[^ ^29^ ^]^ Although **neutrophils** are essential for early‐stage pathogen clearance, their overproduction of reactive oxygen species (ROS) in chronic conditions such as diabetic bone defects perpetuates oxidative stress, impairing vascularization and delaying healing. The cytokine landscape further dictates bone remodeling outcomes. Proinflammatory cytokines dominate in pathologies such as rheumatoid arthritis and osteoporosis, driving excessive bone resorption through nuclear factor kappa‐light‐chain‐enhancer of activated B cells/mitogen‐activated protein kinase (NF‐κB/MAPK) pathway activation and Wnt/β‐catenin signaling suppression.^[^ ^10^ ^]^ Conversely, anti‐inflammatory mediators promote tissue repair by polarizing macrophages toward the M2 phenotype, stimulating MSC osteogenic differentiation, and stabilizing nascent vascular networks. This delicate equilibrium underscores the therapeutic potential of targeting immuno‐microenvironmental crosstalk to restore skeletal homeostasis. Pathological perturbations, such as chronic inflammation in osteoporosis or infection in osteomyelitis, disrupt this equilibrium. For example, sustained tumor necrosis factor‐α (TNF‐α) signaling in rheumatoid arthritis accelerates RANKL‐driven osteoclastogenesis while inhibiting osteoblast maturation.^[^ ^30^ ^]^

In summary, the bone immunomodulatory microenvironment represents a therapeutic frontier where understanding cellular crosstalk and cytokine networks enables the design of advanced biomaterials to restore skeletal integrity in patients with degenerative and inflammatory bone diseases.

### 2.2 Bone Regeneration Process

Bone regeneration is a complex and highly coordinated process involving a diverse array of cells, molecules, and signaling pathways.^[^ ^31^ ^]^ This review explores the roles and mechanisms of immune cells, cytokines, and signaling pathways across the various stages of bone regeneration, offering a comprehensive overview of the interconnected processes that underpin this vital physiological function.

The initial stage of bone regeneration is characterized by the formation of fibrin thrombi and the regulation of the immune response. Upon bone injury, platelets release coagulation factors, triggering the coagulation cascade. This cascade results in the conversion of soluble fibrinogen into insoluble fibrin by thrombin, culminating in the formation of fibrin clots. These clots not only play a critical role in hemostasis but also serve as a structural framework for the subsequent inflammatory phase and the initiation of bone repair.^[^ ^32^ ^]^

Following hemostasis, the acute inflammatory phase begins and isdriven by the activity of various immune cells. Within the first 24 h after bone injury, damaged cellular debris releases proinflammatory signals known as DAMPs, while bacterial or viral invasion of the injured tissue triggers the release of pathogen‐associated molecular patterns (PAMPs). These molecular patterns play pivotal roles in recruiting immune cells by activating pattern recognition receptors, such as Toll‐like receptors, on immune cells. This activation stimulates the secretion of inflammatory mediators, including cytokines and chemotactic factors, which amplify the immune response. The released cytokines facilitate the recruitment, migration, and infiltration of immune cells, aid in the clearance of necrotic tissue and foreign matter, and regulate the activity of stem cells essential for bone regeneration.^[^ ^33^ ^]^

During the acute inflammatory phase, immune cells play dual roles: clearing damaged tissue and pathogens while promoting repair by releasing growth factors and other signaling molecules. Among these, macrophages demonstrate remarkable phenotypic plasticity, adapting to environmental cues by differentiating into either proinflammatory M1 or reparative M2 phenotypes. M1 macrophages release proinflammatory cytokines, such as TNF‐α and interleukin‐1β (IL‐1β), which are critical for pathogen elimination and debris clearance. Conversely, M2 macrophages secrete anti‐inflammatory and tissue‐repair molecules, including IL‐10 and transforming growth factor‐β (TGF‐β), which are essential for new tissue formation and the healing process.^[^ ^34^ ^]^

During the initial stages of bone regeneration, immune cells in the wound area, such as macrophages, neutrophils and dendritic cells, release a variety of inflammatory and growth factors, including interleukin‐1 (IL‐1), interleukin‐6 (IL‐6), and TNF‐α. TNF‐α has been reported to negatively regulate bone homeostasis in some inflammatory diseases, inhibiting osteoblast differentiation by inhibiting the Wnt signaling pathway. These factors not only mediate the inflammatory response but also play a direct role in promoting angiogenesis. The formation of new blood vessels is essential for delivering nutrients and oxygen to the damaged area, supporting the growth and maturation of new bone tissue.^[^ ^35^ ^]^ Among the key growth factors involved, vascular endothelial growth factor (VEGF) is particularly critical for angiogenesis. VEGF, which is produced predominantly by macrophages and other immune cells, stimulates endothelial cell proliferation and differentiation while increasing vascular permeability, facilitating the formation of new vascular networks. Concurrently, macrophages contribute to wound clearance by phagocytosing dead cells and tissue debris, creating space for vascular and tissue regeneration. The next stage in bone healing is the proliferative phase, during which the hematoma fibers formed earlier are gradually replaced by fibrous and connective tissue.^[^ ^36^ ^]^ Concurrently, bone marrow stromal cells (BMSCs) are recruited from surrounding tissues to the site of bone injury, where they differentiate into osteoblasts and chondrocytes, contributing to cartilage scar formation and subsequent calcification. Inflammatory factors secreted by immune cells such as IL‐1, IL‐6, and TNF‐α, etc. along with growth factors, such as VEGF and platelet‐derived growth factor (PDGF), play critical roles in stimulating the proliferation and migration of BMSCs. The activationof these bioactive factors is integral to both innate and adaptive immune responses and mobilizes the activity of various immune cells, including polymorphonuclear neutrophils (PMNs), dendritic cells (DCs), macrophages, T cells, and B cells.^[^ ^37^ ^]^

Simultaneously, BMSCs exert regulatory effects on osteoblasts and osteoclasts through paracrine signaling and exosomal interactions, thereby modulating bone healing processes. The final stage of bone healing involves calcification of the bone scab and the initiation of bone remodeling. Bone remodeling is a cyclical process comprising three successive stages: osteolysis, bone reversal, and osteogenesis. This process is orchestrated through dynamic interactions and mutual regulation among osteocytes, osteoclasts, and osteoblasts. Moreover, studies have shown that osteogenesis and osteolysis are influenced by immune cells through shared regulatory pathways, such as the receptor activator of nuclear factor κB–receptor activator of nuclear factor κB ligand–osteoprotegerin (RANKL‐RANK‐OPG) axis and the sphingosine‐1‐phosphate–sphingosine‐1‐phosphate receptor 1 (S1P‐S1PR1) axis, a phenomenon referred to as bone immunomodulation.^[^ ^38^, ^39^ ^]^

In summary, immune regulation plays a pivotal role in bone healing, influencing not only the modulation of inflammatory processes during the early stages but also bone formation and resorption.^[^ ^40^ ^]^ These latter processes are further subject to feedback regulation by the bone tissue itself. A comprehensive understanding of the mechanisms underlying immunomodulation during bone healing is essential for developing effective strategies to repair bone damage.

### 2.3 Inflammatory Response

The inflammatory response constitutes the initial phase of bone healing following injury and is essential for initiating repair processes. Upon bone injury, the rupture of blood vessels results in substantial bleeding and the formation of a hematoma composed of red blood cells, white blood cells, and fibrin. This hematoma not only initiates a cascade of immune responses but also acts as a temporary biological scaffold at the injury site.^[^ ^41^ ^]^ The hypoxic environment, elevated lactic acid levels, reduced pH, and potential microbial invasion at the defect site collectively stimulate the migration and infiltration of immune cells, marking the beginning of the proinflammatory phase of bone healing.^[^ ^42^ ^]^

Numerous immune cells canadapt to harsh microenvironments, such as hypoxia.^[^ ^43^ ^]^ Neutrophils, the primary responders of innate immunity, play a critical role by secreting proteases and antibacterial agents to eliminate necrotic cells and inhibit pathogen proliferation at the injury site.^[^ ^44^ ^]^ Moreover, neutrophils promote the recruitment and polarization of macrophages toward the proinflammatory M1 phenotype through the release of inflammatory mediators, including TNF‐α, IL‐1, and IL‐6.^[^ ^45^ ^]^ They also facilitate the infiltration of other immune cells by secreting chemokines such as C‐X‐C motif chemokine ligand 1 (CXCL1) and C‐X‐C motif chemokine ligand 2 (CXCL2).^[^ ^46^ ^]^

Macrophages, known for their remarkable plasticity, are critical components of innate immunity.^[^ ^47^, ^48^, ^49^ ^]^ During the early inflammatory phase, most macrophages exhibit a proinflammatory M1 phenotype.^[^ ^50^ ^]^ These M1 macrophages are induced by proinflammatory signals such as TNF‐α, interferon‐γ (IFN‐γ), IL‐1,^[^ ^51^ ^]^ inducible nitric oxide synthase (iNOS) and interleukin‐12 (IL‐12). M1 macrophages are highly efficient in antigen presentation and play a key role in initiating acute inflammatory responses and clearing necrotic cells or bacteria at the injury site.^[^ ^52^, ^53^ ^]^ However, a prolonged inflammatory microenvironment characterized by excessive cytokine production can impair bone healing and may lead to fibrous scar formation around biomaterial implants, highlighting the need to downregulate M1 macrophages at appropriate stages.^[^ ^54^ ^]^ In contrast, the transition from the proinflammatory M1 phenotype to the anti‐inflammatory M2 phenotype is pivotal for osteogenic differentiation and successful bone regeneration.^[^ ^55^ ^]^ M2 macrophages are essential for suppressing inflammation, promoting tissue repair, and regulating immune responses. They can be induced by Th2 cytokines, such as interleukin‐4 (IL‐4) and interleukin‐13 (IL‐13), as well as by immune complexes, IL‐10, and glucocorticoids. This phenotypic shift underscores the dynamic role of macrophages in balancing inflammation and repair during bone healing.^[^ ^56^, ^57^ ^]^

Moreover, macrophage phenotypes influence the direction of MSCs differentiation. Macrophages can affect the fate of BMSCs by secreting extracellular vesicles (EVs), such as exosomes (Exos). Studies have shown that macrophage‐derived extracellular vesicles regulate the expression of osteogenic differentiation genes in BMSCs by transferring microRNAs, including miR‐233 and miR‐146a. This process shapes the differentiation trajectory of BMSCs, thereby influencing bone healing.^[^ ^58^ ^]^ The transition from M1 to M2 macrophages plays a critical role throughout the bone reconstruction process, with balanced polarization of M1/M2 macrophages being essential for effective bone tissue repair.^[^ ^59^, ^60^, ^61^, ^62^ ^]^ Consequently, precise spatiotemporal regulation of macrophage activation represents a promising therapeutic strategy for enhancing bone regeneration and repair.^[^ ^63^, ^64^, ^65^ ^]^

T cells and B cells, which are characterized by high specificity and diversity, migrate to the site of bone defects via the lymphatic system and play pivotal roles in adaptive immunity. These cells secrete cytokines and chemokines that modulate the intensity and duration of the inflammatory response. For instance, T cells emit proinflammatory factors to mitigate excessive inflammation.^[^ ^66^ ^]^ Additionally, both T and B cells secrete growth factors such as VEGF and bone morphogenetic proteins (BMPs), which promote vascularization and bone formation, thus contributing to the subsequent stages of bone healing. Notably, subpopulations of these cells, including Tregs, act as immunosuppressive cells that can effectively suppress the inflammatory response during tissue injury, reduce the level of proinflammatory cytokines in local tissues, and are thought to have an immunoregulatory function, which is beneficial to the bone healing process. Th17 cells are also a subgroup of T cells that produce cytokines such as IL‐17 to induce the expression of RANKL in osteoclasts to promote bone resorption. In addition, B cells differentiate and mature in the bone marrow cavity and secrete OPG to inhibit osteoclast differentiation and promote bone regeneration.

### 2.4 Angiogenesis

Angiogenesis is a crucial component of bone regeneration during the repair process, and its regulation is closely linked to immune activities.^[^ ^53^, ^67^ ^]^ he interactions between angiogenesis and immune regulation are reciprocal, with each process influencing the other.^[^ ^68^, ^69^ ^]^ During the inflammatory phase, angiogenesis not only promotes the activation and recruitment of immune cells but also, in turn, is regulated by immune cells through the secretion of cytokines and chemokines.^[^ ^70^, ^71^ ^]^ Notably, macrophage polarization has a significant effect on angiogenic activity. Thrombospondin‐1 (TSP‐1) secreted by M1 macrophages^[^ ^72^ ^]^ and VEGF secreted by M2 macrophages regulate endothelial cell proliferation and migration.^[^ ^73^ ^]^ Thus, macrophages play a central role in the angiogenic switch during bone repair. In the proliferative phase, immune cells influence angiogenesis through their polarization states,^[^ ^43^ ^]^ whereas cytokines released by these cells regulate both the pace and quality of angiogenesis during bone healing.^[^ ^74^ ^]^

Various immune cells and their secreted cytokines, such as TNF‐α, IL‐1, IL‐6, and interleukin‐8 (IL‐8), play crucial roles in modulating VEGF expression, thereby either promoting or inhibiting angiogenesis. VEGF interacts with its receptor (VEGFR), initiating downstream signaling pathways, such as phosphatidylinositol 3 kinase/Akt (PI3K/Akt),^[^ ^75^ ^]^ and recombinant phospholipase C Gamma (PLCγ), etc. This signaling cascade stimulates endothelial cell proliferation and migration, as well as the recruitment and differentiation of surrounding cells. Additionally, endothelial cells themselves release cytokines and chemical mediators that regulate vascular dynamics. For example, nitric oxide (NO) and prostacyclin (PGI2) are critical in controlling vascular relaxation, which enhances blood flow and facilitates the delivery and infiltration of immune cells. In contrast, endothelin‐1 (ET‐1) acts as a key regulator of vasoconstriction. Furthermore, different types of blood vessels express specific molecular markers, such as platelet endothelial cell adhesion molecule (CD31) and alkaline phosphatase, which further influence immune system activity and balance.^[^ ^76^ ^]^

### 2.5 Regulation of Osteoblasts and Osteoclasts

Osteoblasts and osteoclasts are essential for maintaining the balance between bone formation and resorption and play a pivotal role in bone regeneration.^[^ ^77^, ^78^ ^]^ The interplay between the skeletal system and the immune system is also crucial for bone reconstruction.^[^ ^79^ ^]^ BMSCs can be stimulated by various cytokines to differentiate into osteoblasts, which secrete bone matrix to promote tissue mineralization and bone formation.^[^ ^80^, ^81^ ^]^ Osteoclasts, which are multinucleated cells derived from macrophages, attach to bone surfaces and secrete proteases to resorb and degrade bone tissue.^[^ ^82^ ^]^ The balance between osteoblasts and osteoclasts plays an important role in bone remodeling, which is also regulated by the immune system.^[^ ^83^ ^]^ Immune cells, including neutrophils, macrophages, T cells, and B cells, secrete a variety of cytokines and chemical mediators that influence the activity and interactions between osteoblasts and osteoclasts, thereby regulating the equilibrium of bone formation and resorption.

Cytokines secreted by macrophages regulate both inflammation and the differentiation of MSCs, as well as osteoblast function.^[^ ^84^ ^]^ M1 macrophages secrete proinflammatory cytokines such as IL‐1β, TNF‐α, and IL‐10, which inhibit osteoblast alkaline phosphatase activity and the expression of bone‐related proteins, such as type I collagen. These cytokines also suppress the secretion of extracellular bone matrix and the formation of calcified nodules. Additionally, M1 macrophages downregulate the expression of nuclear factor of activated T cell cytoplasmic 1 (NFATc1), a key factor required for osteoclast differentiation, thereby inhibiting bone resorption. In contrast, M2 macrophages promote osteogenic differentiation of MSCs. BMP, a critical factor in osteogenic differentiation, and TGF‐β, which is upstream of the BMP signaling pathway, both have stimulatory effects on osteoblast differentiation.^[^ ^33^ ^]^

T cells and B cells play critical roles in regulating the balance between osteoblast and osteoclast activity. Cytokines such as IL‐4 and IL‐10 secreted by Th2 cells and Bregs enhance the expression of bone proteins and bone mineralization, thereby accelerating bone healing. In contrast, cytokines such as interleukin‐17 (IL‐17), produced by Th17 cells, trigger inflammatory responses, stimulate bone resorption, and impair bone repair. Additionally, TGF‐β and other factors released by Tregs attenuate inflammation, activate the BMP signaling pathway, and ultimately promote osteogenesis, supporting the bone healing process.

Several signaling pathways regulate the balance between osteoblast and osteoclast activity. Key cytokines, including TNF‐α, IL‐6, IL‐10, and IL‐1β, modulate the expression of RANKL, which binds to receptors on osteoclast precursors. RANKL activation triggers the NF‐κB and c‐Fos signaling pathways, promoting osteoclast differentiation and bone resorption. OPG, which acts as a decoy receptor, blocks the interaction between RANKL and its receptor (RANK), thereby inhibiting osteoclast‐mediated bone resorption. Moreover, the expression of OPG is regulated by various immune system‐derived cytokines, further influencing bone remodeling.

The BMP signaling pathway, which regulates both osteoblast and osteoclast function, is modulated by a variety of immune system‐derived cytokines and chemical mediators, including TNF‐α, IL‐1β, IL‐6, IL‐10, IL‐17, and interleukin‐23 (IL‐23). Specifically, BMP signaling promotes osteoblast differentiation and bone formation by binding to BMP receptors, which activate both the Smad and non‐Smad pathways. BMP signaling also inhibits the Wnt pathway,^[^ ^85^ ^]^ reducing osteoclast differentiation and bone resorption. Additionally, interleukin‐15 (IL‐15), produced by T cells and peripheral blood monocytes, promotes osteoclast differentiation and bone resorption by upregulating RANKL and phospholipase D‐1 (PLD1) expression. This effect is mediated through the activation of the MAPK and NF‐κB signaling pathways, thereby promoting osteoclast formation and enhancing bone destruction.^[^ ^86^ ^]^ In addition, the NF‐κB pathway is central to M1 polarization. Upon stimulation by DAMPs or PAMPs, IκB kinase (IKK) phosphorylates IκBα, leading to its degradation and nuclear translocation of NF‐κB. This activates transcription of proinflammatory cytokines (e.g., TNF‐α, IL‐6) and iNOS, exacerbating osteoclastogenesis via RANKL upregulation. In contrast, IL‐4/IL‐13 signaling through the PI3K/Akt axis phosphorylates and inactivates Forkhead Box O1(FoxO1), promoting M2 marker expression. These pathways are therapeutic targets for biomaterials aiming to resolve inflammation and enhance osteogenesis.^[^ ^87^ ^]^ ROS play multifaceted roles in bone regeneration, primarily through their involvement in immune modulation. Rather than merely being by‐products of cellular metabolism, ROS function as signaling molecules that regulate immune responses and promote tissue regeneration. During the inflammatory phase of bone repair, ROS modulates the intensity and duration of inflammation by influencing macrophage polarization. In addition, ROS contributes to bone tissue formation and remodeling by affecting the differentiation and activity of osteoblasts. ROS enhances osteoblast maturation and mineralization, but excessive ROS levels may increase osteoclast activity, accelerating bone degradation. As intracellular signaling molecules, ROS also regulate cell growth, differentiation, and survival by modulating key signaling pathways such as the MAPK and NF‐κB pathways.^[^ ^88^ ^]^ These pathways play a crucial role in bone cell regulation, with ROS promoting osteoclast formation and activation through the RANKL signaling pathway.

In summary, the interactions among immune cells, cytokines, and their associated signaling pathways are central to the process of bone regeneration.^[^ ^89^ ^]^

## 3 Biomaterials for Immune Modulation in Bone Regeneration

Bone tissue engineering is a technique that utilizes biomaterials, bioactive factors, or cells to address bone defects.^[^ ^90^ ^]^ An ideal biomaterial for bone tissue engineering should demonstrate excellent biocompatibility, meaning it integrates effectively with natural bone and promotes synergistic interactions with surrounding bone tissue.^[^ ^91^, ^92^ ^]^ Additionally, the implantation of biomaterials into the body triggers an immune response. If the immune reaction is excessively hostile, it can lead to severe rejection of the implant,^[^ ^93^ ^]^ resulting in encapsulation by fibrous tissue and subsequent implant fibrosis, which impedes bone healing.^[^ ^94^, ^95^ ^]^ Conversely, when biomaterials stimulate an appropriate immune response at the site of a bone defect, they can enhance the regenerative process, supporting successful bone repair and regeneration.^[^ ^96^, ^97^, ^98^ ^]^

The immunomodulatory role of biomaterials in bone healing is influenced by their inherent physicochemical properties, including shape, size, porosity, surface roughness, surface charge, and degradability. Additionally, the ability of biomaterials to carry and release cells and bioactive molecules—such as cytokines, peptides, and drugs—further contributes to their capacity to modulate the immune microenvironment and promote osteogenesis. Given the pivotal role of immune regulation in bone healing,^[^ ^99^, ^100^ ^]^ biomaterials can be strategically engineered to influence the immune response at the injury site. This approach provides a novel perspective for bone tissue engineering and offers significant theoretical and clinical implications, paving the way for more effective treatments for bone repair and regeneration.^[^ ^18^, ^101^, ^102^ ^]^

### 3.1 Immune Modulation by the Physical Properties of Biomaterials

Biomaterials implanted at bone defect sites exhibit a range of characteristics that can effectively activate and modulate the immune response.^[^ ^103^ ^]^ Ideally, the physical properties of these materials should closely resemble those of natural bone, promoting integration and facilitating bone healing.^[^ ^104^, ^105^ ^]^ Key factors such as surface roughness, morphology, scaffold geometry, pore size, porosity, and surface charge influence how biomaterials interact with blood and surrounding tissues, thereby shaping the immune microenvironment at the injury site.^[^ ^106^ ^]^ For example, titanium surfaces with varying degrees of roughness can direct macrophage polarization toward the M2 phenotype, enhancing osseointegration. In contrast, titanium surfaces that are either excessively smooth or rough may hinder osseointegration.^[^ ^107^, ^108^, ^109^, ^110^, ^111^ ^]^ Moreover, the shape and size of biomaterials can affect macrophage phagocytosis and polarization, whereas porosity governs the infiltration and activation of immune cells. The surface charge of biomaterials also plays a critical role in modulating antigen adsorption and presentation, further influencing immune responses.^[^ ^112^, ^113^, ^114^ ^]^ Here, the physical properties of the biomaterials are summarized and classified according to the types of applications in **Table** **1**.

**Table 1:** Immune modulation by physical properties of biomaterials.

Engineering parameters	Materials	Property	Outcome	Refs.
Surface roughness and topography	AuNP	The surface chemistry (amine or acrylic acid) and scale of the nanotopography (16, 38, and 68 nm)	68ACpp inhibited inflammation, polarizing toward M2 macrophages	[118]
	Ti	SLA and TNT	The Ti implants treated with TNT surface treatment show the ability to inhibit inflammation and antioxidant	[119]
	Gear‐inspired bioceramic scaffolds	The bionic gear ceramic scaffolds with different groove widths (G10, G20, G50 and G100) or depths (D30, D70 and D100)	The M2 phenotype polarization of G20 induced macrophages is the best effect	[120]
	PLLA	PLLA stents (aligned microfibers, aligned nanofibers, random microfibers, and random nanofibers)	The random nanofibrous PLLA scaffold reduces the secretion of proinflammatory molecules	[121]
	HA	Micro/nano‐graded HA structures with appropriate pattern sizes	Polarize toward M2 macrophages	[122]
	Artificial bone bioceramic scaffold	Artificial bone bioceramic scaffold with star‐, Tai Chi‐, or interlacing‐shaped multicellular patterns	Tai Chi mode induced macrophage polarization to M2 phenotype and increased secretion of pro‐healing cytokines	[123]
	PCL	PCL‐fiber and PCL‐solid	PCL‐fiber promotes the transition from pro‐inflammatory to anti‐inflammatory during bone healing	[124]
	HA	HA coatings with different morphologies (Smooth micron‐scale rough surface and nanometer‐scale rough surface)	The nano‐scale roughness surface has the best effect on promoting M2 polarization of macrophages	[125]
	PDA	Solid PDA (sPDA), hollow PDA (hPDA), and mesoporous PDA (mPDA)	mPDA had the highest level of anti‐inflammatory and antioxidant effects	[126]
Pore size and porosity	PCL/PEG/HA	(PCL/PEG/HA) with different pore sizes (200, 400, and 600 µm)	The largest pore size scaffolds (600 µm) induce more M2 macrophage infiltration	[134]
	Electrospinning of PDO polymer	Different fiber and pore size electrospun scaffold (1, 10, and 15 µm)	The expression of M2 marker Arg1 was the highest in BMMΦ treated with 15 µm pore size scaffolds	[132]
	EF scaffolds	3D‐printed PCL scaffolds with PLLA electrospun microfibrous (3D‐M‐EF) and nanofibrous (3D‐N‐EF)	3D‐M‐EF scaffolds accelerate osteoblastic differentiation by polarizing M2 macrophages	[135]
Hydrophilicity of biomaterials' surface	Ti	Hydrophobic and hydrophilic Ti	Macrophages cultured on high surface wettability materials produce an anti‐inflammatory microenvironment	[137]
	Ti	Sand‐blasted acid‐etched (SLA) and hydrophilic SLA (modSLA) titanium surfaces	Hydrophilic surfaces result in more successful osseointegration through BMP signalling.	[138]
	Ti	Hydrophilic, micropatterned hydrophobic/hydrophilic, and hydrophobic surfaces	Hydrophilic surfaces drive the transformation of macrophages to the M2 phenotype through the PI3K/Akt signaling pathway.	[139]
Stiffness	PA gels	Different stiffness (323 kPa, 88 kPa, 11 kPa)	Gels with hardnesses of 11 kPa and 88 kPa promote an increase in IL10 production in macrophages in a ROCK dependent manner	[140]
	Agarose gel	Agarose gel (1% gel (4 kPa), 4% gel (15 kPa), and 10% hard gel (100 kPa))	The soft substrate induced the M2 macrophage polarization through the induction of PPARγ expression	[141]
	PEG‐RGD hydrogel	PEG‐RGD hydrogels were fabricated with compressive moduli of 130, 240, and 840 kPa	Hydrogels with lower stiffness led to reduced macrophage activation and FBR	[142]
Surface charge	Ti	Surface zeta potentials of Ti, Ti–P1#, and Ti–P2# were ≈−40, −80, −100 mV	The low‐potential Ti surface regulates macrophage polarization through the PI3K‐Akt signaling pathway toward M2 phenotype	[143]

**Abbreviations**: SLA: microscale sand blasted‐acid etched topography; TNT: nanoscale TiO2 nanotube topography; sPDA: solid PDA; hPDA: hollow PDA; mPDA: mesoporous PDA; PDO: polydioxanone; EF: electrospun fibrous; PA gels: polyacrylamide gels.

#### 3.1.1 Surface Roughness and Topography

The surface roughness and morphology of biomaterials play critical roles in modulating the immune response during bone healing, facilitating the creation of a favorable immune microenvironment that promotes tissue regeneration.^[^ ^115^ ^]^ The design of highly ordered microstructures on biomaterial surfaces has significant potential for enhancing immunomodulation and osteogenesis. Surface morphology, which includes features such as roughness, order/disorder, and the presence of patterned or unpatterned microstructures, directly influences cell behavior. The interaction between cells and the biomaterial surface affects key processes such as cell adhesion, migration, proliferation, and differentiation. By strategically tailoring these surface characteristics, biomaterials can be optimized to enhance cellular responses and promote efficient bone repair.

Given the substantial impact of nanoscale topography on cellular behavior, numerous studies have highlighted how alterations in nanoscale features can influence the adhesion, proliferation, and differentiation of osteoblasts. These topographical modifications, however, extend their effects beyond osteoblast regulation, significantly affecting immune cells and the surrounding immune microenvironment. For instance, research has demonstrated that nanoscale topography can modulate macrophage cytoskeletal organization, signaling pathways, and gene expression, thereby controlling macrophage morphology, adhesion, proliferation, and inflammatory responses.^[^ ^116^ ^]^ By influencing macrophage behavior in this way, the nanoscale topography indirectly impacts the activity of osteoblasts, osteoclasts, and angiogenesis, thus playing a crucial role in the overall bone healing process.^[^ ^117^ ^]^

**Figure 1:**
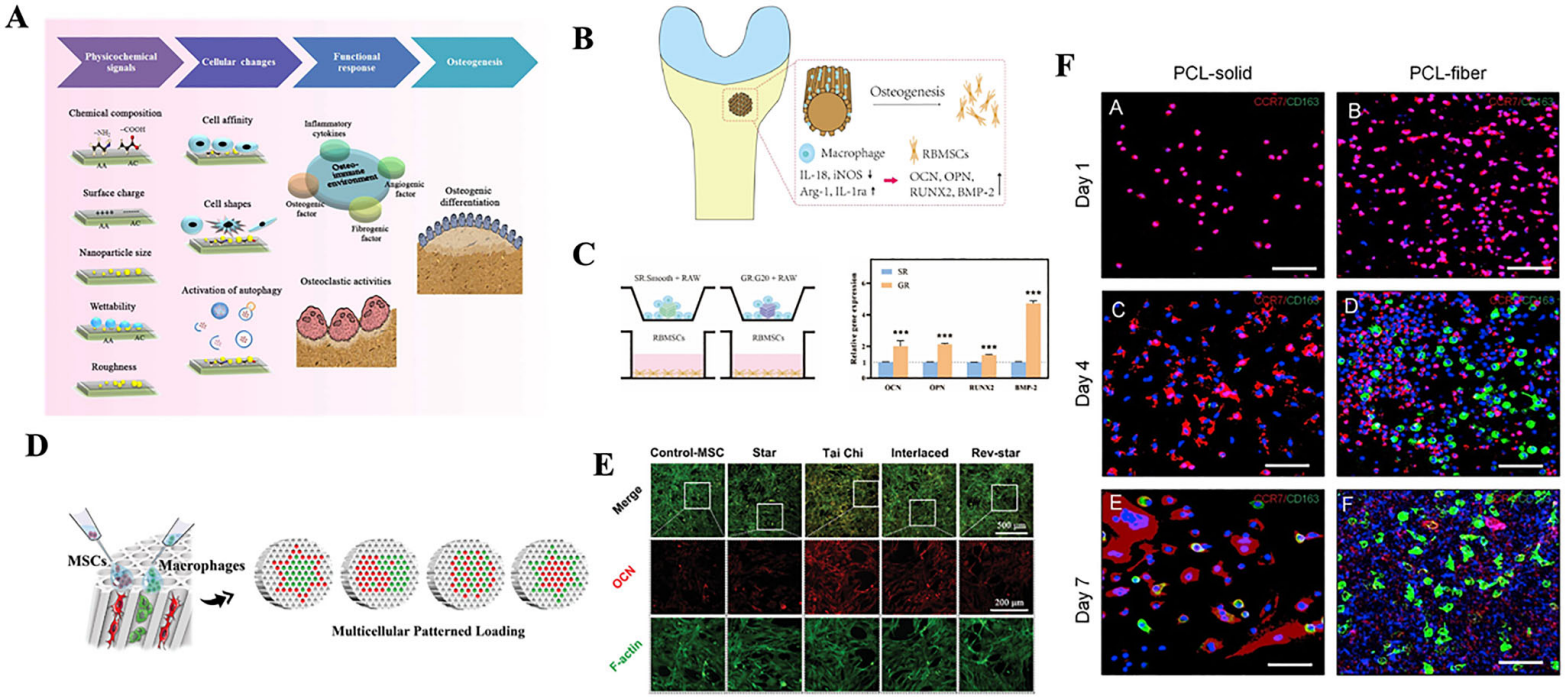
A) Tuning Chemistry and Topography of Nanoengineered Surfaces to Manipulate Immune Response for Bone Regeneration Applications. Reproduced with permission.^[^ ^119^ ^]^ Copyright 2017, American Chemical Society. B) 3D printing of gear‐inspired biomaterials: Immunomodulation and bone regeneration. C) The schematic illustration of the co‐culture of RBMSCs (on the plate) and scaffolds loaded with RAW264.7 cells (in the insert). The SR group meant that the RAW264.7 cells were seeded on the Smooth scaffolds, and the GR group meant that the RAW264.7 cells were seeded on the G20 scaffolds. The osteogenic gene expression of RBMSCs (on the plate) after co‐culturing for 2 days. (^*^ *p* < 0.05, ^**^ *p* < 0.01, ^***^ *p* < 0.001). Reproduced with permission.^[^ ^120^ ^]^ Copyright 2022, Acta Materialia Inc. Published by Elsevier Ltd. D) Characterization of the multichannel honeycomb‐like bioceramic scaffolds and patterned spatial configuration of MSCs and macrophages in the scaffolds. Schematic illustration of multicellular patterning bioceramic scaffolds. The red channel is loaded with MSCs, and the green channel means it is loaded with macrophages. E) immunofluorescent staining of OCN for MSCs cultured on scaffolds with different multicellular patterns for 7 days. The Tai Chi pattern with a 2:1 ratio of MSCs to macrophages could significantly promote the osteogenic differentiation of MSCs as compared with the other multicellular patterns. Reproduced with permission.^[^ ^123^ ^]^ Copyright 2022 The Authors. Advanced Science published by Wiley‐VCH GmbH. F) Immunofluorescence analyses on the macrophage polarization‐related markers in the cells adhered onto the PCL‐solid and PCL‐fiber at days 1, 4, and 7 post‐implantation. Fluorescence images of adhered cells, which were immunostained for CCR7 (red, M1 phenotype marker) or CD163 (green, M2 phenotype marker), were observed on PCL‐solid (A, C, and E) and PCL‐fiber (B, D, and F). Reproduced with permission.^[^ ^124^ ^]^ Copyright 2017 Elsevier Ltd. All rights reserved.

Research conducted by Chen et al. demonstrated that adjusting the surface chemistry and scale of nanostructures (16, 38, and 68 nm) can effectively create a favorable immune microenvironment at bone defect sites. Their study confirmed that the immune microenvironment regulated by nanoscale topography could enhance the osteogenic differentiation of BMSCs. Specifically, the surface chemistry of nanostructures, such as amine and acrylic acid functional groups, has been shown to influence bone immunomodulation by modulating immune cell behavior, particularly that of macrophages. Amine surfaces tended to promote anti‐inflammatory M2 macrophage phenotypes, whereas acrylic acid‐functionalized surfaces were more likely to induce proinflammatory M1 phenotypes. Additionally, the size of the nanostructures influences macrophage attachment and differentiation, thereby regulating the osteogenic process. Smaller nanostructures generally facilitate bone formation, whereas larger structures are more effective in modulating immune responses.^[^ ^118^ ^]^ (**Figure** **1**A) Nanotopography‐induced cytoskeletal reorganization activates integrin‐mediated signaling, such as PI3K/Akt, which suppresses NF‐κB and enhances β‐catenin nuclear translocation. This cascade not only polarizes macrophages toward the M2 phenotype but also synergistically activates Wnt/β‐catenin signaling in osteoblasts, bridging immunomodulation and osteogenesis. Furthermore, it was found that different nanotopological scales, in combination with surface chemistry modifications, synergistically regulate the immune microenvironment during bone regeneration. Huang and colleagues employed two different surface treatment techniques—echniques—sandblasting, large‐grit, acid‐etched (SLA) and titanium nanotubes (TNTs)—to modify titanium surfaces. TNT treatment created nanotube structures on the titanium surface, providing a larger surface area and more complex topography. These nanotubes demonstrated superior antioxidative capacity and improved osseointegration under oxidative stress conditions, which was found to be mediated by the expression or silencing of the signaling FoxO1 in MSCs.^[^ ^119^ ^]^

Additionally, some studies have demonstrated that the ordered microstructures on the surface of biomaterials can directly influence their cellular behavior. Yu and colleagues successfully fabricated a 3D ceramic scaffold with well‐ordered surface microstructures inspired by gear structures. This scaffold was shown to effectively regulate the immune response during bone regeneration. (Figure 1B) Researchers have compared bionic gear‐shaped ceramic scaffolds with different groove widths and depths (G10, G20, G50, and G100), as well as ceramic scaffolds with varying groove depths (D30, D70, and D100), alongside smooth scaffolds without gear structures. The experiments revealed that scaffolds with a gear structure featuring a groove width of 20 micrometers and a depth of 5 micrometers could induce M2 macrophage polarization, thereby providing optimal and stable anti‐inflammatory effects. Moreover, these scaffolds effectively promoted the osteogenic differentiation of rat bone marrow stromal cells (RBMSCs) under inflammatory conditions^[^ ^120^ ^]^ (Figure 1C **)**.

Moreover, research has shown that the alignment of electrospun polylactic acid (PLA) fibers—either random or oriented—significantly influences macrophage activation and osteogenesis. The results indicated that randomly aligned electrospun fibers induced macrophages to polarize toward the M1 phenotype, characterized by high expression of chemokine (C–C motif) receptor 7 (CCR7) and IL‐1β, along with increased secretion of TNF‐α and IL‐8. These findings suggest that nanoscale topography can significantly impact immune cell attachment and activation states. Surfaces with specific nanoscale structures can influence the morphological changes, phenotypic shifts, and cytokine release of immune cells through both physical contact and chemical signaling.^[^ ^121^ ^]^

Additionally, Chen et al. developed hydroxyapatite (HA) materials with micro/nanoscale hierarchical structures via a combination of photolithography and hydrothermal techniques. The micron‐sized pores provided physical support, whereas the nanoscale surface roughness influenced cell membrane deformation and signal transduction, thereby promoting macrophage activation and M2 polarization. This multiscale structure, which combines micron‐sized porosity with nanoscale surface roughness, effectively mimics the complex geometry of natural bone tissue. The authors reported that micro/nanoscale hierarchical HA structures, with appropriately sized patterns, could either promote or mitigate macrophage polarization, which in turn influences the osteogenic differentiation of human bone marrow stromal cells (hBMSCs) and the angiogenic activity of human umbilical vein endothelial cells (HUVECs).^[^ ^122^ ^]^

Additionally, cellular micropatterning has emerged as an effective immunomodulatory strategy that regulates cell behavior and function while maintaining a favorable local immune microenvironment. By mimicking the natural microstructure and functionality of bone, micropatterning can optimize both cellular interactions and tissue regeneration. Zhang et al. designed three different types of micropatterned bioceramic bone materials through the spatial arrangement of MSCs and macrophages: star‐shaped, tai chi‐shaped, and staggered patterns^[^ ^123^ ^]^ (Figure 1D). Their study demonstrated that the tai chi‐shaped pattern, in which the number of MSCs was double that of macrophages, has the most significant effects in reducing excessive inflammatory responses, resulting in uniform cell adhesion and migration pathways. This pattern was most effective in inducing M2 macrophage polarization and accelerating new bone formation, thereby creating a stable microenvironment conducive to long‐term tissue regeneration (Figure 1E).

 Furthermore, Zhang et al. compared electrospun polycaprolactone (PCL) fibers with PCL solids, noting the distinct physical morphologies and surface properties of the two materials, which led to different interactions with immune cells. The results demonstrated that PCL fibers, owing to their fibrous structure, larger specific surface area, and higher porosity, recruited more MSCs and significantly increased the population of M2 macrophages (Figure 1F). This shift in macrophage polarization contributed to a transition from a proinflammatory state to an anti‐inflammatory state during bone healing. The study concluded that the fibrous structure of biomaterials, through its ability to modulate immune responses, might be particularly beneficial for enhancing bone regeneration and osseointegration.^[^ ^124^ ^]^

Moreover, the regulation of osteoblast (OB) and osteoclast (OC) behavior is also affected by the morphology of biomaterials. Research has shown that different surface topographies—such as smooth, micron‐scale rough surfaces and nanoscale rough surfaces—of HA coatings can differentially regulate the activity of osteoblasts and osteoclasts. Specifically, HA coatings with micron‐scale roughness promote the polarization of macrophages toward the anti‐inflammatory M2 phenotype, whereas nanoscale rough surfaces further enhance this immunoregulatory effect, facilitating the maintenance of an anti‐inflammatory state by macrophages. This favorable microenvironment not only supports osteogenesis but also inhibits osteoclast activity, thereby preventing excessive bone resorption. This inhibitory effect is partially mediated by a reduction in the number of proinflammatory M1 macrophages, which typically promote osteoclast differentiation and bone resorption through the secretion of cytokines such as TNF‐α and IL‐6. In contrast, the predominance of M2 macrophages suppresses osteoclast differentiation and activity, thereby supporting bone preservation and regeneration.^[^ ^125^ ^]^

**Figure 2:**
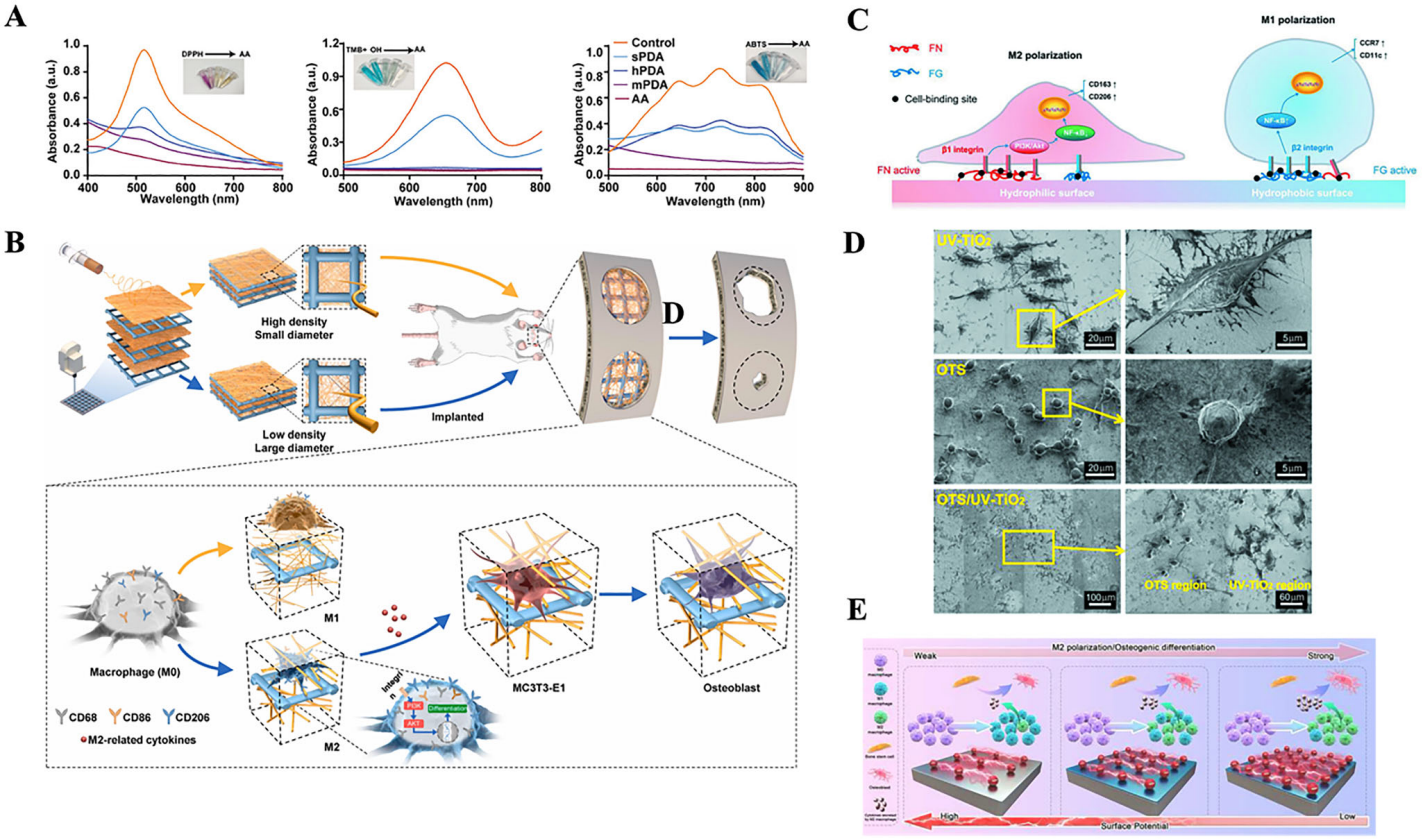
A) UV–vis absorbance spectra of DPPH·, TMB+·OH, ABTS^+·^ radicals after incubation with the PDA NPs. Reproduced with permission.^[^ ^301^ ^]^ Copyright 2023 Wiley‐VCH GmbH. B) Schematic illustration of the study. The 3D‐printed electrospun fibrous scaffolds were developed by combining 3D printing technology and electrospinning technology. The 3D‐M‐EF scaffolds polarized macrophages toward the M2 phenotype via PI3K/AKT signaling and enhanced bone regeneration. Reproduced with permission.^[^ ^135^ ^]^ Copyright 2021 Elsevier Ltd. All rights reserved. C) Schematic description illustrating the potential mechanism of surface hydrophilicity on macrophage adhesion and polarization. D) FE‐SEM observation of RAW cells after 1 d incubation on different surfaces. The images in the right column were obtained under higher magnification. Reproduced with permission.^[^ ^139^ ^]^ Copyright 2018 WILEY‐VCH Verlag GmbH & Co. KGaA, Weinheim.dr. E) Tuning the surface potential to reprogram the immune microenvironment for bone regeneration. Reproduced with permission.^[^ ^143^ ^]^ Copyright 2022 Elsevier Ltd. All rights reserved.

Elevated levels of ROS in the body can continuously trigger inflammation, thereby impairing bone healing. Therefore, the capacity of bone repair materials to scavenge ROS plays a crucial role in modulating immune responses during the bone healing process. Zheng et al. fabricated three types of nanoparticles with different morphologies: solid PDA (sPDA), hollow PDA (hPDA), and mesoporous PDA (mPDA). Their study revealed that mPDA, owing to its relatively large specific surface area and abundance of active sites, was particularly effective in promoting macrophage polarization toward the anti‐inflammatory M2 phenotype. Moreover, mPDA enhanced the production of anti‐inflammatory cytokines, such as IL‐10. As a result, mPDA exhibited the most potent antioxidant and anti‐inflammatory effects, showing significant therapeutic potential in reducing reperfusion injury, preventing periodontitis, and improving renal function (**Figure** **2**A).^[^ ^126^ ^]^

In summary, the surface morphology of biomaterials may be a critical parameter in the immunoregulation of bone healing. Smaller nanoscale structures, specific surface modifications, well‐designed microstructures, and higher specific surface areas can promote the formation of an anti‐inflammatory environment through various mechanisms, thereby supporting the generation and integration of new bone.

#### 3.1.2 Pore Size and Porosity

The pore size and porosity of bone filler materials play crucial roles in regulating cellular behavior during bone reconstruction, influencing key processes such as cell adhesion, morphological changes, and signal transduction in biomaterials.^[^ ^127^, ^128^, ^129^ ^]^ Studies have reported that pore size and porosity significantly impact the proliferation, migration, and differentiation capabilities of osteoprogenitor cells. Compared with nonporous cells, porous cells exhibit superior migration, proliferation, and differentiation. Moreover, the degradation rate of porous materials is more closely related to the natural healing process of bone.^[^ ^130^ ^]^ Additionally, during bone repair, the infiltration of biomolecules such as proteins or oxygen is closely linked to pore size, making porosity a key factor in regulating osteogenesis and the immune response. An insufficient oxygen supply within a material can lead to chronic inflammation, which further hinders bone healing and regeneration.^[^ ^131^ ^]^

During bone regeneration, the pore size and porosity of scaffolds are critical for regulating osteogenesis and immune responses. The infiltration of biomolecules such as proteins and oxygen is influenced by the degree of porosity, and an inadequate oxygen supply can induce hypoxia, leading to chronic inflammation.^[^ ^132^, ^133^ ^]^ Li et al. fabricated bioactive scaffolds with various pore sizes (200 µm, 400 µm, and 600 µm) via pneumatic extrusion‐based 3D printing technology with a composite of polycaprolactone/polyethylene glycol/hydroxyapatite (PCL/PEG/HA). Their study revealed that the scaffolds with the largest pore size recruited the fewest inflammatory cells, had the lowest M1/M2 macrophage ratio, and promoted vascularization and new bone formation. In contrast, scaffolds with smaller pore sizes tended to promote proinflammatory M1 macrophage activity, which could exacerbate the foreign body response and hinder bone tissue formation. Smaller pore sizes also contributed to hypoxic conditions within the scaffold, further enhancing M1 macrophage activity. However, larger pore sizes may not always be ideal for bone tissue engineering, as increased pore size can significantly compromise the mechanical properties of the scaffold, limiting its practical application. Therefore, selecting the optimal pore size for bone repair materials requires a careful balance between mechanical stability and immunomodulatory effects.^[^ ^134^ ^]^ Further research by Garg et al. revealed that increasing the fiber diameter or pore size of electrospun scaffolds within a smaller range (less than 20 micrometers) shifted macrophage polarization toward the M2 phenotype. This shift was marked by an increase in the expression of the M2 marker arginase 1 (Arg1) and a decrease in the expression of the M1 marker iNOS.^[^ ^132^ ^]^ The underlying mechanism may be attributed to the fact that, under conditions of larger pore sizes and higher porosity, macrophages are better able to infiltrate the scaffold and adopt a more natural and dispersed morphology, which is more conducive to tissue regeneration. Furthermore, the signals transmitted to macrophages by scaffolds with different fiber diameters/pore sizes vary, influencing the polarization of these cells. These findings suggest that myeloid differentiation primary response protein (MyD88) is a critical component involved in the signaling mechanisms driving macrophage polarization.^[^ ^135^ ^]^

Liu et al. prepared two PCL scaffolds, one containing PLLA electrospun microfibers (3D‐M‐EF) and the other containing PLLA electrospun nanofibers (3D‐N‐EF), with pore sizes of 3 and 10 µm, respectively. They reported that 3D‐M‐EF was able to increase the ratio of M2/M1 type macrophages while activating the PI3K/AKT signaling pathway^[^ ^135^ ^]^ (Figure 2B). Although larger pore sizes were beneficial for the transport of nutrients and provided channels for the inward growth of cells, scaffolds with larger pore sizes presented lower biomechanical properties. Therefore, appropriate parameters could be selected according to different clinical purposes to achieve the best immune regulation and bone repair effects.^[^ ^135^ ^]^

Precise regulation of the bone immune microenvironment not only depends on the chemical composition of the material but also influences the process of bone repair by regulating the spatial distribution, morphological changes, and signal transduction of immune cells. In addition, the average diameters of the 3D‐M‐EF and 3D‐N‐EF scaffolds were 2.61 µm and 520 nm, respectively, and the results revealed that the depth of RAW264.7 cell infiltration into the M‐EF scaffold was significantly greater than the depth of infiltration into the N‐EF scaffold. However, more RAW264.7 cells were distributed on the surface of the 3D‐N‐EF scaffold than on the 3D‐M‐EF scaffold, and MC3T3‐E1 cells inoculated on the 3D‐M‐EF scaffold were more likely to adhere to the interior of the scaffold. In addition, fiber diameters of different sizes affect the morphology and direction of polarization of macrophages. Scaffolds with larger diameters are more likely to infiltrate, whereas those with smaller diameters can make it easier for cells to grow on the surface. The nanoscale diameter of the fiber scaffold polarized macrophages toward the M1 direction, and the micron diameter of the fiber scaffold polarized macrophages toward the M2 direction, which reduced the infiltration of macrophages, accelerated tissue repair, and established a good anti‐inflammatory microenvironmentPrecise regulation of the bone immune microenvironment not only depends on the chemical composition of the material but also influences the process of bone repair by regulating the spatial distribution, morphological changes and signal transduction of immune cells. In addition, the average diameters of the 3D‐M‐EF and 3D‐N‐EF scaffolds were 2.61 µm and 520 nm, respectively, and the results revealed that the depth of RAW264.7 cell infiltration into the M‐EF scaffold was significantly greater than the depth of infiltration into the N‐EF scaffold. However, more RAW264.7 cells were distributed on the surface of the 3D‐N‐EF scaffold than on the 3D‐M‐EF scaffold, and MC3T3‐E1 cells inoculated on the 3D‐M‐EF scaffold were more likely to adhere to the interior of the scaffold. In addition, fiber diameters of different sizes affect the morphology and direction of polarization of macrophages. Scaffolds with larger diameters are more likely to infiltrate, whereas those with smaller diameters can make it easier for cells to grow on the surface. The nanoscale diameter of the fiber scaffold polarized macrophages toward the M1 direction, and the micron diameter of the fiber scaffold polarized macrophages toward the M2 direction, which reduced the infiltration of macrophages, accelerated tissue repair, and established a good anti‐inflammatory microenvironmentPrecise regulation of the bone immune microenvironment not only depends on the chemical composition of the material but also influences the process of bone repair by regulating the spatial distribution, morphological changes and signal transduction of immune cells. In addition, the average diameters of the 3D‐M‐EF and 3D‐N‐EF scaffolds were 2.61 µm and 520 nm, respectively, and the results revealed that the depth of RAW264.7 cell infiltration into the M‐EF scaffold was significantly greater than the depth of infiltration into the N‐EF scaffold. However, more RAW264.7 cells were distributed on the surface of the 3D‐N‐EF scaffold than on the 3D‐M‐EF scaffold, and MC3T3‐E1 cells inoculated on the 3D‐M‐EF scaffold were more likely to adhere to the interior of the scaffold. In addition, fiber diameters of different sizes affect the morphology and direction of polarization of macrophages. Scaffolds with larger diameters are more likely to infiltrate, whereas those with smaller diameters can make it easier for cells to grow on the surface. The nanoscale diameter of the fiber scaffold polarized macrophages toward the M1 direction, and the micron diameter of the fiber scaffold polarized macrophages toward the M2 direction, which reduced the infiltration of macrophages, accelerated tissue repair, and established a good anti‐inflammatory microenvironment.^[^ ^132^ ^]^

In conclusion, while larger pore sizes facilitate nutrient transport and cell growth, the mechanical properties of the scaffold must also be considered for successful bone repair. Therefore, optimizing pore size on the basis of clinical needs can help achieve the most effective immunoregulation and support bone regeneration.

#### 3.1.3 Hydrophilicity

The hydrophilicity of biomaterials is a crucial factor in regulating cell behavior and immune responses during bone healing and regeneration. Hydrophilic surfaces are particularly effective at promoting the adhesion, proliferation, migration, and differentiation of MSCs. This is because hydrophilic surfaces tend to activate intracellular signaling pathways, which support the functionality of cells. In addition to influencing osteogenic differentiation, hydrophilic surfaces also contribute to bone regeneration by modulating the immune system.

Hydrophilic material surfaces more readily adsorb specific serum proteins, such as fibronectin and albumin, which act as bridges for cell adhesion and directly influence the adhesion and activation of immune cells. The type and conformation of the adsorbed proteins affect how macrophages recognize and respond to the surface, thereby regulating their polarization state. Generally, bone scaffold materials with hydrophilic surfaces tend to inhibit macrophage polarization toward the M1 phenotype, resulting in reduced secretion of proinflammatory molecules and the creation of an anti‐inflammatory immune microenvironment. When macrophages are predominantly polarized toward the M2 phenotype, the anti‐inflammatory cytokines and growth factors they secrete can further stimulate the osteogenic differentiation of MSCs, thereby accelerating bone tissue formation and repair.^[^ ^136^ ^]^

Hotchkiss et al. studied titanium surfaces with varying degrees of roughness and hydrophilicity to evaluate their effects on macrophage polarization. These results indicated that hydrophilic titanium surfaces created a more favorable, anti‐inflammatory microenvironment. This was evidenced by increased levels of anti‐inflammatory cytokines, such as IL‐4 and IL‐10, which helped to reduce postimplantation inflammatory responses and accelerated osseointegration. The study also revealed that combining increased surface roughness with hydrophilicity had a synergistic effect, further enhancing immune modulation and promoting bone integration.^[^ ^137^ ^]^

Similarly, Vlacic‐Zischke et al. compared two types of titanium implant surfaces: sandblasted, large‐grit, acid‐etched (SLA) and hydrophilic SLA (modSLA) surfaces. The hydrophilic modSLA surface was shown to reduce the foreign body response around the implant and decrease the degree of fibrous encapsulation, thus promoting a more favorable immune environment. This was accompanied by increased expression of osteogenic genes, including osteocalcin, alkaline phosphatase, and osteoprotegerin, suggesting enhanced osteogenic activity. Furthermore, this study revealed the upregulation of genes and proteins within the Transforming Growth Factor‐β/bone morphogenetic protein (TGF‐β/BMP) signaling pathway, which plays a key role in bone regeneration. These findings suggest that hydrophilic materials can increase the expression of cytokine receptors in osteoblasts, further promoting bone regeneration.^[^ ^138^ ^]^

Compared with hydrophobic surfaces, hydrophilic TiO_2_ films promote a shift in macrophage polarization toward the M2 phenotype, which inhibits inflammation. The hydrophilicity of the surface influences the adsorption and conformation of specific proteins, such as fibronectin and fibrinogen. This, in turn, modulates macrophage behavior through the selective expression of integrins β1 and β2, which activate the PI3K and NF‐κB signaling pathways. On hydrophilic/hydrophobic striped surfaces (octadecyl‐richlorosilane/ultraviolet‐treated TiO_2_), macrophages exhibit filamentous attachment and clear cytoplasmic extension (Figure 2C,D). M2 macrophages secrete factors that promote the proliferation of preosteoblasts, osteogenic differentiation, and mineralization, ultimately facilitating the regeneration of bone tissue.^[^ ^139^ ^]^

The hydrophilicity of a material plays a critical role in the immunoregulation of bone regeneration. Ultrahydrophilic and highly hydrophilic surfaces are particularly effective in creating an immune microenvironment that supports bone regeneration, primarily by promoting M2 macrophage polarization and suppressing proinflammatory responses. These surface properties make such materials well‐suited for use in bone implants and tissue engineering applications. In contrast, surfaces with moderate to weak hydrophilicity, or those that are hydrophobic, may exacerbate proinflammatory reactions, which can hinder bone tissue repair and regeneration. Thus, optimizing surface hydrophilicity is a key strategy for enhancing clinical outcomes in the design of biomaterials for bone regeneration.

#### 3.1.4 Stiffness

Another critical physical parameter of bone scaffold materials is stiffness, which plays a significant role in modulating the immune response during bone repair. The stiffness of biomaterials influences various cellular behaviors, signaling pathways, and the overall bone healing process. Notably, stiffness can affect macrophage polarization and phagocytic activity, which are key factors in the immune response to bone injury. Rukmani Sridharan et al. investigated the polarization and functional behaviors of THP‐1‐derived macrophages cultured on polyacrylamide gels with varying stiffnesses. These findings revealed that stiffer materials, which induce greater cell tension and stress fiber formation, tended to promote M1 polarization, associated with proinflammatory responses. In contrast, softer materials, characterized by lower mechanical tension, favor M2 polarization and anti‐inflammatory responses. Specifically, gels with a stiffness of 323 kPa induced a proinflammatory phenotype in macrophages and decreased their phagocytic function, whereas gels with stiffnesses of 11 kPa and 88 kPa promoted an anti‐inflammatory phenotype and enhanced macrophage phagocytic activity. Additionally, stiffness‐dependent macrophage polarization is mediated through the Rho‐associated protein kinase (ROCK) signaling pathway.^[^ ^140^ ^]^

Takayuki Okamoto and colleagues examined the impact of substrate stiffness on macrophage polarization and peroxisome proliferator‐activated receptor gamma (PPARγ) expression, as well as its role in bone repair. They fabricated matrices with three different stiffness levels: 1% gel (4 kPa), 4% gel (15 kPa), and 10% stiff gel (100 kPa). Their study revealed that decreasing substrate stiffness promoted M2 macrophage polarization, enhancing immunoregulation and tissue repair by upregulating PPARγ expression. These findings offer valuable theoretical insights for the design of biomaterials for bone regeneration.^[^ ^141^ ^]^ Takayuki Okamoto and colleagues examined the impact of substrate stiffness on macrophage polarization and peroxisome proliferator‐activated receptor gamma (PPARγ) expression, as well as its role in bone repair. They fabricated matrices with three different stiffness levels: 1% gel (4 kPa), 4% gel (15 kPa), and 10% stiff gel (100 kPa). Their study revealed that decreasing substrate stiffness promoted M2 macrophage polarization, enhancing immunoregulation and tissue repair by upregulating PPARγ expression. These findings offer valuable theoretical insights for the design of biomaterials for bone regeneration.^[^ ^142^ ^]^

The mechanical stiffness of materials plays a critical role in regulating macrophage polarization by influencing cell adhesion and the distribution of stress within the cytoskeleton. Soft substrates reduce intracellular stress transmission, thereby activating signaling pathways that promote M2 polarization, whereas rigid substrates increase intracellular stress, driving M1 polarization. Therefore, controlling the stiffness of biomaterials is crucial when designing materials for bone regeneration. In addition to meeting the mechanical requirements of the target tissue, biomaterials must also effectively modulate the immune response to facilitate successful tissue repair. To better mimic the complex mechanical properties of bone tissue, materials with multilayer structures or stiffness gradients can be designed. These designs provide varying mechanical support across different regions, simultaneously modulating macrophage polarization and optimizing tissue repair outcomes. Thus, controlling the stiffness of bone regeneration materials not only ensures mechanical compatibility but also regulates the immune response, creating an optimal regenerative microenvironment. By precisely tuning material stiffness, immunomodulation during tissue repair can be enhanced, ultimately improving the success of bone regeneration.

#### 3.1.5 Surface Charge

The surface charge properties and magnitude of biomaterials can affect the interaction of biomaterials with cells, cytokines, and other bioactive factors, thereby regulating the organic immune response. Typically, macrophage membranes carry a negative charge. Consequently, positively charged biomaterials tend to induce macrophage migration and infiltration, leading to a stronger inflammatory response, whereas negatively charged bone scaffold materials can mitigate inflammation. In a study by Mei Li et al., a series of implants with varying surface potentials but similar morphologies were fabricated using polydopamine‐modified titanium surfaces to investigate their effects on bone immunomodulation. Polydopamine, which contains multiple quinone groups, has a negatively charged surface. Upon heat treatment, the phenolic hydroxyl groups in polydopamine undergo oxidation, resulting in a reduction in surface potential and an increase in the M2/M1 macrophage polarization ratio, as observed in both in vivo and ex vivo experiments (Figure 2E). Gene expression analysis revealed that the low‐potential Ti surface could modulate macrophage polarization via the PI3K‐Akt signaling pathway, thus establishing an anti‐inflammatory immune microenvironment.^[^ ^143^ ^]^ However, some studies present opposing viewpoints. Matthias Bartneck and colleagues investigated the impact of surface charge modification on inflammatory gene expression and phenotypic changes by using carboxylated and amino‐functionalized gold nanorods. These results indicated that surface charge modifications significantly influence the activation of human macrophages, with amino‐terminated groups promoting the expression of mRNA encoding anti‐inflammatory proteins.^[^ ^144^ ^]^

Although modulating the surface charge of biomaterials can enhance immune regulation in material design, the effects of surface charge on macrophage polarization remain controversial and require further investigation. As such, material design should be tailored to the specific characteristics of the biomaterial, avoiding a one‐size‐fits‐all approach. Immune responses should be carefully adjusted in accordance with clinical needs and therapeutic objectives to optimize treatment outcomes.

### 3.2 Immune Modulation by the Chemical Properties of Biomaterials

The chemical properties of metallic elements in biomaterials, as well as their degradation products, significantly influence immune regulation during bone regeneration. Metallic biomaterials are widely used in bone repair because of their exceptional mechanical properties and biocompatibility. The release of metal ions at the injury site initiates an immune response, thereby facilitating tissue regeneration. Moreover, degradation products or biologically active ions released during the breakdown of these materials play a pivotal role in modulating the immune environment in bone regeneration. These ions can directly or indirectly affect immune cell behavior and participate in immune regulation through various mechanisms. A comprehensive understanding of the chemical properties of these biomaterials and their impact on immune modulation is essential for optimizing their design, ultimately improving therapeutic outcomes in bone repair. Here, the chemical properties of biomaterials are summarized and classified according to the types of applications in **Table** **2**.

**Table 2:** Immune modulation by chemical properties of biomaterials.

Engineering parameters	Bioactive molecules	Materials	Outcome	Refs.
Metallic elements	Sr	Sr‐BGn	Sr‐BGn inhibits osteoclast differentiation through inactivation of NF‐kB/MAPK/ERK1/2 mediated NFATc1 transcription process	[152]
		Sr/Cu‐BSG cement	The dual effects of inhibition of osteoclasts and promotion of osteogenesis could synergistically reduce the expression of pro‐inflammatory genes (IL‐1β, IL‐6, and CD80) and increase the expression of anti‐inflammatory genes (IL‐1Ra, TGF‐β1, and CD206)	[151]
	Mg	MgO NPs	Favor the M2 phenotype of the macrophages	[154]
		GelMA‐BP‐Mg	Beneficial to osteogenesis and angiogenesis by stimulating osteoblasts and endothelial cells while restraining osteoclasts	[158]
	Mn	Mn‐TCP	Activation of Nrf2 regulates antioxidant enzymes to clear ROS to inhibit RANKL‐induced osteoclast production	[159]
	Cu	Cu@PDA‐GOx	Favor the M2 phenotype of the macrophages	[165]
	Zn	Zn‐Coated SPEEK	Polarizing toward M2 macrophages and secreted more anti‐inflammatory cytokines	[167]
		GelMA/Met@ZIF‐8	ZIF‐8 reduces the infiltration of inflammatory cells and promotes dysfunctional mitochondria degradation by autophagy, to prevent cell damage.	[168]
	Fe	IONPs	IONPs regulated bone metabolism by scavenging ROS and promoted the osteogenic differentiation of BMSCs and inhibited the osteoclast differentiation of monocytes	[169]
		Fe‐cat NPs	Fe‐cat NPs resist inflammation and promote M2 polarization of macrophages	[170]
	Li	LiCl	LiCl promotes M2 polarization of macrophages and releases anti‐inflammatory	[171]
		Li‐gel	Li‐gel favored the M2 phenotype of the macrophages and osteogenesis	[49]
Degradation products in biomaterials	Bioactive glasses	BGs	The dissolution product of BGs promotes communication between immune cells, and stimulates the production of cytokines and chemokines	[173]
	silicate bioceramics	silicate bioceramics	Inhibit the activation of inflammation‐related MAPK/NF‐κB signaling pathway	[174]
	UsCCP	UsCCP	Favor the M2 phenotype of the macrophages and reduce inflammation	[175]

**Abbreviations**: Sr‐BGn:nanoscale glass powders containing Sr;SPEEK:sulfonated polyetheretherketone; IONPs: iron oxide nanoparticles; Fe‐cat NPs: iron‐catechin nanoparticles; BGs: bioactive glasses; UsCCP: ultra‐small‐sized calcium phosphate nanoclusters

#### 3.2.1 Metallic Elements

Metallic orthopedic implants or bone graft materials, which contain metallic elements, gradually degrade in the body, releasing metal ions or particles. These degradation products can activate or suppress specific immune cells, thereby regulating inflammatory responses and influencing the activity of osteoclasts, osteoblasts, and the production of angiogenesis‐related factors. Metal ions play a key role in regulating inflammation, promoting bone cell function, and facilitating vascular regeneration by interacting with cellular signaling pathways. Research has shown that metallic elements in scaffold materials contribute to bone regeneration by either releasing ions or generating electrochemical gradients. Additionally, they can modulate macrophage polarization and enhance the immune microenvironment of bone tissue through various mechanisms.^[^ ^30^, ^145^, ^146^, ^147^, ^148^ ^]^ By investigating the immune modulation mechanisms of metallic biomaterials during bone repair, their design can be optimized to improve clinical efficacy and safety.

##### Sr

Osteoclast activity is tightly regulated by various cytokines and signaling pathways within the immune system. One of the most important mechanisms is the RANK/RANKL/OPG signaling pathway, which controls osteoclast differentiation and activation. Immune cells, such as T cells and macrophages, promote osteoclast formation and activity through the secretion of RANKL. Consequently, immunoregulation is crucial for maintaining the functional balance between osteoclasts and osteoblasts. For example, strontium ranelate has been found to reduce bone resorption by increasing osteoblast activity, leading to bone formation, and deactivation of osteoclasts.^[^ ^149^ ^]^ Inspired by this, strontium‐loaded biomaterials have been developed to release strontium ions gradually, reducing systemic drug toxicity while exerting a dual regulatory effect on osteoblasts and osteoclasts in bone metabolism.^[^ ^150^ ^]^

**Figure 3:**
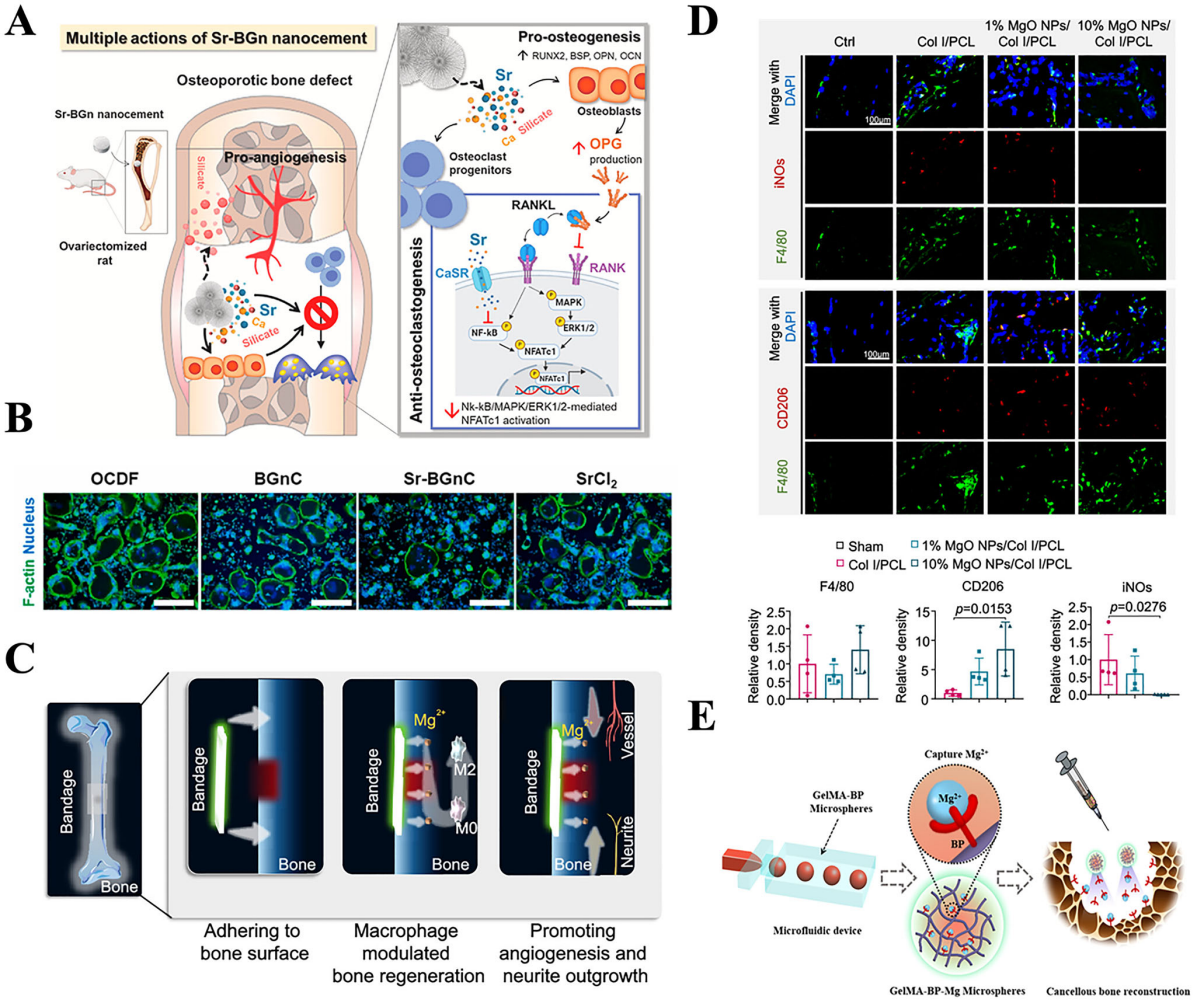
A) Dual actions of osteoclastic‐inhibition and osteogenic‐stimulation through strontium‐releasing bioactive nanoscale cement imply biomaterial‐enabled osteoporosis therapy. B) Formation of actin rings stained by Phalloidin at day 6 (scale bar = 175 µm). Reproduced with permission.^[^ ^152^ ^]^ Copyright 2021 Elsevier Ltd. All rights reserved. C) After the in vivo implantation, the periosteum bandage was adherent to mice's femoral bone surface for releasing Mg^2+^. Mg^2+^ modulated bone formation by directly activating M2 polarization of macrophages and facilitating angiogenesis and neurite outgrowth. These joint efforts accelerated the healing of the bone defect. D) Immunofluorescent staining of M1 (iNOs and F4/80) and M2 (CD206 and F4/80) macrophages after the implantation of bandages for 2 weeks and the statistical analysis of the distribution within the newly formed bone. Reproduced with permission.^[^ ^154^ ^]^ Copyright 2022, American Chemical Society E) Construction of a Microfluidic GelMA‐BP‐Mg Microsphere with a “Magnet” Function That Can Capture Mg^2+^ and Description of the Principle of Capturing Mg^2+^_._ Reproduced with permission.^[^ ^158^ ^]^ Copyright 2021, American Chemical Society.

Researchers have developed Sr‐doped bioactive nanocement (Sr‐BGnC),^[^ ^151^ ^]^ (**Figure** **3**A), which was experimentally shown to have the highest osteogenic transcriptional activity for Runt‐related transcription factor 2 (Runx2) and osteocalcin (OCN). Additionally, the expression of osteoclast‐related genes was inhibited through the modulation of osteoblast signaling, and the formation of actin rings in osteoclasts was significantly reduced (Figure 3B). The NF‐kB/MAPK/ERK1/2‐mediated activation of NFATc1 is a well‐known signaling pathway for osteoclast differentiation, which serves as a bridge between the immune system and bone metabolism. Sr‐doped bioactive nanocement (Sr‐BGnC) inhibits osteoclast differentiation by deactivating the NF‐kB/MAPK/ERK1/2‐mediated transcription of NFATc1. Therefore, Sr‐containing biomaterials can suppress osteoclast differentiation by regulating immunomodulatory factors, thus promoting bone regeneration.^[^ ^152^ ^]^

In addition to regulating osteoblast and osteoclast activity, strontium also exerts anti‐inflammatory effects. Li and colleagues developed borosilicate bone cement incorporating both strontium (Sr) and copper (Cu). The incorporation of these elements reduced the setting temperature of the bone cement, while their synergistic release promoted macrophage polarization from the proinflammatory M1 phenotype to the anti‐inflammatory M2 phenotype. This transition led to a significant reduction in the expression of proinflammatory genes (e.g., IL‐1β, IL‐6, and CD80) in RAW264.7 macrophages, whereas the expression of anti‐inflammatory genes (e.g., TGF‐β1 and CD206) was markedly increased. This not only minimized tissue damage caused by inflammation but also fostered a more favorable environment for bone regeneration, particularly during the early stages of healing, by mitigating the detrimental effects of excessive inflammation on osteogenesis.

##### Mg

For the regulation of cortical bone periosteum‐based bone regeneration,^[^ ^153^ ^]^ artificial periosteal bandages based on magnesium oxide (MgO) nanoparticles effectively regulate cortical bone regeneration (Figure 3C). In the group implanted with bandages containing MgO nanoparticles, an increase in the number of CD206‐positive M2 macrophages and a decrease in the number of iNOS‐positive M1 macrophages were detected, as was an increase in the expression of osteogenesis‐related factors. (Figure 3D). Furthermore, the expression of osteogenesis‐related genes were upregulated. These findings suggest that MgO nanoparticles can effectively regulate the behavior of periosteal cells, which are pivotal in bone regeneration. By modulating the immune response, MgO‐based materials increase the activity of periosteal cells, promoting their differentiation into osteoblasts and facilitating cortical bone formation.^[^ ^154^ ^]^

In addition to influencing periosteal cells, Mg has been shown to regulate osteoclast activity and promote the production of angiogenesis‐related factors, further contributing to an immunological microenvironment favorable for osteogenesis. However, it is important to note that both excessively high and low concentrations of Mg^2^⁺ ions can hinder osteogenic differentiation.^[^ ^154^, ^155^, ^156^, ^157^ ^]^ In addition to cortical bone regeneration, Mg^2^⁺ also plays a crucial role in cancellous bone regeneration. Zhao et al. developed a microfluidic microsphere designed to trap Mg^2^⁺ ions via a Schiff base reaction and aldehyde activation for metal ion coordination. These microspheres effectively capture and slowly release Mg^2^⁺, while bisphosphonate (BP) is targeted specifically to bone tissue (Figure 3E). In vitro experiments revealed that Mg^2^⁺‐loaded composite microspheres promote osteogenesis and angiogenesis by stimulating osteoblasts and endothelial cells while simultaneously inhibiting osteoclast activity. This dual effect allows for sustained regulation of the immune microenvironment at the site of bone regeneration. The controlled release of Mg^2^⁺ ions optimizes the local immune response, thereby enhancing the osteogenic process and supporting the regeneration of cancellous bone.^[^ ^158^ ^]^

##### Mn

**Figure 4:**
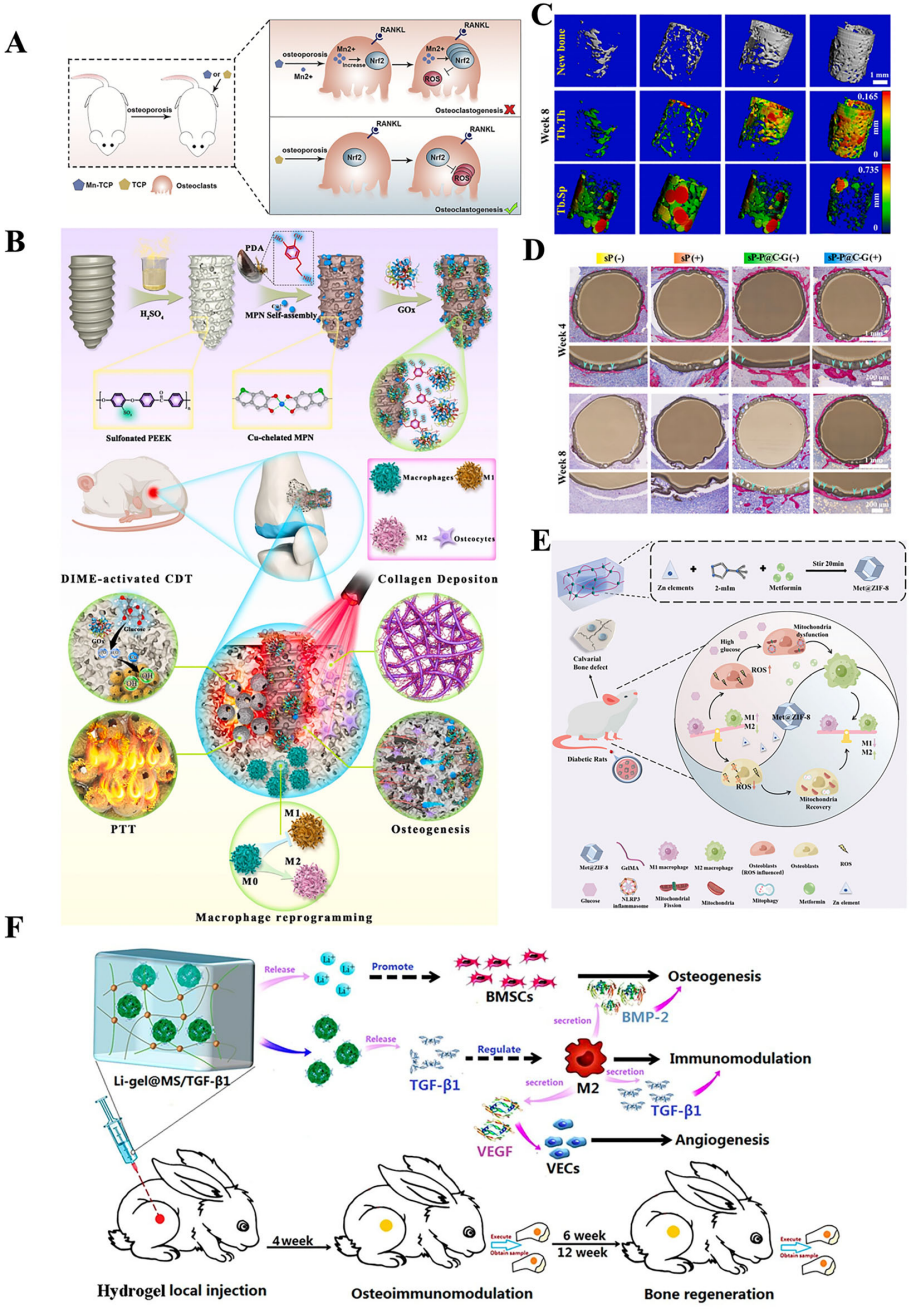
A) A schematic diagram showing the function of Mn‐TCP bioceramics in osteoporotic bone defect regeneration. Reproduced with permission.^[^ ^159^ ^]^ Copyright 2021 The Authors. B) Schematic illustration of the preparation the sterilization, and osseointegration effects of hyperglycemic micromilieu‐unlocked PEEK implants. C)Toluidine blue‐fuchsine staining showing the newly generated bone tissue around different PEEK implants at week 4 and week 8 (Green arrows indicate new bone contact with the implant surface directly). D) Micro‐CT reconstruction image of newly‐regenerated bone, Tb,·Th, and Tb. Sp around different PEEK implants at week 8 postoperatively. Reproduced with permission.^[^ ^165^ ^]^ Copyright 2023 Elsevier Ltd. All rights reserved. E) Schematic representation of how dose Met@ZIF‐8 breaks “the vicious cycle” in the diabetic microenvironment. Reproduced with permission.^[^ ^168^ ^]^ Copyright 2023 Wiley‐VCH GmbH. F) Schematic representation of the fabrication of the TGF‐β1‐containing heparin‐gelatin microspheres. Reproduced with permission.^[^ ^49^ ^]^ Copyright 2022 Elsevier Ltd. All rights reserved.

Manganese (Mn) is a potent promoter of bone healing and plays a critical role in osteoblast proliferation and adhesion during bone regeneration by activating integrins. Mn also supports osteogenic differentiation and inhibits osteoclast activity. Additionally, Mn exerts anti‐inflammatory effects by scavenging excess ROS in the body. In a study in which tricalcium phosphate (TCP) was used as the matrix, Mn was incorporated into the material to create a Mn‐doped bioceramic scaffold (Mn‐TCP) (**Figure** **4**A). The results demonstrated that Mn‐TCP effectively clears ROS by activating nuclear factor erythroid 2‐related factor 2 (Nrf2), which regulates the expression of antioxidant enzymes, thus inhibiting the production of osteoclasts induced by RANKL. RANKL is a critical immunomodulatory factor that promotes osteoclastogenesis by mediating the expression of tartrate‐resistant acid phosphatase (TRAP) through the RANK receptor. Moreover, Mn‐TCP inhibited the expression of osteoclast‐specific genes, including NFATc1, matrix metalloproteinase 9 (MMP9), dendritic cell‐specific transmembrane protein (DC‐STAMP), osteoclast stimulatory transmembrane protein (OC‐STAMP), TRAP, and cathepsin K (Ctsk), all of which are induced by RANKL. Furthermore, Mn^2^⁺ ions upregulated the expression of osteogenic‐related genes during MC3T3‐E1 osteogenic differentiation and accelerated the formation of calcium nodules.^[^ ^159^ ^]^

##### Cu

Additionally, copper activates the AKT (protein kinase B) signaling pathway in macrophages, promoting their polarization toward the M2 phenotype,^[^ ^160^ ^]^ which is a potent promoter of immunomodulation during bone repair. This, in turn, enhances both bone regeneration and angiogenesis.^[^ ^161^, ^162^, ^163^, ^164^ ^]^ Researchers have developed a bone scaffold material for treating bone defects in a glucose‐infected microenvironment that combines polyether ether ketone (PEEK), copper‐chelated metal polyphenol networks, and glucose oxidase (GOx) (Figure 4B). The scaffold demonstrated promising in vivo effects on bone regeneration (Figure 4C,D). In this system, glucose oxidase decomposes glucose in the microenvironment, which copper catalyzes the production of bactericidal hydroxyl radicals (·OH) from hydrogen peroxide (H_2_O_2_) via a Fenton‐like reaction. RNA sequencing (RNA‐Seq), quantitative real‐time polymerase chain reaction (qRT‒PCR), and other analyses revealed that the scaffold exhibited immune‐regulatory capabilities. It significantly reduces the expression of proinflammatory cytokines (TNF‐α, IL‐1β, and IL‐6) while markedly upregulating the expression of M2 macrophage markers.^[^ ^165^ ^]^

##### Zn

In addition, zinc has been shown to inhibit macrophage polarization toward the proinflammatory M1 phenotype and reduce the secretion of proinflammatory cytokines.^[^ ^166^ ^]^ Liu et al. demonstrated that sulfonated polyether ether ketone (SPEEK) coated with zinc effectively modulates macrophage polarization. Specifically, zinc‐coated SPEEK promoted the polarization of nonactivated macrophages toward the M2 phenotype, leading to increased secretion of anti‐inflammatory cytokines and increased osteoblast activity.^[^ ^167^ ^]^

In addition, Zn has been reported to have an important function in protecting mitochondria from an excess ROS environment. A biomaterial that can promote bone regeneration in an abnormal diabetic bone microenvironment was prepared by Lao et al. Zeolite imidazolium ester framework 8 (ZIF‐8) was loaded with metformin, which has the property of adapting to the pH value specific to diabetic bone defects, to release Zn^2+^ (Figure 4E). Reports have shown that it can repair dysfunctional mitochondria and reduce the infiltration of inflammatory cells, indicating that it has promising applications in diabetic bone damage repair.^[^ ^168^ ^]^

##### Fe

Zheng et al. demonstrated that iron oxide nanoparticles (IONPs) can mitigate bone loss in ovariectomized mice by effectively inhibiting osteoclast activity and exhibiting potent antioxidant properties.^[^ ^169^ ^]^ Additionally, research has shown that iron acts as an immunomodulator, influencing macrophage polarization. When iron is combined with polyphenolic compounds to form iron‐catechin nanoparticles (Fe‐cat NPs), these nanoparticles exhibit both immunomodulatory and bone‐enhancing effects. The Fe‐cat NPs reshaped the osteoimmune microenvironment by attenuating inflammation and promoting the polarization of macrophages toward the M2 phenotype, thereby facilitating bone regeneration.^[^ ^170^ ^]^

##### Li

Research findings indicate that lithium (Li) plays a pivotal role in bone tissue metabolism and systemic immune modulation, which are essential for bone regeneration and reconstruction. Specifically, Li inhibits glycogen synthase kinase 3 beta (GSK3β), which leads to decreased transcriptional activity of NF‐κB. This inhibition promotes the upregulation of the anti‐inflammatory cytokine IL‐10 by monocytes, while concurrently reducing the levels of proinflammatory cytokines such as IL‐1β, IL‐6, TNF‐α, IL‐12, and IFN‐γ. Moreover, Li enhances the osteogenic differentiation of BMSCs through activation of the Wnt/GSK‐3β signaling pathway, a critical regulator of bone formation and repair. This function of Li is particularly instrumental in fracture healing.

Investigations have demonstrated the immunomodulatory properties of lithium chloride (LiCl), particularly with respect to alterations in the macrophage phenotype induced by titanium metal particles and their subsequent enhancement of osteogenic differentiation in MSCs. The evidence suggests that an optimal concentration of LiCl facilitates the differentiation of macrophages into the M2 phenotype, thereby increasing the release of anti‐inflammatory and osteogenic factors.^[^ ^171^ ^]^

Additionally, Li et al. developed an injectable lithium‐heparin hydrogel (Li‐gel), which functions as both a microsphere delivery carrier and a sustained release system for Li ions. This innovative system effectively modulates the osteoimmune environment, fostering macrophage polarization and promoting osteogenic differentiation of BMSCs. Consequently, Li plays a crucial role in immune modulation and bone dynamics at sites of bone injury, thereby supporting effective bone repair^[^ ^49^ ^]^ (Figure 4F).

#### 3.2.2 Degradation Products

Certain bone repair materials significantly influence immunoregulation and anti‐inflammatory responses during bone regeneration through their degradation products or released ions. Upon implantation, bone repair biomaterials naturally degrade, releasing a variety of bioactive products, primarily consisting of inorganic ions and organic molecules, which exert multifaceted immunomodulatory effects within the local microenvironment. Silicate‐ and phosphate‐based bioceramics, along with bioactive glasses, contain ions such as Ca^2^⁺, Si⁴⁺, P⁵⁺, and Mg^2^⁺, which are essential for bone regeneration. As these materials degrade, these ions are gradually released and absorbed by surrounding cells, directly influencing the behavior of macrophages, osteoblasts, and fibroblasts. Additionally, certain bioceramic materials can alter local pH levels during degradation. For example, the degradation of calcium phosphate biomaterials reduces the surrounding pH, which significantly impacts macrophage activity. A lower pH environment enhances macrophage phagocytosis and promotes M2 polarization, thereby mitigating excessive inflammation and fibrosis, and supporting bone tissue repair and regeneration. Furthermore, some biomaterials release organic molecules, such as protein fragments, hyaluronic acid, and heparin, during degradation. Heparin, a natural anticoagulant, can further enhance bone formation by modulating the activity of inflammatory cytokines and growth factors.

Bioactive glass (BG) triggers immune responses upon implantation, enhancing the interaction between immune cells.^[^ ^172^ ^]^ BG primarily promotes cellular activity during tissue regeneration through its dissolution products, including released ions and induced biomineral precipitates. These products stimulate immune cells to secrete cytokines and chemokines, thereby creating an immune microenvironment conducive to the osteogenic differentiation of stem cells.^[^ ^173^ ^]^

Additionally, the ionic products released during the degradation of silicate bioceramics play crucial roles in regulating inflammation, immune cell responses, and osteogenesis. Huang and colleagues cultured RAW264.7 macrophages on Ackermannite (AKT), Nagelschmidt (NAGEL), and β‐tricalcium phosphate (β‐TCP) bioceramics. They reported that macrophages cultured on silicate ceramics secreted significantly fewer inflammatory cytokines (TGF‐β1, IL‐1β, and IL‐6) than those cultured on β‐TCP. Moreover, the Si, Mg, and Ca ions released during the degradation of silicate ceramics were shown to inhibit the MAPK/NF‐κB signaling pathways, which are associated with inflammation.^[^ ^174^ ^]^

In a related study, Zhou et al. utilized a bone filler material consisting of ultrasmall calcium phosphate nanoclusters (UsCCP) as the scaffold. Research has demonstrated that the bioactive scaffolds release substantial amounts of products, including ions, functional groups, and biomolecules, into the surrounding microenvironment. These products significantly influence the proliferation of RAW 264.7 macrophages. Additionally, the expression of CD11c was downregulated, whereas CD163 expression was upregulated. Further analyses via RT‒PCR and Western blot assays revealed increased expression of genes associated with the M2 macrophage phenotype, including Arg‐1, IL‐4, IL‐10, and interleukin‐1 receptor antagonist (IL‐1Ra), in RAW 264.7 cells cultured in the presence of UsCCP.^[^ ^175^ ^]^

### 3.3 Delivery of Bioactive Molecules by Biomaterials

In addition to the physicochemical properties of the bone filler materials themselves, which can participate in immune regulation during bone healing, they can also serve as local delivery vehicles for bioactive factors,^[^ ^176^ ^]^ to regulate the crosstalk between immune cells and stem cells. The phenotype, quantity, and function of immune cells, as well as the type, level, and effect of immune factors in the osteoimmune microenvironment, are altered, thereby promoting the process of bone regeneration (**Table** **3**).^[^ ^177^ ^]^

**Table 3:** Delivery of bioactive molecules by biomaterials.

Engineering parameters	Bioactive molecules	Materials	Outcome	Refs.
proteins/cytokines	Fg	Fg‐3D scaffolds	Increased T cells and decreased B, NK, and NKT lymphocytes and myeloid cells	[178]
	IL‐4	GO‐CMC/PEGDA IPN hydrogel	Polarize toward M2 macrophages while producing anti‐inflammatory cytokines	[179]
	FAP	FAPi‐MMS‐Gel	Eliminating ROS, polarizing toward M2 macrophages, promoting osteogenic differentiation, and inhibiting osteoclast formation.	[181]
drugs	ALN	MCPC/HMSNs@ALN‐PTH/GM	Inhibition of NF‐κB/MAPK/ p38‐mediated NFATC1 transcription inhibits osteoclastogenesis	[188]
	PCA	PCA	Polarize toward M2 macrophages while producing anti‐inflammatory cytokines	[189]
	Res	RES@PLGA NBs	Osteogenic differentiation in vitro, and significantly reduced local inflammation and enhanced bone regeneration in vivo	[191]
	Gastrodin	Gastrodin‐PU/n‐HA scaffolds	Inducing the polarization of M2 macrophages and accelerating the repair of diabetic bone defects	[192]
	EGCG	PCL‐Si	Polarize toward M2 macrophages while producing anti‐inflammatory cytokines	[193]
	DEX	Hierarchical MBG scaffolds	Reduce macrophage over‐infiltration and the transition from M1 to M2	[194]
	Puerarin	P@C	Promoted M2 macrophage polarization and increased anti‐inflammatory factor levels	[195]
	Cur	cFMSN	induce macrophage M2‐type polarization and anti‐inflammatory cytokine	[196]
	rapamycin	PSeR	Remove the accumulated ROS inside the senescent BMSCs and enhance the osteogenic potential of BMSCs	[198]
Delivery of peptides	SVVYGLR peptide	SVVYGLR peptide	Inhibit osteoclast activation and regulate osteoblast function through immune response‐related signaling pathways	[202]
	LL37 peptide W9 peptide	LL37‐W9/PLGA‐SIS	Promoted M2 macrophage polarization and increased anti‐inflammatory factor levels	[203]

**Abbreviations**: Fg: fibrinogen; IL‐4: Interleukin‐4; CMC: carboxymethyl chitosan; PEGDA: Poly(ethylene glycol) diacrylate; FAP: Fibroblast activation protein; ALN: Alendronate sodium; PCA: protocatechuic aldehyde; Res: Resveratrol; PU: polyurethane; EGCG: Epigallocatechin gallate; Dex: Dexamethasone; Cur: Curcumin; W9: WP9Q; PLGA: polylactic‐glycolic acid; SIS: small intestinal submucosa.

#### 3.3.1 Proteins/Cytokines

Fibrinogen (Fg) is a blood protein with numerous Mac‐1 binding sites that effectively activate immune cells. It also contains two arginine–glycine–aspartic acid (RGD) motifs that promote cell adhesion. While the solubility of Fg is essential for its functionality, its free form can lead to tissue damage. To mitigate this issue, researchers developed a fibrinogen‐based 3D scaffold using freeze‐drying techniques to reduce its solubility. Compared with repair without these scaffolds, tissue repair with the Fg‐3D scaffolds resulted in reduced plasma levels of IL‐1β and increased levels of TGF‐β1. Additionally, Fg‐3D scaffolds not only regulate local inflammatory cytokines but also contribute to systemic immunomodulation.^[^ ^178^ ^]^

IL‐4 is a cytokine that induces macrophage polarization toward the M2 phenotype. However, free cytokines are rapidly metabolized in vivo, which limits their effectiveness. To address this, biomaterials are often used as carriers for sustained delivery, ensuring a stable microenvironment at the site of bone injury to promote repair. Zhou et al. developed a controlled release system based on graphene oxide (GO) that codelivers IL‐4 and BMP‐2, allowing for the gradual release of bioactive factors. This system facilitates macrophage polarization toward the M2 phenotype, reducing inflammation and promoting osteoblast proliferation and differentiation through the secretion of growth factors. By regulating the immune microenvironment at various stages of bone regeneration, this sustained modulation helps maintain a balanced immune response over time, ultimately enhancing bone defect repair.^[^ ^179^ ^]^

**Figure 5:**
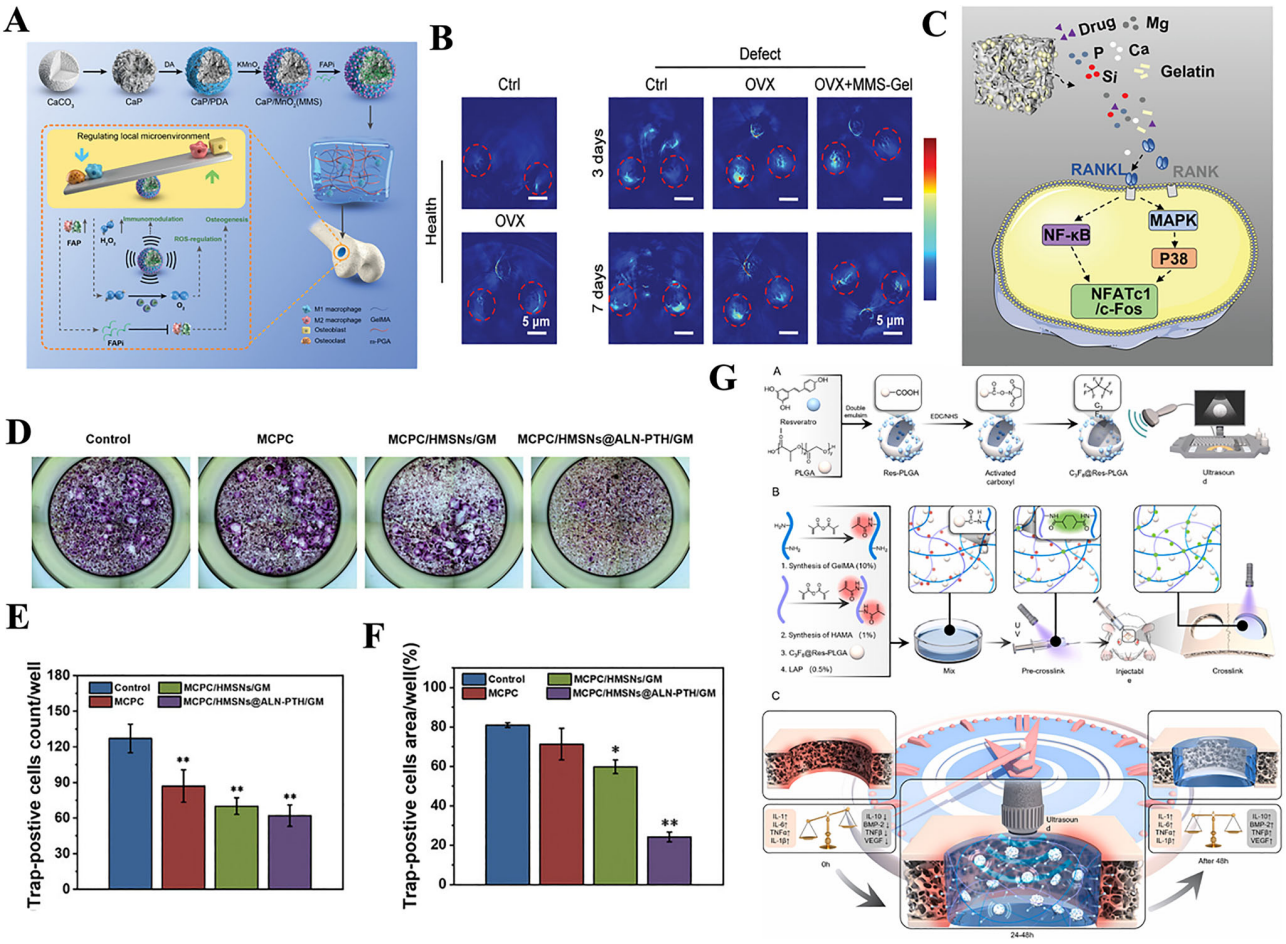
A) Schematic illustration of the design and preparation of a composite hydrogel containing MnO_2_‐coated CaP microspheres loading FAPi for the repair of osteoporotic bone defects. MnO_2_ on the surface of MMS can eliminate ROS and, accordingly, generate oxygen in the microenvironment. CaP microspheres slowly degrade and constantly release FAPi to inhibit FAP, which could regulate immune response and bone formation. B) PA images of the femur in normal (Ctrl) and OVX mice and PA images of the femur in normal (Ctrl) and OVX mice after bone injury. Reproduced with permission.^[^ ^180^ ^]^ Copyright 2022 Wiley‐VCH GmbH. C) Schematic illustration of the regulating effect of MCPC/HMSNs@ALN‐PTH/GM on osteoclastogenesis. D) TRAP staining was performed to detect osteoclast formation under the treatment of MCPC composites extracted solutions. E) The count and. F) The area of TRAP‐positive cells was statistically analyzed to evaluate the function of MCPC composites (mean ± SD; *n* = 4; ^*^ *p* < 0.05; ^**^ *p* < 0.01). Reproduced with permission.^[^ ^189^ ^]^ Copyright 2023 Wiley‐VCH GmbH. G) Schematic diagram of UCE hydrogels to precisely regulate spatiotemporal osteoimmune disturbance. Reproduced with permission.^[^ ^191^ ^]^ Copyright 2023 Elsevier Ltd. All rights reserved.

Fibroblast activation protein (FAP) plays a role in activating the inflammation‐related transcription factor NF‐κB, which is associated with the inhibition of osteoclastogenesis and thus acts as an osteogenesis inhibitor. While FAP is rarely expressed in healthy tissues, its expression is significantly upregulated during tissue injury and excessive inflammation. Moreover, FAP inhibitors (FAPi) can effectively suppress FAP production. Chen and colleagues developed a composite hydrogel made from methyl acrylate PGA, and methyl acrylate gelatin, loaded with MnO_2_ and FAPi‐calcium phosphate microspheres, which demonstrated excellent antioxidant and immune regulatory properties^[^ ^180^ ^]^ (**Figure** **5**A). The hydrogel limited osteoclast formation, as evidenced by TRAP staining and F‐actin staining, and reduced expression of osteoclast‐specific genes such as nuclear factor of activated T‐cells, cytoplasmic 1(Nfatc1), acid phosphatase 5, tartrate resistant (Acp5), and Mmp9. This prevents excessive osteoclast activation and promotes MSC differentiation toward osteogenesis. Additionally, the material exhibited strong ROS clearance ability in vivo (Figure 5B) and induced macrophage polarization toward the M2 phenotype.^[^ ^181^ ^]^

#### 3.3.2 Drugs

The modulation of the immune system by small molecule drugs or pharmacological agents during bone repair has gained significant attention in recent years.^[^ ^182^, ^183^ ^]^ Alendronate sodium (ALN) is commonly used to treat osteoporosis by inhibiting osteoclast activity and reducing bone resorption,^[^ ^184^, ^185^, ^186^, ^187^ ^]^ whereas recombinant human parathyroid hormone (rPTH 1–34), an FDA‐approved anabolic drug, enhances calcium regulation and bone remodeling in osteoporosis treatment. Zhao et al. reported that the combination of these two drugs resulted in improved osteogenic effects. They used gelatin‐coated hollow mesoporous silica nanoparticles (HMSNs/GM) in combination with magnesium calcium phosphate bone cement (MCPC), as a drug delivery carrier to continuously release both drugs at the site of bone injury. When bone marrow‐derived macrophages (BMMs) from Institute of Cancer Research (ICR) mice were cultured in vitro and treated with the drug‐loaded carriers, the results revealed significant inhibition of protein kinase phosphorylation associated with the activation of theNF‐κB and MAPK signaling pathways (Figure 5C). Moreover, there was a marked downregulation of osteoclast markers, such as MMP9 and TRAP^[^ ^188^ ^]^ (Figure 5D–F).^[^ ^189^ ^]^ By regulating immune signaling pathways, this approach influences bone tissue homeostasis. In particular, osteoclastogenesis and differentiation are suppressed by deactivation of the NF‐κB/MAPK/P38‐mediated transcription of NFATC1. This in turn modulates macrophage polarization and the interactions between immune cells, contributing to a favorable bone regeneration microenvironment.

Resveratrol (Res), a plant‐based anti‐inflammatory small molecule, has been shown to reduce inflammation by inhibiting proinflammatory factors such as TNF‐α and IL‐1β.^[^ ^190^ ^]^ Han et al. developed a novel delivery system by loading Res into poly (lactic‐co‐glycolic acid) (PLGA) to create nanobubbles. These nanobubbles were engineered to release the drug in a controlled manner at specific times and locations upon noninvasive ultrasound stimulation. The carboxyl groups of the nanobubbles were covalently bonded to the amino groups of Gelatin methacryloyl (GelMA), forming a hydrogel that allowed for ultrasound‐triggered burst release of Res. This system aligns with the spatial and temporal needs for immunomodulation during bone repair. On the second day after bone repair, the application of controlled ultrasound intensity increased the release rate of Res at the injury site, effectively suppressing immune inflammation^[^ ^191^ ^]^ (Figure 5G).

**Figure 6:**
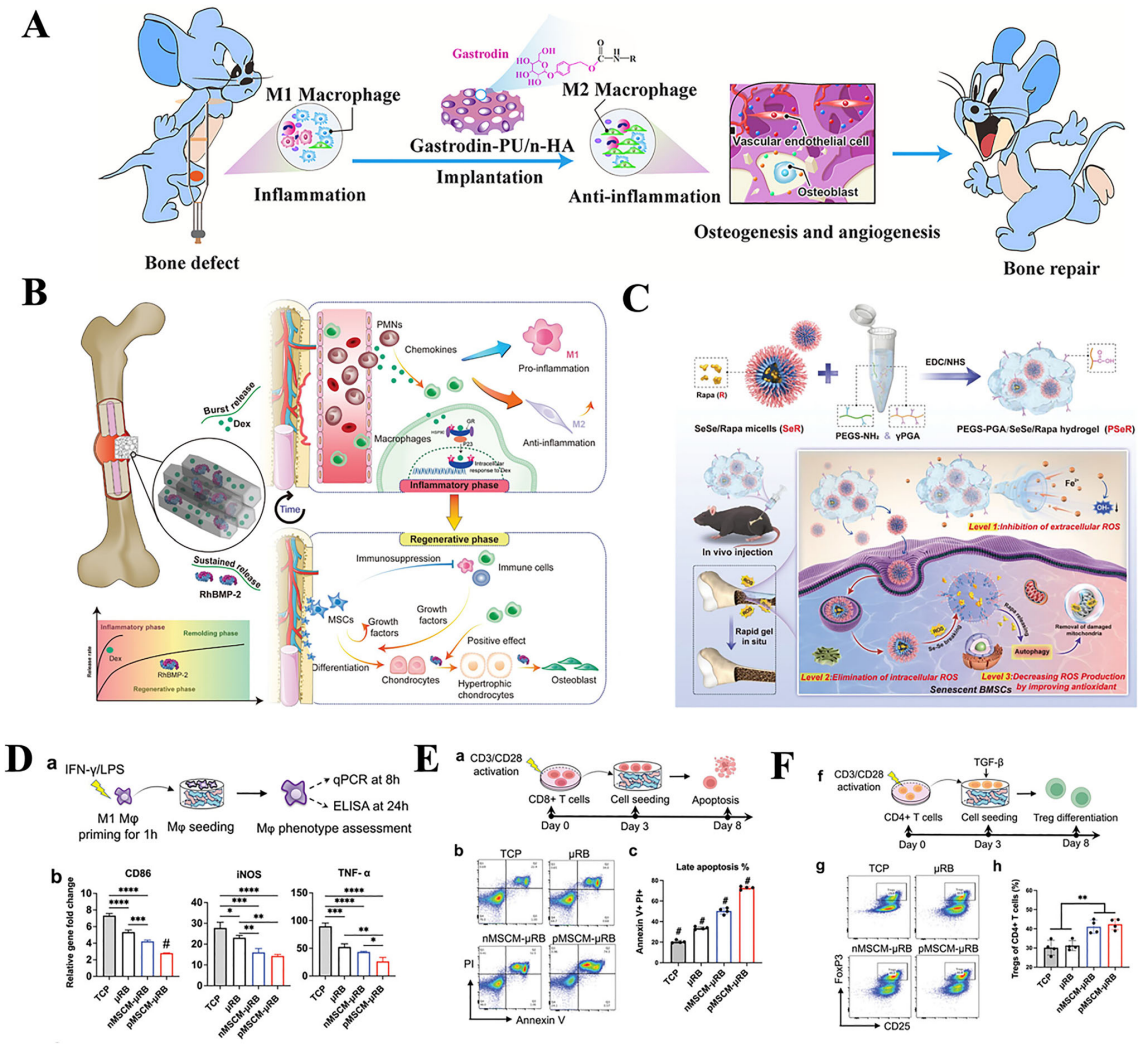
A) Sequential gastrodin release PU/n‐HA composite scaffolds reprogram macrophages for improved osteogenesis and angiogenesis. Reproduced with permission.^[^ ^191^ ^]^ Copyright 2022 The Authors. B) Scheme of designed multiporous tunnels for sequential release of Dex and rhBMP‐2. A burst release of Dex aims to precisely modulate an appropriate inflammatory response in the early stage, and thus promotes the sustained‐release rhBMP‐2‐induced EO bone regeneration. Reproduced with permission.^[^ ^194^ ^]^ Copyright 2021 The Authors. Advanced Science published by Wiley‐VCH GmbH. C) Schematics of a multi‐hierarchy reactive oxygen species (ROS) scavenging system for aged bone regeneration. Reproduced with permission.^[^ ^198^ ^]^ Copyright 2023 The Authors. Advanced Materials published by Wiley‐VCH GmbH. D) a) Schematic of experimental design. Four groups were evaluated, including 2D tissue culture plastic (TCP), uncoated µRB scaffolds, and µRB scaffolds coated with nMSCM or pMSCM. b) Normalized gene expressions of M1 Mφ markers. Data are represented as mean ± S.D. (*n* = 4/group). ^*^ *p* < 0.05, ^**^ *p* < 0.01, ^***^ *p* < 0.001, ^****^ *p* < 0.0001. E) MSCM‐coating rescued MSC osteogenesis and mineral deposition in vitro by inducing CD8+ T cell apoptosis. a–c) Evaluating the effects of MSCM coating on TCD8+ apoptosis using flow cytometry; b) representative flow cytometry plots and c) quantification of apoptotic CD8+ cells. F) MSCM‐coating rescued MSC osteogenesis and mineral deposition in vitro by enhancing Treg differentiation. f–h) Evaluating the effects of MSCM coating on Treg differentiation; g) Representative flow cytometry plots and h) quantification of Tregs (of CD25+ FoxP3+); Data are represented as mean ± S.D. (*n* = 4/group). Scale bars: 200 µm. ^**^ *p* < 0.01, # indicates *p* < 0.001. Reproduced with permission.^[^ ^205^ ^]^ Copyright 2023 Wiley‐VCH GmbH.

Gastrodin, a plant‐derived bioactive molecule, is known for its ability to protect cells from oxidative stress and exert anti‐inflammatory effects, thereby enhancing bone immune regulation. Its multiple hydroxyl functional groups enable effective chemical modification. Researchers have successfully encapsulated aspalathin in a polyurethane/n‐HA matrix for sustained release (**Figure** **6**A). This system promotes macrophage polarization toward the M2 phenotype and significantly increases the levels of anti‐inflammatory factors, optimizing the immune and anti‐inflammatory microenvironments. Cytokines released by M2 macrophages further promote the osteogenic differentiation of MSCs, thereby facilitating vascularization and bone regeneration.^[^ ^192^ ^]^

Additionally, a plant polyphenol called epigallocatechin gallate (EGCG), a major component of tea polyphenols, exhibits strong immunomodulatory activity. Xiao et al. constructed a silica/EGCG nanocoating on the surface of polycaprolactone (PCL) scaffold materials. The nanocoating not only increased the surface roughness of the material, which, as previously discussed, can influence macrophage polarization, but also provided excellent immunomodulatory capabilities owing to the phenolic hydroxyl functional groups in EGCG. The results showed that the scaffold material exhibited strong free radical scavenging ability, intracellular ROS clearance, and overall immunomodulatory effects.^[^ ^193^ ^]^

Dexamethasone (DEX), a glucocorticoid known for its anti‐inflammatory and osteogenic properties, can be delivered in a controlled manner to regulate inflammation and immune responses. Liu et al. developed a bioglass bone filler material loaded with DEX and recombinant human bone morphogenetic protein (rhBMP) to modulate the early inflammatory response. This approach significantly reduced macrophage overinfiltration and promoted the transition from M1 to M2 macrophages. It also enhances the secretion of chemokines and cytokines, such as Arg‐1 and cluster of differentiation206 (CD206)^[^ ^194^ ^]^ (Figure 6B).

Puerarin, an isoflavonoid with potent anti‐inflammatory and antibacterial properties, has been shown to effectively regulate macrophage polarization toward the M2 phenotype. By binding to lipopolysaccharide (LPS), which typically triggers an inflammatory response, puerarin reduces inflammation and has a strong immunomodulatory effect that promotes osteogenesis.^[^ ^195^ ^]^

Curcumin (Cur) inhibits the toll‐like receptor 4 (TLR4)‐mediated signaling pathway, thereby decreasing the ratio of M1/M2 macrophages and contributing to bone immunomodulation. Li et al. prepared a three‐layer bone scaffold material. Magnesium oxide was used as the inner layer, l‐lactide containing curcumin as the interlayer, and mesoporous silica and dehydrated dicalcium phosphate were used as the outer layer. The released curcumin can well regulate the immune microenvironment of bone.^[^ ^196^ ^]^ Additionally, researchers have prepared a reversible degradable nanomicelle based on a diselenide compound capable of effectively loading rapamycin, a drug known for its antioxidative and antiaging effects.^[^ ^197^ ^]^ This nanomicelle activates the mechanistic target of rapamycin (mTOR) signaling pathway to regulate cell behavior. With a high‐sensitivity ROS response to the aging microenvironment, diselenide‐carbonate micelles efficiently remove accumulated ROS within senescent BMSCs, thereby creating a more favorable microenvironment for aging stem cells^[^ ^198^ ^]^ (Figure 6C).

#### 3.3.3 Peptides

Peptides, which are composed of amino acids, serve as bioactive molecules in bone repair biomaterials and play crucial roles in regulating immune responses through cell surface receptors or signaling pathways, thereby fostering a conducive osteogenic microenvironment.^[^ ^199^, ^200^, ^201^ ^]^ Egusa and colleagues synthesized a short peptide composed of seven amino acids, SVVYGLR. This peptide was shown to inhibit the expression of genes associated with bone marrow‐derived macrophages (BMMs) and reduce the number of TRAP‐positive osteoclasts, thereby suppressing osteoclast activation. Additionally, the SVVYGLR peptide inhibited the activation of nuclear factor of activated T cells (NFAT) in RANKL‐stimulated RAW264.7 cells, leading to reduced mRNA expression of calcitonin receptor, cathepsin K, and TRAP (all downstream targets of NFATc1). By regulating immune‐related signaling pathways, such as the RANKL/NFATc1 pathway, the peptide influences the bone regeneration process, modulating the immune system's role in bone remodeling and repair. These combined effects help maintain the balance between osteoblasts and osteoclasts during bone regeneration.^[^ ^202^ ^]^

Furthermore, researchers have explored the sequential release of two peptides from biomaterials to mimic the spatiotemporal regulation of macrophage polarization (M1/M2) during bone regeneration, resulting in a beneficial immune microenvironment. Microspheres containing the LL37 peptide and WP9QY (W9) peptide were encapsulated in a hydrogel and used as a bone defect filler. Initially, the LL37 peptide, known for its antibacterial and proinflammatory properties, recruits immune cells, as well as MSCs, to clear bacteria and pathogens The release of the W9 peptide subsequently exerts potent anti‐inflammatory effects, promoting macrophage polarization toward the M2 phenotype, which significantly enhances bone healing.^[^ ^203^ ^]^

### 3.4 Cell‐Based Biomaterials

#### 3.4.1 Cells

MSCs are critical for regulating bone homeostasis and establishing a favorable immune microenvironment that can activate and enhance the process of bone reconstruction. Consequently, the transplantation of exogenous MSCs is widely used in bone tissue repair. MSCs can influence other cells and the surrounding microenvironment by releasing cytokines or vesicles.^[^ ^35^ ^]^ However, when stem cells are implanted in vivo for bone repair, their viability can decrease, leading to impaired differentiation function and a reduced ability to secrete therapeutic proteins. Additionally, issues such as immune rejection pose risks, limiting the broader application of stem cell implantation. Immune cells, particularly macrophages, play crucial roles in immunomodulation within the context of bone tissue engineering. Sara Nadine and colleagues developed a bioencapsulation system by coculturing monocyte‐derived macrophages, MSCs, and HUVECs to simulate the specific microenvironment during bone regeneration. Macrophages play an anti‐inflammatory and tissue‐repair role through polarization within the microenvironment, particularly toward the M2 phenotype. This polarization not only helps reduce local inflammatory responses but also regulates osteoblast activity through the secretion of anti‐inflammatory cytokines, such as IL‐10 and TGF‐β, thereby promoting osteogenic differentiation. Carefully orchestrating signaling and interactions between cells further enhances this process. This system was designed to foster cell interactions, improving both the immune microenvironment and supporting osteogenic differentiation and angiogenesis.^[^ ^204^ ^]^

#### 3.4.2 Cell Membrane

Innovations in cell membrane coating materials utilize the complex signaling inherent in natural cell membranes to emulate intricate immune cell functions. Su and colleagues investigated a macroporous microstrip scaffold coated with mesenchymal stem cell membrane (MSCM) for repairing critical‐sized cranial defects. The MSCM is rich in immunomodulatory ligands that regulate both innate and adaptive immunity, which are crucial for bone regeneration. After IFN‐γ/LPS‐induced macrophages were inoculated onto the MSCM‐coated microstrip rib (µRB) scaffold, M1 macrophage markers such as TNF‐α and cluster of differentiation 86 (CD86) were downregulated (Figure 6D). Furthermore, this scaffold modulated T‐cell fate; in vitro inoculation with cluster of differentiation 8‐positive T cells (CD8+ T cells) activated by cluster of differentiation 3/cluster of differentiation 28 (CD3/CD28) Dynabeads led to increased apoptosis of these cells, highlighting their inhibitory effect (Figure 6E). Conversely, the cluster of differentiated 4‐positive T cells (CD4+ T cells) activated under similar conditions showed enhanced differentiation into Tregs, bolstering bone regeneration (Figure 6F).^[^ ^205^ ^]^ Therefore, this biomimetic bone scaffold material with cell membrane coating can regulate the immune system by binding various secretory factors on the cell membrane.

#### 3.4.3 Exosomes

In addition to serving as cell membranes, exosomes also serve as crucial communication mediators between cells and are effective bone induction factors.^[^ ^206^, ^207^, ^208^ ^]^ Exosomes derived from MSCs have been shown to induce macrophage polarization toward the M2 phenotype, inhibit harmful immune responses, and promote tissue repair, as confirmed by various studies. Exosomes from HUVECs reportedly increase the angiogenic capacity of endothelial cells. Moreover, exosomes expressing nuclear enriched abundant transcript 1 (NEAT1) significantly increase the infiltration capacity of M2 macrophages while promoting the secretion of anti‐inflammatory cytokines such as IL‐10 and reducing the levels of proinflammatory cytokines such as IL‐1β and IL‐6. Additionally, these exosomes have been shown to effectively promote the migration and differentiation of MSCs, further aiding in tissue regeneration.^[^ ^209^ ^]^

**Figure 7:**
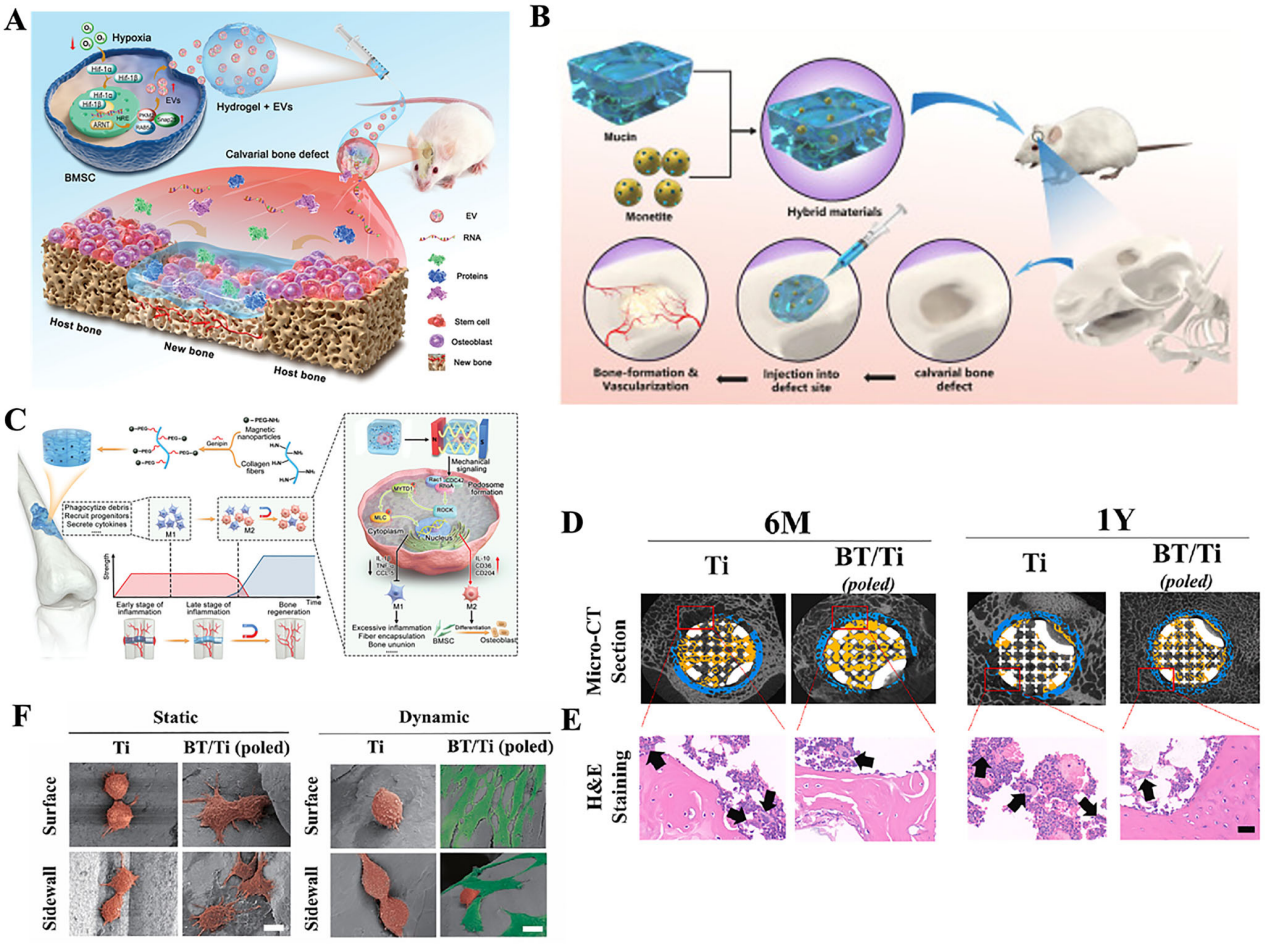
A) Schematic illustration of an injectable hydrogel containing versatile Hypo‐EVs for calvarial defect repair. The hydrogel depot could continuously liberate Hypo‐EVs at the defect site. The cargo in Hypo‐EVs could recruit osteoblasts and BMSCs and promote their proliferation and osteogenic differentiation, thereby accelerating the bone regeneration. Reproduced with permission.^[^ ^302^ ^]^ Copyright 2023 Wiley‐VCH GmbH. B) Early osteoimmunomodulation by mucin hydrogels augments the healing and revascularization of rat critical‐size calvarial bone defects. Reproduced with permission.^[^ ^217^ ^]^ Copyright 2023 The Authors. C) Schematic illustration of magnetized nanocomposite hydrogels for on‐demand immunomodulation via temporally controlled macrophage phenotypic transition in response to a magnetic field. Fabrication processes for the magnetized hydrogels and the scheduled inflammation regulation strategy through a timely programmed macrophage phenotypic switching from M1 to M2 polarization under the manipulation of a magnetic field. M1/M2, M1/M2 polarized macrophage; BMSC, bone mesenchymal stem cell. Reproduced with permission.^[^ ^58^ ^]^ Copyright 2022 The Authors. Small, published by Wiley‐VCH GmbH. D) Micro‐CT section of the osteo‐integration around the artificial bodies, red box indicates the dark zone. E) Representative H&E staining images of FBGCs in bone tissues around artificial vertebral bodies, scale bar = 50 µm. F) Phenotype changes in RAW264.7 macrophages observed by SEM after piezoelectric stimulation under dynamic loading, scale bar = 5 µm. Reproduced with permission.^[^ ^231^ ^]^ Copyright 2022 Elsevier Ltd. All rights reserved.

Exosomes secreted from human adipose‐derived stem cells (hADSC‐Exos) also have potent osteogenic and immunomodulatory abilities,^[^ ^210^, ^211^, ^212^ ^]^ in addition to those secreted by HUVECs and MSCs.^[^ ^213^, ^214^ ^]^ Yue et al. prepared exosome‐functionalized bone repair scaffolds loaded with Mg^2+^ and gallic acid (GA). They cocultured hADSC‐Exos labeled with PKH26 with RAW264.7 cells and reported that hADSC‐Exos were phagocytosed by the cells. They also observed that hADSC‐Exos created a favorable microenvironment for tissue growth, as evidenced by the expression of M1 macrophage‐specific proteins iNOS and cyclooxygenase‐2 (COX‐2) were detected, and promoted the formation of new bone.^[^ ^215^ ^]^ Furthermore, Deng et al. enhanced the production and protein content of extracellular vesicles from BMSCs preconditioned under hypoxic conditions. These vesicles were encapsulated in an injectable bone filler material composed of a polyethylene glycol–polypeptide copolymer, which allowed for sustained release and potentiated osteogenesis via activation of the PI3K/AKT signaling pathway (**Figure** **7**A).^[^ ^216^ ^]^

#### 3.4.4 Mucins

Mucins on the cell surface play a significant role in immunoregulation. For example, intestinal mucinoglycans can promote immune tolerance in dendritic cells by interacting with their galactoglucan‐3 receptor proteins. Additionally, mucin hydrogels have demonstrated minimal complement activation following in vivo implantation, effectively inhibiting macrophage activation and cytokine release. This characteristic positions mucins as promising candidates for bone regeneration biomaterials. Recent advancements include the development of composite hydrogels from bovine submandibular mucin modified with tetrazine (Tz) and norbornene (Nb). When these hydrogels were implanted into rat skulls, researchers observed a significant reduction in iNOS‐positive macrophages, leading to the suppression of inflammatory factor secretion. This reduction in inflammation not only facilitates the resolution of acute inflammatory responses but also enhances bone tissue regeneration^[^ ^217^ ^]^ (Figure 7B).

### 3.5 External Stimuli in Immune Modulation by Biomaterials

Externally responsive biomaterials are designed to adapt their physical or chemical properties in response to external stimuli, thereby influencing the immune microenvironment involved in bone regeneration.^[^ ^218^, ^219^, ^220^ ^]^ These advanced materials utilize external signals to facilitate precise drug delivery or to modify the physicochemical conditions essential for bone regeneration (**Table** **4**). By responding dynamically to these stimuli, these biomaterials can activate immune cells, reduce inflammation, and regulate the activities of osteoblasts and osteoclasts, thereby promoting bone regeneration.^[^ ^221^ ^]^ This responsive behavior not only enhances the effectiveness of treatments but also optimizes the timing and localization of therapeutic interventions, offering a more sophisticated and targeted approach to bone healing^[^ ^222^, ^223^ ^]^ (**Table** **5**).

**Table 4:** Cell‐based biomaterials.

Cell‐based biomaterials	Cell	Materials	Outcome	Refs.
cells	Macrophages, MSCs, and HUVECs	Macrophages, MSCs, and HUVECs to construct a bioencapsulation system	induce macrophage M2‐type polarization and anti‐inflammatory cytokine	[204]
Cell membrane	MSCM	A macroporous microstrip scaffold coated with MSCM	CD8+T cell apoptosis was increased, and T‐reg differentiation was enhanced	[205]
Exosomes	Exosomes of MSCs/HUVEC	alginate/GelMA IPN hydrogels	convert macrophages toward M2, inhibit deleterious immune responses	[209]
	Exosomes secreted from hADSC (hADSC‐Exos)	PLGA/Exo‐Mg‐GA MOF	the expression levels of M1 macrophage specific proteins iNOS and COX‐2 were down‐regulated	[215]
	Hypoxic preconditioned BMSC‐derived extracellular vesicles (Hypo‐EVs)	mPEG‐PA hydrogel	activate the PI3K/AKT pathway	[216]
Mucins	Mucins on the cell surface	Monetite	the number of iNOS‐positive macrophages was decreased and promote the resolution of acute inflammation	[217]

**Abbreviations**: MSCM: MSC membrane; hADSC: human adipose‐derived stem cells.

**Table 5:** External stimuli in immune modulation by biomaterials.

Stimulation	Materials	Outcome	Refs.
Magnetic	Superparamagnetic hydrogel	External magnetic field activates podosome/Rho/ROCK mechanotransduction pathway in macrophages	[58]
Electrical	BaTiO3 coated on the porous Ti scaffolds	Inhibit of MAPK/JNK signaling pathway and promote M2 polarization of macrophages	[231]
	BTO/P(VDF‐TrFE) nanocomposite membrane	Inhibit of PI3K‐AKT signaling pathway and polarize toward M2 macrophages	[232]
Light	Injectable and photocurable hydrogel therapeutic	Inhibit of PI3K‐AKT signaling pathway and polarize toward M2 macrophages	[239]
	DHCP‐PIP hydrogel	Regulate the balance of osteoblasts and osteoclasts	[241]
	RGD‐CO@MPDA‐Ti	Polarize toward M2 macrophages and reduce the secretion of pro‐inflammatory factors	[242]
Thermal	Temperature‐sensitive HPCH hydrogel loaded with porous CS	Polarize toward M2 macrophages	[244]
	MSCs‐HPCH hydrogel	Promote the transformation of macrophages from M1 to M2 to produce anti‐inflammatory and tissue regeneration cytokines	[245]
pH	Bisphosphonate‐magnesium ion nanocomposite hyaluronic acid hydrogel system	Block the osteoclast differentiation induced by RANKL	[247]
	Poly (quaternary ammonium salt‐methacrylic acid copolymer) copolymer	The polarization of M1‐type macrophages was inhibited by down‐regulating TLR4/MyD88 pathway	[248]

**Abbreviations**: BTO/P(VDF‐TrFE): BaTiO3/poly(vinylidene fluoride‐trifluoroethylene); CS: hydroxypropyl chitin (HPCH) chitosan.

#### 3.5.1 Magnetic

Magnetoresponsive bone scaffold materials represent a class of intelligent biomaterials embedded with magnetic nano/microparticles that rapidly respond to external magnetic fields. These materials regulate various cellular processes, including proliferation, migration, differentiation, and signal transduction, by leveraging the unique properties of magnetic fields.^[^ ^224^, ^225^, ^226^ ^]^ Magneto‐responsive bone scaffolds can be categorized into three main types: the first type combines magnetic nanoparticles with biodegradable polymers to create superparamagnetic‐responsive scaffolds with nanofiber structures, often fabricated by electrospinning technology; the second type integrates magnetic nanoparticles with bioceramic materials, forming porous scaffolds through methods such as sol‒gel processing, freeze‐drying, or hot pressing; and the third type involves magnetic alloy materials, such as cobalt‒chromium and nickel‒titanium alloys, which are processed into magnetically responsive scaffolds through techniques such as heat treatment, surface modification, or laser melting.^[^ ^227^ ^]^ Huang et al. developed a superparamagnetic hydrogel and introduced a method to control macrophage polarization toward the M1 or M2 phenotype using an external static magnetic field. They also achieved time‐engineered transformation from M1 to M2 macrophages via adjusting the timing of magnetic field switching. This technique effectively harnesses the role of M1 macrophages in the early stages of tissue regeneration while enhancing the regenerative capacity of M2 macrophages in the later stages. The regulation of macrophage polarization by the magnetized hydrogel is thought to be mediated by magnetic manipulation that activates the podosome/Rho/ROCK mechanotransduction pathway in macrophages, influencing their polarization (Figure 7C).^[^ ^58^ ^]^

#### 3.5.2 Electrical

Electrically responsive bone scaffold materials are a class of smart biomaterials that can either respond to an external electric field or generate their own electrical signals to modulate cell behavior through electrical stimulation.^[^ ^228^, ^229^ ^]^ By incorporating electrical stimulation properties, these materials can directly influence cell behavior, adjust the local microenvironment, and enhance tissue regeneration outcomes. In recent years, electroactive biomaterials have demonstrated significant potential in regenerative medicine. These electrically responsive bone scaffolds can be categorized into three main types: piezoelectric materials, which are made from piezoelectric ceramics or polymers; photovoltaic materials, which are composed of inorganic semiconductors or organic photovoltaic polymers; and electrostatic materials, which are scaffolds with electrostatic energy storage capabilities that are typically created through freeze‒drying or hot pressing methods.^[^ ^230^ ^]^ Wu et al. coated Bi_2_TiO_3_ onto porous titanium alloy scaffolds to prepare a piezoelectric scaffold material. The results revealed that the piezoelectric effect of the BT/Ti scaffold modulated the immune response, promoted osteogenic differentiation, and enhanced the calcification of MSCs, accelerating the repair of cervical vertebral bone defects in sheep (Figure 7D). Furthermore, the BT/Ti scaffold alleviated the inflammatory response and promoted the polarization of macrophages to the M2 phenotype (Figure 7E). The study also revealed that piezoelectric stimulation by the BT/Ti scaffold inhibited the mitogen‐activated protein kinase/c‐Jun N‐terminal kinase (MAPK/JNK) signaling pathway and activated oxidative phosphorylation and ATP synthesis in macrophages.^[^ ^231^ ^]^

In a recent study by Sun et al., a piezoelectric membrane capable of generating controlled electrical signals through ultrasound stimulation was developed to immunomodulate the diabetic bone reconstruction process. By programming these electrical signals, researchers are able to regulate macrophage polarization by modulating the expression and phosphorylation levels of AKT2, thereby aligning the temporal pattern of bone healing and promoting diabetic bone repair.^[^ ^232^ ^]^ Researchers have fabricated a ferroelectric BaTiO_3_/poly(vinylidene fluoride‒trifluoroethylene) (BaTiO_3_/PVDF‒TrFE) nanocomposite membrane to mimic the endogenous electrical microenvironment of natural bone tissue. These findings demonstrated that the membrane inhibited high glucose‐induced M1 macrophage polarization and promoted the osteogenic differentiation of BMSCs. Additionally, the study revealed enhanced effects on bone healing in type 2 diabetic rats. The microelectrical environment facilitates bone immunomodulation by inhibiting the expression of AKT2 and interferon regulatory factor 5 (IRF5) within the PI3K‐AKT signaling pathway, reducing M1‐type macrophage polarization under hyperglycemic conditions, while simultaneously promoting M2‐type macrophage polarization.^[^ ^233^ ^]^

#### 3.5.3 Light

Light‐responsive bone scaffold materials are advanced biomaterials whose physicochemical properties can be altered in response to light (ultraviolet, infrared, or visible light) irradiation.^[^ ^234^, ^235^, ^236^ ^]^ These materials regulate the activities of macrophages and stem cells through light modulation, activating intracellular signaling pathways, regulating immune responses, and promoting the regeneration and reconstruction of bone tissue.^[^ ^237^, ^238^ ^]^

**Figure 8:**
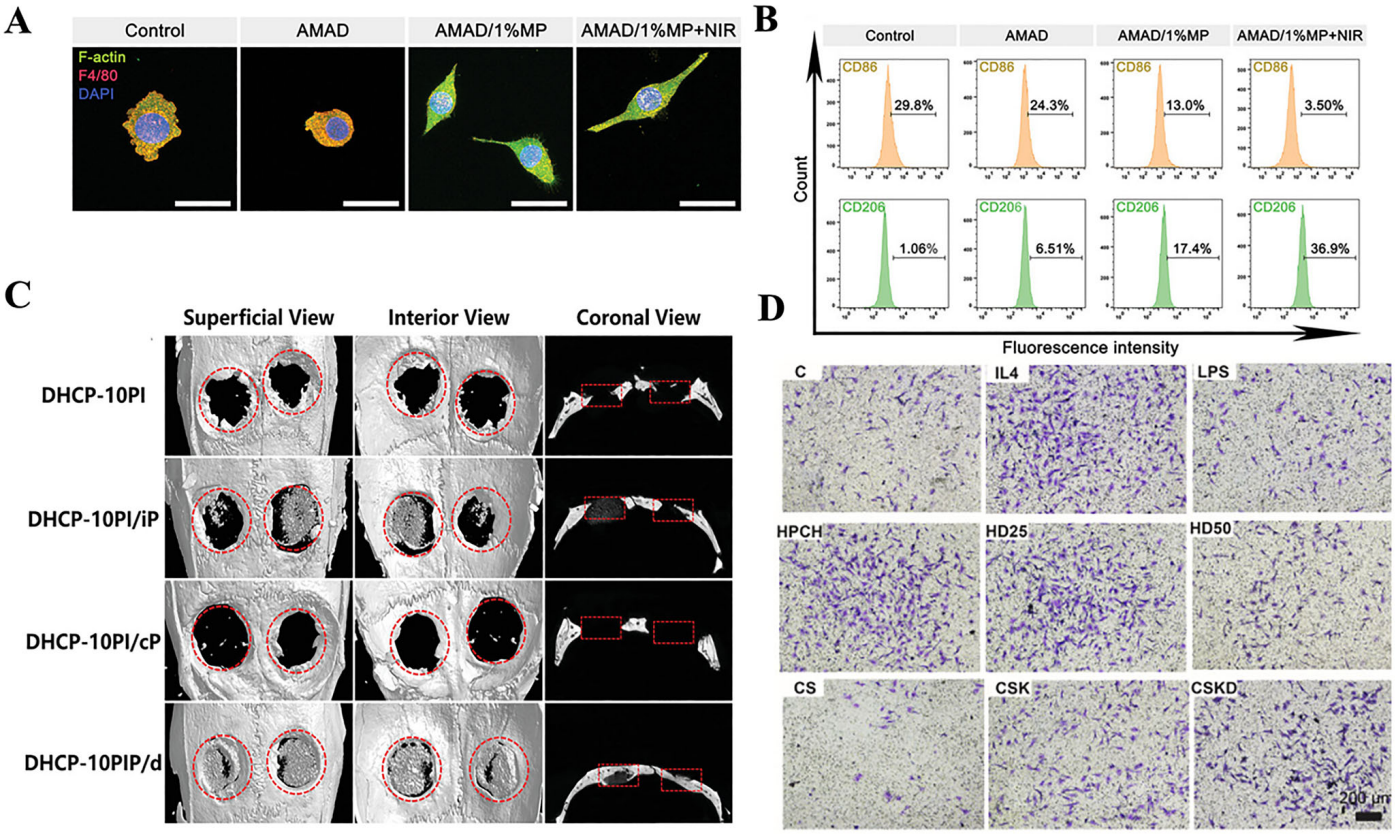
A) Representative cytoskeleton staining images of RAW 264.7 cells after treatment for 4 days. Scale bar: 25 µm. B) Flow cytometry analysis of the M1‐to‐M2 transition in RAW 264.7 cells after treatment for 4 days. Reproduced with permission.^[^ ^239^ ^]^ Copyright 2023 Wiley‐VCH GmbH C) 3D reconstruction images of the defect sites by micro‐CT in osteoporosis rats at week 12. Reproduced with permission.^[^ ^241^ ^]^ Copyright 2021 Wiley‐VCH GmbH. D) Transwell cell migration test of MSCs cultured with macrophage supernatant. Reproduced with permission.^[^ ^244^ ^]^ Copyright 2022 Elsevier Ltd. All rights reserved.

Wu and colleagues developed an injectable, photocurable hydrogel therapeutic platform composed of alginate methacrylate, alginate‐grafted dopamine, and polydopamine‐functionalized nanosheets. Their study demonstrated that the photothermal effect induced by this hydrogel altered the morphology of macrophages cultured in vitro, transforming them from a small, rounded shape to a pseudopod‐like, elongated form (**Figure** **8**A). Anti‐inflammatory cytokine expression was found to increase via flow cytometry, indicating that the organism inhibited the ROS‐induced inflammatory state^[^ ^239^ ^]^ (Figure 8B). These findings suggest that mild photothermal therapy can stimulate the upregulation of heat shock proteins (HSPs), which are typically elevated in response to heat and oxidative stress. This upregulation, mediated through the PI3K/AKT signaling pathway, helps suppress the proinflammatory cascade, contributing to tissue healing and regeneration.

Moreover, near‐infrared (NIR) light can trigger drug release, thereby modulating the immune microenvironment for bone regeneration.^[^ ^240^ ^]^ In a recent study, the authors fabricated an injectable, multifunctional hydrogel composed of in situ‐generated calcium phosphate nanoparticles (ICPNs) coordinated with poly (dimethylaminoethyl methacrylate‐co‐2‐hydroxyethyl methacrylate) and loaded with parathyroid hormone (PTH) for NIR‐stimulated release. In an ovariectomized (OVX) rat model of osteoporosis, the localized photothermal effect activated on‐demand PTH release, which successfully regulated the balance between osteoblasts and osteoclasts. This approach has demonstrated a significant effect on bone regeneration in the rat cranium^[^ ^241^ ^]^ (Figure 8C). Furthermore, Zhang et al. developed peptide‐based implants that covalently grafted carbon monoxide (CO) gas onto arginine‐glycine‐aspartic acid (RGD) peptides. Upon NIR irradiation, CO gas is released on demand, inhibiting the polarization of M1 macrophages and suppressing proinflammatory cytokine secretion. This process promoted the polarization of M1 macrophages toward the M2 phenotype. M2 macrophages, in turn, secrete anti‐inflammatory cytokines such as IL‐10 and TGF‐β, creating a favorable microenvironment for bone repair while mitigating excessive inflammation. This study highlights the significant potential of NIR‐irradiated implants for immunomodulation and osteogenic differentiation.^[^ ^242^ ^]^

#### 3.5.4 Thermal

Thermoresponsive smart immunoregulatory bone regeneration materials represent a sophisticated class of biomaterials whose physical or chemical properties are altered in response to temperature variations.^[^ ^243^ ^]^ These materials are engineered to modulate the cellular states of immune cells or stem cells by releasing bioactive factors, such as cytokines or drugs, thereby enhancing bone regeneration and reconstruction. The ability to deliver growth factors in a spatiotemporal manner, precisely timed to match the phases of bone healing, is essential for effective immunomodulation during bone reconstruction. This advanced functionality ensures that therapeutic interventions are both optimally timed and localized, improving the efficacy and outcomes of bone regeneration strategies. In a study by Ji et al., a thermoresponsive hydroxypropyl chitosan (HPCH) hydrogel loaded with composite porous chitosan (CS) microspheres was developed. The porous structure of the chitosan microspheres promoted cell infiltration and the macrophage colonization, thereby regulating the polarization direction of macrophages (Figure 8D). By promoting the polarization of macrophages toward the M2 phenotype, chitosan microspheres help alleviate local inflammatory responses and facilitate tissue repair and regeneration. The hydrogel matrix not only maintained the positioning of the microspheres but also provided an environment conducive to cell migration and tissue reconstruction, supporting ongoing immunomodulation processes. These experiments demonstrated that this composite hydrogel could effectively modulate the bone regeneration microenvironment at the defect site by regulating macrophage polarization.^[^ ^244^ ^]^ Additionally, researchers have used thermoresponsive HPCH hydrogels as carriers for MSCs and infused these composite hydrogels into 3D‐printed polycaprolactone/nanohydroxyapatite (PCL/nHA) scaffolds. MSCs, known for their low immunogenicity, can promote the transition of macrophages from a classically activated (M1) state to an alternatively activated (M2) state via paracrine mechanisms. This transition facilitates the production of anti‐inflammatory and tissue regenerating cytokines, alleviating inflammatory responses and creating a conducive osteoimmune environment for angiogenesis and bone regeneration.^[^ ^245^ ^]^

#### 3.5.5 pH

pH‐responsive smart materials are a class of functional biomaterials that can sense changes in the pH of the local microenvironment and dynamically adjust their physical and chemical properties (such as swelling, degradation, and drug release). In the field of bone regeneration, such materials achieve spatiotemporal coordination between immune regulation and bone regeneration by targeting the immune microenvironment (such as pH abnormalities under pathological conditions such as inflammation, infection, and metabolic disorders), showing unique advantages. Notably, the pathological bone microenvironment (such as inflammation, infection, metabolic disorders, etc.) is often accompanied by characteristic pH fluctuations: the osteoclast active area forms a local acidic environment (pH≈5.5) due to H+ pump secretion, while the bacterial infection site further reduces the pH due to increased glycolysis. On the basis of this property, researchers have constructed a variety of pH‐responsive biomaterials through molecular design and functional modification and have made a series of breakthroughs in the field of immune regulation‐mediated bone regeneration.

Taking the bone absorption imbalance caused by abnormal osteoclast activation as an example, Ji et al. innovatively developed a self‐assembled bisphosphonate‐magnesium ion nanocomposite hyaluronic acid hydrogel system. It can release bisphosphonates in the pH response, promote the apoptosis of mature osteoclasts, inhibit their excessive differentiation in the local acidic environment where osteoclasts are active, and realize the negative feedback regulation of osteoclast bone absorption via mimicogenesis. The homeostasis of the bone immune microenvironment is regulated to enhance bone regeneration in situ.^[^ ^246^ ^]^ The hydrogel incorporates Embelin, which enables responsive degradation in acidic environments. Thus, the expression of proinflammatory cytokines such as IL‐1β was effectively reduced by inhibiting the NF‐κB pathway, and the osteoclast differentiation induced by RANKL was also blocked. It can effectively suppress inflammation and reverse immune disorders and osteogenesis.^[^ ^247^ ^]^ In terms of immune regulation of infectious bone defects, Zhang et al. developed a pH‐sensitive antibacterial copolymer containing cationic quaternary ammonium salts and carboxyl groups. Under weak acid conditions, charge reversal was achieved, resulting in a positive charge, and the polarization of M1‐type macrophages was inhibited by downregulating the TLR4/MyD88 pathway so that the immune microenvironment at the injured site had an anti‐inflammatory effect.^[^ ^248^ ^]^

These advances signal that smart biomaterials are moving toward a new era of programmed, precise immune regulation.

## 4 Applications of Biomaterials with Immunomodulatory Effects in Bone Regeneration

This article systematically elucidates the tripartite interplay among physicochemical properties, biological functionalities, and immune‐modulatory effects of biomaterials in bone regeneration, particularly focusing on their therapeutic mechanisms under physiological bone remodeling conditions. Notably, while biomaterial‐mediated bone repair has been extensively studied in nonpathological defects, its expanding applications in anatomically diverse and disease‐specific scenarios necessitate deeper mechanistic investigations. Of particular importance, under pathological conditions such as osteoporosis, periodontitis, and osteomyelitis, the immune‐bone crosstalk exhibits distinct regulatory patterns compared to physiological healing, thereby demanding customized biomaterial design principles. In the context of osteoporosis, the dysregulated bone remodeling process is characterized by a skewed osteoclast–osteoblast equilibrium and aberrant immune activation. In this context, ideal biomaterials must execute dual immunomodulatory missions: 1) suppressing excessive osteoclastic resorption through RANKL/OPG axis modulation, and 2) reprogramming proinflammatory macrophages to alleviate TNF‐α/IL‐17‐mediated suppression of osteogenesis. In contrast, periodontitis‐associated bone defects present distinct immunological challenges. The crux lies in biofilm‐induced chronic inflammation that hijacks macrophage polarization toward M1 dominance, with elevated MMP‐9/RANKL levels exacerbating alveolar bone resorption. Mechanistic studies reveal that the immunomodulatory supremacy of pathological bone repair biomaterials stems from their spatiotemporal control over immune cell metabolism. Therefore, the role of biomaterials in the repair of pathological bone defects goes beyond simply providing a physical scaffold; their immune‐modulatory function is equally important. Future research should focus on further exploring the mechanisms through which these biomaterials interact with different immune environments to enable precise repair tailored to various pathological conditions. This will contribute to the development of personalized treatment strategies and offer more effective solutions for the clinical management of bone diseases, ultimately improving patients' quality of life.

### 4.1 Osteoarthritis and Rheumatoid Arthritis

Rheumatoid arthritis (RA), a systemic autoimmune disorder, is characterized by immune homeostasis disruption leading to abnormal autoantibody production (e.g., rheumatoid factor, RF) and subsequent cascading inflammatory responses.^[^ ^249^ ^]^ Notably, this process not only induces synovial tissue destruction and excessive release of proinflammatory cytokines, but also drives osteoclast hyperactivation, resulting in progressive bone erosion and joint deformity that culminate in refractory bone defects.^[^ ^250^, ^251^, ^252^ ^]^ To address this pathological hallmark, emerging biomaterial designs focus on dual regulatory strategies: suppressing excessive immune activation while rebuilding osteoimmune microenvironment homeostasis.

**Figure 9:**
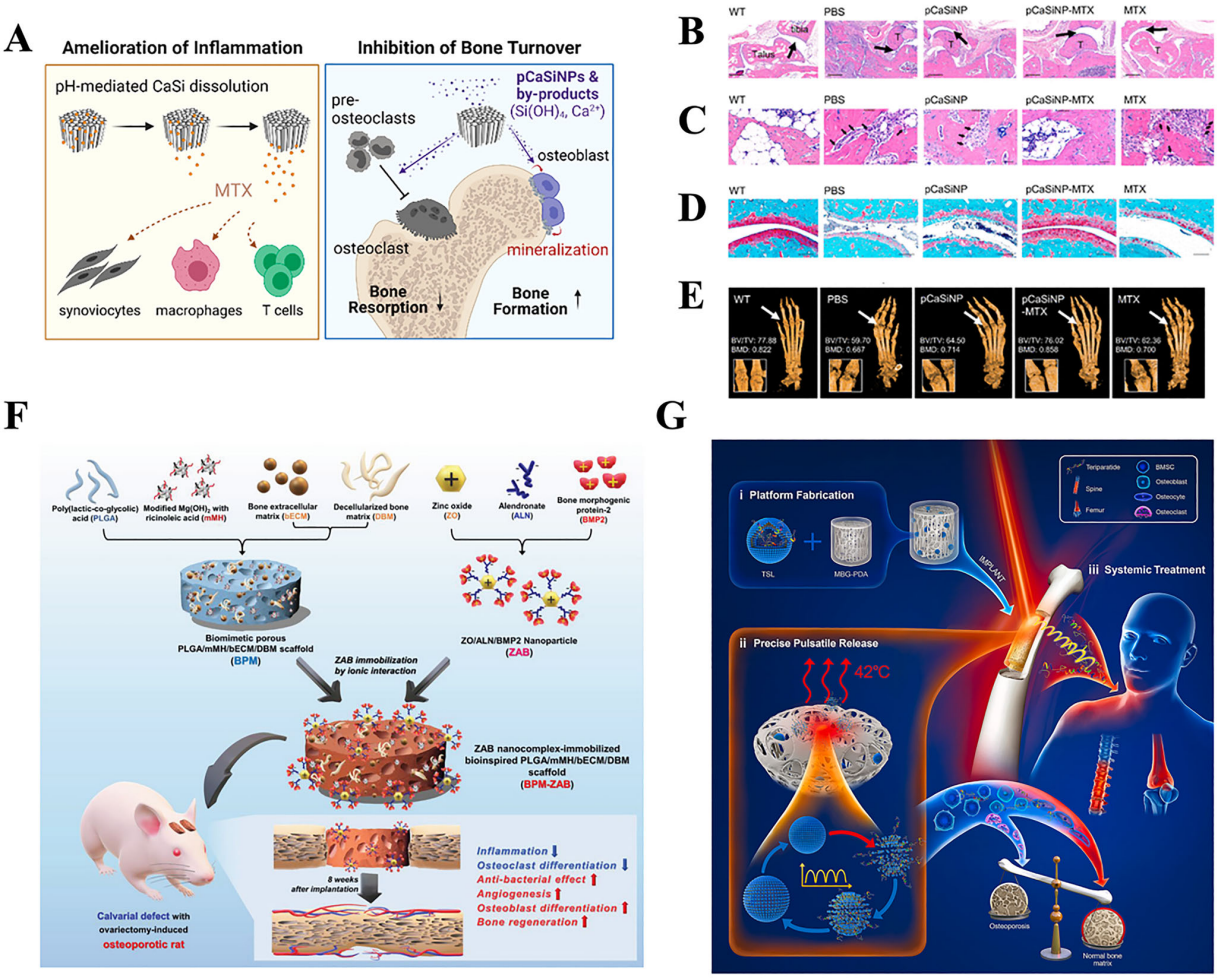
A) Schematic Representation of Mode of Action of the MTX‐Loaded pCaSiNP (pCaSiNP‐MTX) in Rheumatoid Arthritis. B,C) Representative hematoxylin and eosin‐stained images of inflamed joint tissues after treatments showing the gap between talus and tibia (indicated with arrows) (B) and the multinucleated osteoclasts in bone marrow (indicated with arrows) (C). T indicates talus. Scale bars indicate 500 µm in (B) and 50 µm in (C). D) Representative Safranin‐O‐stained images of inflamed joint tissues after treatments. The cells stained in red indicate the chondrocytes. The scale bar indicates 100 µm. E) MicroCT images of the hind paws and joints of treated mice. The insets (left bottom) represent magnified images of toe joints indicated with white arrows. WT, BV/TV, and BMD indicate wild‐type mice, bone volume/tissue volume ratio, and bone mineral density, respectively. Reproduced with permission.^[^ ^253^ ^]^ Copyright 2022, American Chemical Society. F) Schematic illustration of the ZAB‐immobilized bioinspired polymeric scaffold. Reproduced with permission.^[^ ^266^ ^]^ Copyright 2022 The Authors. Advanced Science published by Wiley‐VCH GmbH. G) Schematic illustration of the preparation process of the teriparatide delivery devices and the treatment of local bone defects and systemic osteoporosis. Reproduced with permission.^[^ ^267^ ^]^ Copyright 2022 The Authors.

RA causes not only joint inflammation and pain but also cartilage damage and bone erosion, ultimately leading to bone defects and joint deformities. The immune regulation of RA focuses on reducing the production of autoantibodies, such as rheumatoid factor (RF), by controlling excessive immune activation. Due to the acidic environment characteristic of RA, researchers have developed a method to encapsulate methotrexate (MTX) within a calcium silicate layer. The calcium silicate coating prevents the rapid dissolution of calcium silicate‐porous silicon nanoparticle structures (pCaSiNPs) in the bloodstream (pH 7.4), allowing the drug to be released at the site of inflammation through a pH‐dependent dissolution process (**Figure** **9**A). Calcium silicate preferentially dissolves at acidic pH, and its biodegradation products are protosilicic acid and calcium ions, so it can preferentially release MTX in RA‐associated acidic synovial fluid to inhibit the function of effector T‐cells in the inflamed joints, and it can significantly reduce the immune cells infiltration, inhibit multinucleated osteoclasts formation as well as cartilage damage^[^ ^253^ ^]^ (Figure 9B–E).

Recent studies have indicated an elevated presence of anionic cell‐free DNA (cfDNA) in the serum and joint cavities of RA patients. To address this, researchers synthesized a cationic peptide dendrimer nanogel conjugated with deoxyribonuclease I (DNase I), specifically designed to capture cfDNA. This nanocarrier combines charge‐capturing capabilities with nuclease binding, allowing it to target inflamed joints. Once there, the nanogel can adsorb, degrade, and eliminate cfDNA, effectively alleviating the progression of rheumatoid arthritis. Additionally, cationic polymer nanoparticles (cNPs) exhibit a strong binding affinity for cfDNA and can effectively inhibit the activation of Toll‐like receptor 9 (TLR9) by cfDNA on immune cells, reducing inflammatory responses associated with RA.^[^ ^254^ ^]^

Complementing these approaches, manganese‐based immunomodulatory nanocomposites have emerged as multimodal ROS scavengers. In a groundbreaking study, Chen et al. developed an Mn_3_O_4_/EGCG nanoassembly that synergistically reprograms immune cell functionality in inflamed joints through dual catalytic‐activation mechanisms.^[^ ^255^ ^]^ Although sequential drug release strategies significantly improve bone repair efficiency, synergistic regulation of inflammatory recurrence and abnormal synovial cell death (e.g., iron death) remains a core challenge in RA treatment. In response to this unmet clinical need, an injectable bio‐adhesive hydrogel that targets inflammation and iron death co‐targets significantly alleviates the inflammatory response by down‐regulating the JAK2/STAT3 pathway in macrophages through a dual‐module synergistic mechanism, helps protect chondrocytes from iron autophagy/iron death and restores joint function, and achieves dynamic remodeling of the joint microenvironment.^[^ ^256^ ^]^ A further breakthrough arises from immune–metabolic reprogramming technology. Sagang Koo has developed a nanoparticle that modulates both innate and adaptive immunity, scavenges ROS, and suppresses inflammation in vivo. Nanovesicles secreted by bone marrow stem cells (MSC‐EVs) were used as carriers for cerium dioxide nanoparticles (CeO_2_ NPs) to create a CeO_2_ MSC‐EVs nano mixture. This composite demonstrated high efficiency in scavenging ROS and was shown to significantly reduce oxidative stress and inflammatory responses in joint tissues. Additionally, it modulates the differentiation and function of T cells, inhibiting autoimmune reactions and mitigating tissue destruction and fibrosis in mouse models of rheumatoid arthritis.^[^ ^257^ ^]^

Collectively, these advances unveil the paradigm for next‐generation RA therapeutic biomaterials: Integrating microenvironment‐responsive release, immune signal interception, and metabolic reprogramming to shift from “passive anti‐inflammation” to “active immune reconstruction.”

### 4.2 Osteoporosis

Osteoporosis (OP) is a systemic metabolic bone disease characterized by an imbalance between bone resorption and formation,^[^ ^258^, ^259^ ^]^ resulting in reduced bone mass, compromised bone quality, increased fracture susceptibility, prolonged healing times, and a high rate of nonunion in OP‐related bone defects.^[^ ^260^ ^]^ OP is more prevalent in middle‐aged and elderly women, and conventional drug therapy and hormone therapy can cause many side effects,^[^ ^250^ ^]^ such as bone turnover suppression, jaw necrosis, etc.^[^ ^261^ ^]^ Therefore, biomaterials with immune‐regulatory functions that can promote osteoblast regeneration and inhibit osteoclast reduction have been used to repair bone destruction caused by OP or OP.^[^ ^262^ ^]^ Take bone formation promoters, PTH has been clinically used to treat osteoporosis, but due to its short half‐life, the need for frequent administration, and the risk of hypercalcemia and cardiac toxicity, it can induce more osteoclast activation than osteoblast formation, leading to net bone resorption.^[^ ^263^ ^]^ To address these challenges, controlled release mechanisms are being investigated. In previous research by this group, DEX and Res microspheres were encapsulated in an injectable thermosensitive hydrogel. Given that the osteoporotic microenvironment is characterized by excessive ROS and inflammation, this material was designed to scavenge ROS and modulate macrophage polarization, thereby regulating the immune microenvironment and addressing the underlying pathophysiology of osteoporosis.^[^ ^264^ ^]^

Further breakthroughs came from bone immune metabolism reprogramming strategies. Jun‐Kyu Lee and colleagues prepared ALN, NO,^[^ ^265^ ^]^ BMP‐2 into a novel bioinspired scaffold, which can release NO, achieve triple regulation through a gas signal‐drug synergistic mechanism, and has excellent osteoclast inhibition and osteogenesis ability^[^ ^266^ ^]^ (Figure 9F). NO plays a crucial role in inducing macrophage polarization toward the M2 (anti‐inflammatory) phenotype, which in turn leads to the downregulation of pro‐inflammatory cytokines, such as IL‐1β and IL‐6. This reduction in inflammatory cytokines not only alleviates the inflammatory response during bone regeneration but also promotes angiogenesis and tissue repair, thereby creating a microenvironment that supports bone regeneration. Teriparatide, one of the few globally approved stimulants for bone formation, faces limitations due to side effects associated with high‐dose treatments, such as hypercalcemia and arrhythmia, as well as patient reluctance to undergo daily subcutaneous injections due to pain and injection phobia. Consequently, achieving precise, pulsed release of teriparatide presents significant therapeutic potential. In a groundbreaking approach, teriparatide was encapsulated in thermosensitive liposomes, which were then attached to the surface of bioactive glass coated with polydopamine. Pulsed release of teriparatide was triggered by NIR light, providing a potential solution for osteoporotic bone defects while also demonstrating systemic anti‐osteoporosis efficacy^[^ ^267^ ^]^ (Figure 9G). While immunomodulatory biomaterials have revolutionized bone defect repair, osteoporotic fracture healing remains particularly challenging due to impaired angiogenesis–osteogenesis coupling and excessive osteoclastogenesis. Addressing this unmet need, a zoledronate (ZOL)‐intermixed calcium silicate metal–organic/inorganic hybrid coating on biodegradable Zn‐based intramedullary nails has been engineered to orchestrate angiogenesis‐osteogenesis coupling while suppressing pathological bone resorption.^[^ ^268^ ^]^

Complementing injection systems, the oral targeted nanoplatform has become a game‐changer for OP management by combining patient compliance with precise regulation. A typical example is the multi‐shell bone‐targeting nanoparticle developed by Meng et al., which inhibits osteoclast activity by inhibiting the Sphingosine‐1‐Phosphate Receptor 2 (S1PR2) pathway as well as reducing oxidative stress in BMSCs. Therefore, the treatment of OP changes from passive supplementation to active bone metabolic immune homeostasis reconstruction.^[^ ^269^ ^]^ In addition, a study has shown that oral propolis nanoemulsion can block the vicious cycle of inflammation and bone resorption through the remodeling of intestinal flora and the dual regulation of inflammatory oxidative stress.^[^ ^270^ ^]^

In the field of local bone defect repair, traditional hydrogels are often unable to meet the needs of load‐bearing bone repair due to insufficient mechanical properties (compression modulus <10 kPa), and a single anti‐inflammatory strategy is difficult to synchronously reverse the inflammatory and osteogenic imbalance in the OP microenvironment. In response to this double challenge, the functional reconstruction of osteoporotic bone defects was realized by the mechanical‐immune coupling design of high‐strength gelatin hydrogel scaffolds loaded with drugs. Hypoxia‐inducible factor ‐1α (HIF‐1α) signaling pathway effectively promotes the transformation of inflammatory microenvironment into a microenvironment conducive to bone regeneration, and ultimately enhances osteoporotic bone repair.^[^ ^271^ ^]^

For a long time, the treatment of osteoporosis has faced the dilemma that anti‐bone resorption and promoting bone formation are difficult to cooperate. Traditional drugs such as bisphosphonates (such as alendronate sodium, ALN) can inhibit osteoclast activity, but can not directly promote osteogenesis. However, although sclerotin inhibitors can activate osteogenic differentiation, they have limited inhibition on osteoclastic activity. To address this challenge, Yang et al. developed a carrier‐free two‐drug nanoassembly (Apt/ALN‐Mg) based on aptamer‐driven crystallization technology to achieve a spatiotemporal synergistic effect of osteoclastic inhibition and osteogenic promotion through molecular self‐assembly. The acidic microenvironment of cancellous bone promotes the crystal dissociation, realizes the sequential release of ALN and aptamer, successfully integrates the dual mechanism of anti‐bone resorption and promoting bone formation, and marks the paradigm shift of osteoporosis treatment from “single intervention” to “multi‐target coordination”.^[^ ^272^ ^]^

### 4.3 Alveolar Bone Defect

**Figure 10:**
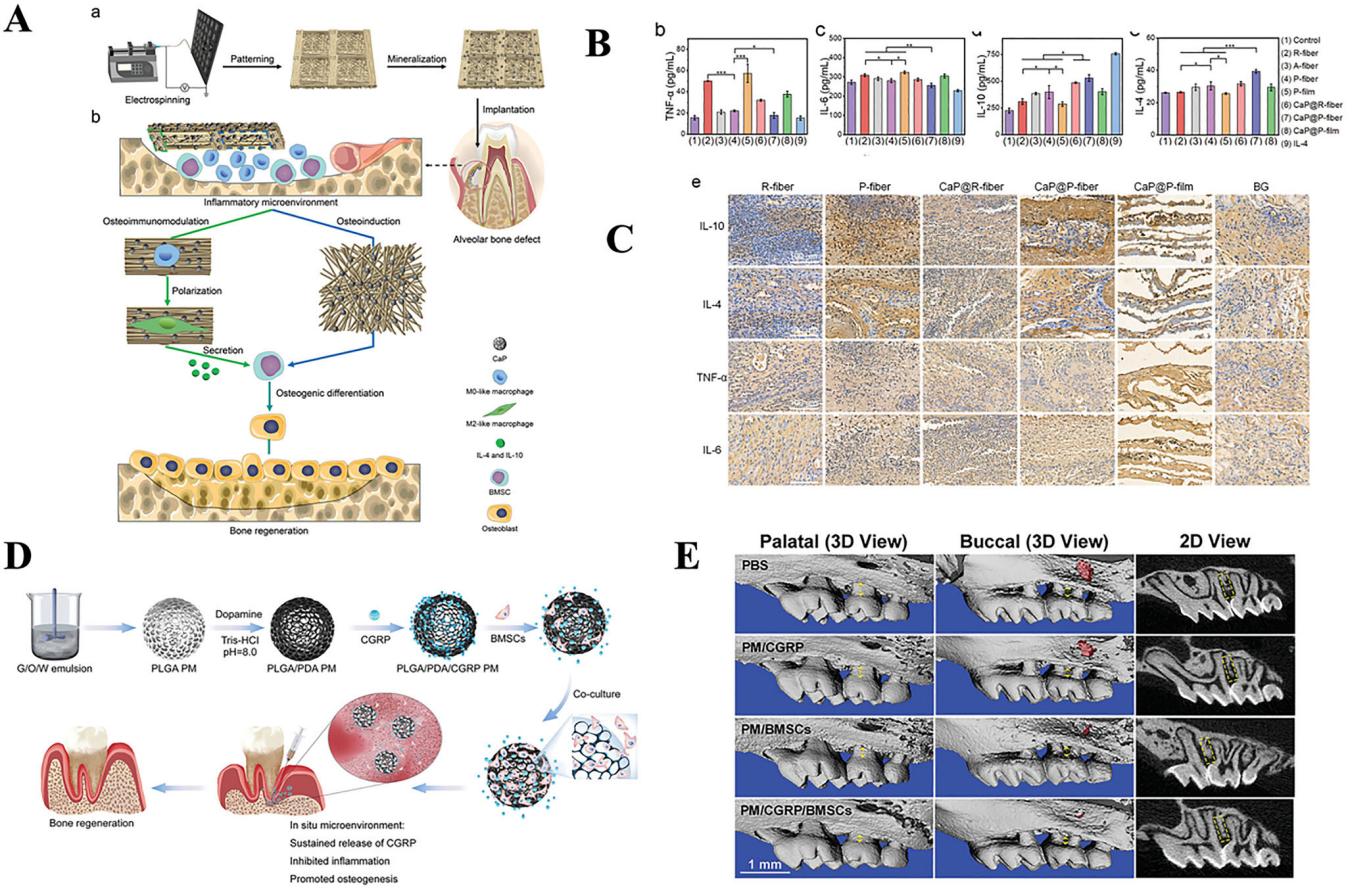
A). Schematic illustration of hierarchical‐structured mineralized nanofiber (HMF) scaffold for enhanced alveolar bone defect repair. B) TNF‐*α*, IL‐6, IL‐10, and IL‐4 by macrophages incubated for 3 days (*n* = 3). (^*^ *p* < 0.05, ^**^ *p* < 0.01, and ^***^ *p* < 0.001). C) Representative immunohistochemistry staining images of IL‐10, IL‐4, TNF‐*α*, and IL‐6. Reproduced with permission.^[^ ^285^ ^]^ Copyright 2021 Wiley‐VCH GmbH D) Schematic illustration of CGRP/BMSC‐loaded PMs preparation for alveolar bone regeneration in periodontitis mouse. E) 3D reconstructions and 2D views of maxillae in the PBS, PM/CGRP, PM/BMSCs, and PM/CGRP/BMSCs groups were generated by micro‐CT. Reproduced with permission.^[^ ^286^ ^]^ Copyright 2023 The Authors. Advanced Healthcare Materials published by Wiley‐VCH GmbH.

**Figure 11:**
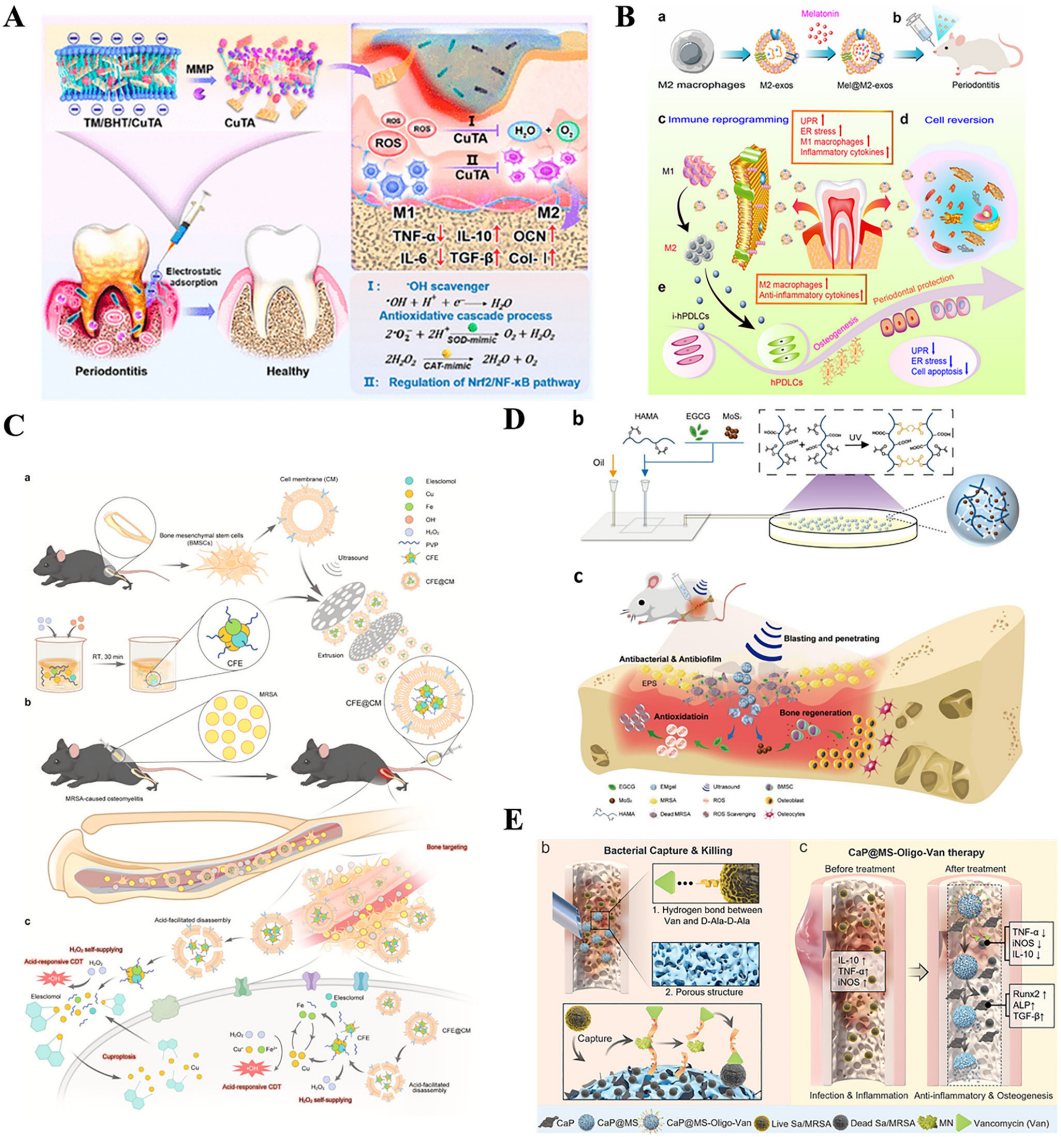
A) Schematic Illustration of the Synthesis of TM/BHT/CuTA Hydrogel and Its Application in Periodontitis. Reproduced with permission.^[^ ^287^ ^]^ Copyright 2021, American Chemical Society. B) Schematic representation of engineered M2 macrophage‐derived exosomes for treating inflammatory bone loss in periodontitis through mediating ER stress and immune reprogramming. Reproduced with permission.^[^ ^289^ ^]^ Copyright 2023 The Authors. C) The fabrication and anti‐osteomyelitis efficacy evaluation of acid‐responsive and nanoenzyme‐loaded artificial nanocells (CFE@CM). Reproduced with permission.^[^ ^294^ ^]^ Copyright 2024 Elsevier Ltd. All rights reserved. D) In situ injection of EMgel into MRSA‐infected chronic osteomyelitis SD rats for killing bacteria and bone regeneration. Reproduced with permission.^[^ ^295^ ^]^ Copyright *2024 Published by Elsevier B.V*. E) The application of nano‐micro CaP@MS‐Oligo‐Van composites for the treatment of osteomyelitis. The composites effectively inhibited the expression of inflammatory cytokines (TNF‐α, iNOS, and IL‐10) and promoted osteogenesis (RUNX2, ALP, and TGF‐β). Reproduced with permission.^[^ ^296^ ^]^ Copyright 2022 The Authors.

Alveolar bone defects refer to incomplete or damaged structures of the alveolar bone, often caused by conditions such as periodontitis or surgical procedures.^[^ ^273^, ^274^ ^]^ Biomaterials play a crucial role in these situations, not only by filling or replacing the damaged alveolar bone but also by serving as carriers for drugs or cells that stimulate bone regeneration.^[^ ^275^ ^]^ Biomaterials provide essential physicochemical stimuli that influence the immune microenvironment of the alveolar bone, regulating bone metabolism balance, enhancing osteoblast activity, and reducing osteoclast activity.^[^ ^276^, ^277^, ^278^, ^279^ ^]^ Additionally, the immune microenvironment at the site of an alveolar bone defect can be modulated through the incorporation of drugs or cells, with factors such as inflammatory responses, immune cell polarization, and cytokine secretion significantly impacting the bone regeneration process and its outcomes.^[^ ^280^, ^281^, ^282^, ^283^, ^284^ ^]^ Yang et al. developed a hierarchically structured mineralized nanofiber with immunomodulatory and osteoblast differentiation‐promoting properties. The scaffold consists of two distinct layers of nanofibers: mineralized oriented nanofibers (anisotropic) and mineralized random nanofibers (isotropic), mimicking the multilevel structure and function of natural bone (**Figure** **10**A). The mineralized oriented nanofiber region exhibits bone immune regulation, effectively reducing the secretion of pro‐inflammatory cytokines (such as TNF‐α and IL‐6) both in vitro and in vivo, while promoting the secretion of anti‐inflammatory cytokines (like IL‐10 and IL‐4) (Figure 10B,C). The mineralized random nanofiber region, on the other hand, initiates the bone induction process, further supporting bone regeneration, and reverses the vicious cycle of inflammatory osteolysis.^[^ ^285^ ^]^ In addition to improving the regulation of the alveolar bone immune microenvironment through surface morphology, biomaterials loaded with peptides can also modulate the immune response at the site of alveolar bone defects. At the molecular regulatory level, periodontitis, primarily caused by anaerobic bacteria in the periodontium, disrupts the immune system, and calcitonin gene‐related peptide (CGRP) plays a key role in modulating the periodontal inflammatory immune microenvironment via the cAMP/PKA signaling pathway. Furthermore, CGRP can regulate bone resorption and bone homeostasis, promoting the reconstruction of the periodontal skeleton. Luo et al. prepared a porous microsphere loaded with CGRP, which protected BMSC from LPS‐induced functional impairment under inflammatory conditions, such as DNA damage, cellular aging, cytokine imbalance, and activation of inflammatory signaling pathways. This approach modulated the immune response and enhanced osteogenesis, contributing to improved bone regeneration at the defect site^[^ ^286^ ^]^ (Figure 10D,E). In addition, due to the accumulation of excessive ROS and MMP at the periodontitis lesion site, Xu et al. prepared a copper tannate‐liganded nano‐enzyme and triglyceride monostearate/2,6‐di‐tert‐butyl‐4‐methylphenol (TM/BHT) hydrogel to self‐assemble to form a TM/BHT/CuTA hydrogel system. The negatively charged hydrogel is retained at the inflammation site through electrostatic adsorption due to the positively charged injury site, exhibiting MMP‐responsive behavior. This system releases nano‐enzymes that regulate macrophage polarization via the Nrf2/NF‐κB pathway, effectively modulating the immune response and promoting healing at the periodontitis lesion^[^ ^287^ ^]^ (**Figure** **11**A). Exosomes have also been demonstrated to be highly effective in the treatment of periodontitis.^[^ ^288^ ^]^ M2 macrophage‐derived exosomes (M2‐exos) exhibit targeted action at sites of bone inflammation and play a regulatory role in the immune system. Cui et al. developed melatonin‐engineered M2‐exos (Mel@M2‐exos), which modulate the secretion of inflammatory cytokines and infiltration of inflammatory cells by reducing excessive endoplasmic reticulum stress and the unfolded protein response. (Figure 11B) This approach significantly reduced alveolar bone loss and restored the osteogenic differentiation capacity of human periodontal ligament cells, offering a promising strategy for bone regeneration in periodontitis.^[^ ^289^ ^]^

In the field of bidirectional immunoosteogenic regulation, a breakthrough has been made in the design of a biomimetic glycopeptide hydrogel system. To inhibit osteoclast maturation by shielding RANKL/RANK interaction and enhancing osteogenic differentiation, thereby synergically regulating and reestablishing bone homeostasis.^[^ ^290^ ^]^

In recent years, the delivery system based on macrophage functional reprogramming has become a hot topic in the treatment of periodontitis. Inspired by the migration ability of natural immune cells, the macrophage penetrating nanoparticle achieves precise intervention of deep lesions in periodontal pocket through bidirectional regulation of immune metabolism.^[^ ^291^ ^]^ In addition, the combined intervention to target the vicious cycle of microbia‐immune imbalance in periodontitis marks the transition of alveolar bone regeneration from a single antibacterial/anti‐inflammatory to a multidimensional microbial‐immune‐mechanical regulation paradigm. Chen et al. prepared a cationic hydrogel to capture anionic microbiome‐associated patterns such as Lipopolysaccharide (LPS) and cfDNA, and reduce the expression of Toll like receptor 4 (TLR4) and Toll like receptor 9 (TLR9) pathways to inhibit the inflammatory response induced by these molecules.^[^ ^292^ ^]^

### 4.4 Osteomyelitis

Osteomyelitis is a bone infection caused by bacteria, fungi, or other pathogenic microorganisms. This condition can result in damage to bone structure and loss of function, often accompanied by abscess formation, osteonecrosis, and the destruction of bone tissue.^[^ ^293^ ^]^ Addressing the challenge of drug‐resistant bacteria biofilms, Li et al. prepared a self‐supplied Fenton catalyst composed of BMSC‐coated nanoparticles Cu–Fe–elesclomol peroxide (CFE), which decomposes at the site of osteomyelitis infection under slightly acidic conditions, and the released Cu^2+^ and Fe^3+^ interfere with copper metabolism. Redox stress and other related biological processes^[^ ^294^ ^]^ (Figure 11C). In addition to the therapeutic effects of biocatalysis on osteomyelitis, ultrasound can also serve as a physical stimulus to enhance the release of bioactive drugs across biological barriers and into the biofilm. Guo et al. developed a multifunctional hydrogel that encapsulates a natural polyphenol EGCG and incorporates MoS_2_ to improve acoustic responsiveness. This hydrogel destroys the biofilm matrix and exhibits superior antioxidant capacity. It can reduce the number of inflammatory cells, reduce TNF‐α levels, increase IL‐10 expression, and regulate the microenvironment at the site of injury. In addition, according to the Agra plate images of different bacteria, ultrasound and microspheres have a strong, broad‐spectrum antibacterial effect, reducing the inflammatory response at the site of bone injury^[^ ^295^ ^]^ (Figure 11D). Lu et al. developed an injectable hydrogel capable of intelligently releasing vancomycin (Van) and calcium phosphate (CaP), which promotes the polarization of M2‐type macrophages. This macrophage phenotype transformation helps suppress acute inflammatory responses and reduces destructive inflammation in osteomyelitis. Furthermore, the hydrogel enhances the regenerative environment at the bone defect site by regulating immune cell activity, leading to the release of anti‐inflammatory cytokines and pro‐repair cytokines^[^ ^296^ ^]^ (Figure 11E).

The difficulties in the treatment of osteomyelitis involve the imbalance of bone metabolism induced by bacteria, and the continuous activation of the immune system leads to a large number of immune cells to infiltrate and then generate pro‐inflammatory factors to induce osteoblast apoptosis. For this purpose, Han et al. constructed a microenvironment‐enhanced supramolecular hydrogel composed of Ce ion and Aln. By promoting calcium inflow, up‐regulating calcium signaling pathway and TGF‐β signaling pathway, and promoting Bmp2/Smad5 path‐related protein expression, thus effectively promoting osteogenic differentiation and reversing the multiple dilemmas of osteomyelitis.^[^ ^297^ ^]^ In the field of treatment of antibiotic‐resistant osteomyelitis, single antibiotic therapy often fails due to biofilm barrier and immunosuppressive microenvironment. Based on this, the mannose‐modified zinc ion‐vancomycin co‐loaded nanoparticles developed by Lv et al. actively target infected macrophages to reduce the level of inflammatory factors and increase bone mass. It indicates that the treatment of drug‐resistant bacteria osteomyelitis has changed from “passive sterilization” to “synergistic regulation of metabolism and immunity.”^[^ ^298^ ^]^

In order to solve the core problems of immune escape and immune memory loss in bacterial osteomyelitis, the in situ vaccine strategy based on biomimetic nanomedical medicine has opened up a new treatment path. Inspired by pathogen‐host interactions, Lin et al. developed a hybrid nanovesicle that relies on bacteria‐associated antigens and can further provide a durable bacteria‐specific immune memory response to prevent infection recurrence. This triggers the maturation of antigen‐presenting cells to activate cellular and humoral adaptive immunity against bacterial infection.^[^ ^299^ ^]^

In conclusion, the emerging strategies for the treatment of osteomyelitis, such as biocatalytic therapies, ultrasound‐enhanced drug delivery, and intelligent hydrogels, highlight the promising potential of advanced biomaterials in combating this debilitating condition. These innovative approaches not only address the microbial infection directly but also actively modulate the immune response and promote tissue regeneration. These advances not only subvert the traditional concept of “infection control first” but also provide a systematic solution from “short‐term sterilization” to “long‐term immune defense‐functional regeneration” for osteomyelitis treatment through the cross‐fusion of material, chemical biology, and immune engineering. It also provides a new idea for the treatment of other pathologically complicated bone defects.

## 5 Summary and Future Outlook

This review provides an overview of recent advancements in the rational design of bone repair materials that can modulate the immune microenvironment to control both the rate and quality of bone healing. The bone microenvironment is essential for maintaining skeletal function and homeostasis, and it significantly influences the process and outcomes of bone regeneration. Therefore, the success of biomaterials in bone healing depends on their high degree of compatibility with the bone microenvironment, as well as their ability to modulate the bone microenvironment. In recent years, progress in immunology and materials science has deepened our understanding of immune cells, cytokines, and cytokine‐mediated signaling pathways, as well as their interactions with bone graft materials. This evolving knowledge has led to a paradigm shift in biomaterial design—from traditional “immune‐friendly” materials, which aim to minimize adverse immune responses such as rejection, inflammation, and fibrosis, to “immune reprogramming” biomaterials. These innovative materials actively regulate specific immune cells and cytokines, thereby promoting an anti‐inflammatory, pro‐angiogenic, and pro‐osteogenic immune microenvironment, ultimately enhancing bone regeneration.^[^ ^300^ ^]^

The strategy of immune modulation by biomaterials in bone regeneration represents a novel and promising research frontier. A comprehensive analysis of existing studies reveals that immune modulation by biomaterials can be categorized into three distinct approaches: physical modification, chemical modification, and functionalization with biomolecules. Physical modifications, such as altering the material's hardness, morphology, charge, pore size, and porosity, can significantly influence the interactions between immune cells and bone cells, including osteoblasts and osteoclasts. Chemical modifications, on the other hand, involve the modification of functional groups on biomaterials to enhance these interactions. Furthermore, the functionalization of biomaterial surfaces with biomolecules—whether through surface decoration or molecule delivery—plays a crucial role in immune modulation, enabling more precise control over the immune microenvironment to promote bone regeneration.

This review also highlights the dynamic capabilities of stimuli‐responsive biomaterials, which can adjust the immune microenvironment in response to local tissue changes. It further emphasizes the importance of tailoring immune‐modulatory biomaterials to specific diseases and skeletal sites, as this can significantly influence their therapeutic effectiveness. For example, in the case of critical‐sized bone defects, the early stimulation of M1 macrophages is essential for clearing necrotic tissue and pathogens. Subsequently, promoting the transition of M2 macrophages is crucial for managing acute inflammation and supporting vascular regeneration and osteoblast differentiation. In contrast, for osteolytic bone loss, such as that seen in osteoporosis or rheumatoid arthritis, the focus shifts toward regulating bone metabolic homeostasis to address the imbalance between osteoblast and osteoclast activity.

Despite substantial progress in the development of immune‐modulatory biomaterials for bone healing, several challenges remain. Given the long‐term implantation of these materials, their biocompatibility must be rigorously assessed to prevent adverse immune reactions and complications. Moreover, the long‐term immune response and potential side effects of these biomaterials have been insufficiently explored, underscoring the need for extended follow‐up and monitoring. Additionally, the precise and controllable delivery of these materials presents a significant hurdle. Current methods for delivering bioactive molecules or cells lack the necessary precision, failing to regulate delivery timing, spatial targeting, and responses to local tissue conditions or physiological signals. Thus, developing more advanced, intelligent delivery systems that can dynamically regulate dosage, timing, effectiveness, and specificity—according to the different stages of bone regeneration—is essential to minimize side effects and toxicity.

**Scheme 1:**
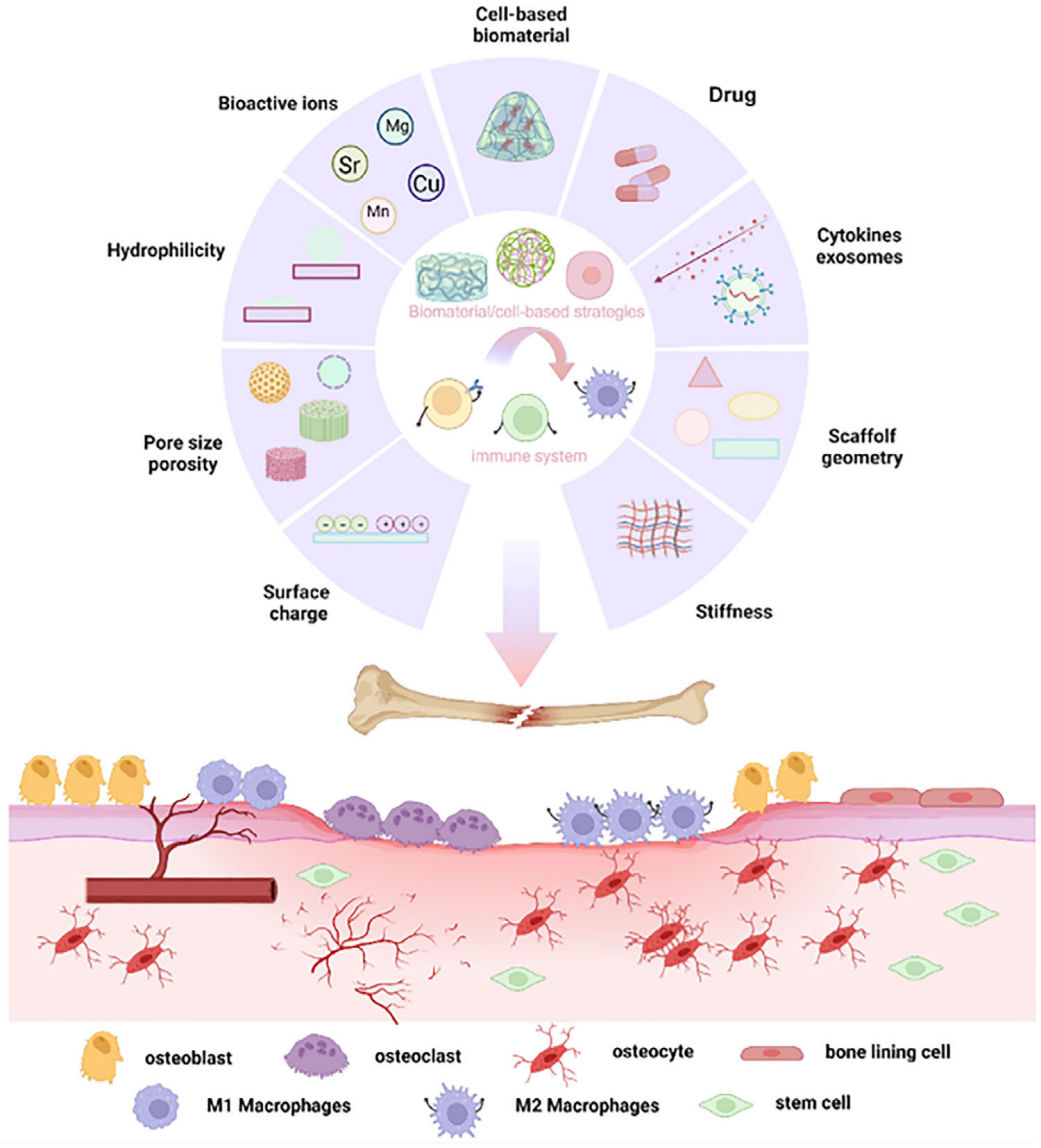
Application of biomaterials in immunomodulation during bone regeneration.

Furthermore, real‐time monitoring of the internal microenvironment at the injury site is critical for assessing the impact of immune‐modulatory biomaterials on bone healing. Currently, most studies rely on animal sacrifice to perform endpoint analyses, such as evaluating immune cell infiltration in local tissues. This approach also poses challenges in differentiating between immune cell types and assessing their activity and status. To overcome these limitations, the development of noninvasive cellular imaging techniques for tracking immune cells within the bone niche is imperative. Technologies such as three‐photon microscopy, combined with advances in transgenic reporter mice and labeling probes, offer promising approaches for the real‐time, noninvasive monitoring of immune cells within the immune microenvironment. These innovations are crucial not only for enhancing our understanding of how biomaterials interact with the immune system during bone regeneration but also for advancing the broader field of regenerative medicine (**Scheme** **1**).

## Conflict of Interest

The authors declare no conflict of interest.

## Author Contributions

J.L., Y.Q. contributed equally to this work. J.L. wrote the original draft, visualized the study, developed the methodology, and performed the investigation. Y.Q. visualized the study, performed validation and investigation, and acquired funds. B.C. acquired funds and resources. T.W. acquired funds and resources. M.P. wrote, reviewed, and edited the final draft and performed validation. D.M. wrote, reviewed, and edited the final draft and performed validation. L.L. wrote, reviewed, and edited the final draft and performed validation. Y.M. visualized the study, developed the methodology, and performed investigation. Y.Y. visualized the study, developed the methodology, and performed investigation. M.W. wrote, reviewed, and edited the final draft and performed validation. X.H. wrote, reviewed, and edited the final draft and performed validation. Z.Q. wrote, reviewed, and edited the final draft, performed supervision and conceptualization, and acquired resources and funds.

## References

[1] X. Gui , B. Zhang , Z. Su , Z. Zhou , Z. Dong , P. Feng , C. Fan , M. Liu , Q. Kong , C. Zhou , Y. Fan , X. Zhang , MedComm – Biomater. Appl. 2023, 2, 41.

[2] S. Chen , Y. Yu , S. Xie , D. Liang , W. Shi , S. Chen , G. Li , W. Tang , C. Liu , Q. He , Nat. Commun. 2023, 14, 7783. 38012166 10.1038/s41467-023-43618-z PMC10682449

[3] Y. Wang , H. Zhang , Y. Hu , Y. Jing , Z. Geng , J. Su , Adv. Funct. Mater. 2022, 32, 2208639.

[4] C. Xu , Z. Liu , X. Chen , Y. Gao , W. Wang , X. Zhuang , H. Zhang , X. Dong , Chin. Chem. Lett. 2024, 35, 108830.

[5] R. J. Miron , M. Bohner , Y. Zhang , D. D. Bosshardt , Periodontol 2000 2024, 94, 9. 37658591 10.1111/prd.12519

[6] H. Takayanagi , Nat. Rev. Rheumatol. 2009, 5, 667. 19884898 10.1038/nrrheum.2009.217

[7] C. Zheng , J. Chen , S. Liu , Y. Jin , Int. J. Oral Sci. 2019, 11, 23. 31423011 10.1038/s41368-019-0060-3 PMC6802669

[8] H. Wu , X. Wei , Y. Liu , H. Dong , Z. Tang , N. Wang , S. Bao , Z. Wu , L. Shi , X. Zheng , X. Li , Z. Guo , Bioact. Mater. 2023, 21, 595. 36685731 10.1016/j.bioactmat.2022.07.032 PMC9832114

[9] M. Qiu , N. Tulufu , G. Tang , W. Ye , J. Qi , L. Deng , C. Li , Adv. Sci. 2024, 11, 2304824. 10.1002/advs.202304824 PMC10767454 37953457

[10] Y. Xiong , B. B. Mi , Z. Lin , Y. Q. Hu , L. Yu , K. K. Zha , A. C. Panayi , T. Yu , L. Chen , Z. P. Liu , A. Patel , Q. Feng , S. H. Zhou , G. H. Liu , Mil. Med. Res. 2022, 9, 65. 36401295 10.1186/s40779-022-00426-8 PMC9675067

[11] K. E. Martin , A. J. Garcia , Acta Biomater. 2021, 133, 4. 33775905 10.1016/j.actbio.2021.03.038 PMC8464623

[12] G. L. Koons , M. Diba , A. G. Mikos , Nat. Rev. Mater. 2020, 5, 584.

[13] B. J. Zhang , M. Zhang , Y. H. Sun , M. M. Li , F. Han , C. T. Wu , Progr. Nat. Sci. Mater. Int. 2021, 31, 883.

[14] C. Xie , J. Ye , R. Liang , X. Yao , X. Wu , Y. Koh , W. Wei , X. Zhang , H. Ouyang , Adv. Healthcare Mater. 2021, 10, 2100408. 10.1002/adhm.202100408 33949147

[15] Y. Liu , D. Luo , T. Wang , Small 2016, 12, 4611. 27322951 10.1002/smll.201600626

[16] T. Wang , J. Bai , M. Lu , C. Huang , D. Geng , G. Chen , L. Wang , J. Qi , W. Cui , L. Deng , Nat. Commun. 2022, 13, 160. 35013289 10.1038/s41467-021-27816-1 PMC8748715

[17] H. Xue , Z. Zhang , Z. Lin , J. Su , A. C. Panayi , Y. Xiong , L. Hu , Y. Hu , L. Chen , C. Yan , X. Xie , Y. Shi , W. Zhou , B. Mi , G. Liu , Bioact. Mater. 2022, 18, 552. 35845319 10.1016/j.bioactmat.2022.05.023 PMC9256949

[18] N. Su , C. Villicana , F. Yang , Biomaterials 2022, 286, 121604. 35667249 10.1016/j.biomaterials.2022.121604 PMC9881498

[19] R. Toita , J. H. Kang , A. Tsuchiya , Acta Biomater. 2022, 154, 583. 36273800 10.1016/j.actbio.2022.10.024

[20] S. Tang , Z. Dong , X. Ke , J. Luo , J. Li , Int. J. Oral Sci. 2021, 13, 42. 34876550 10.1038/s41368-021-00147-z PMC8651686

[21] T. A. Einhorn , L. C. Gerstenfeld , Nat. Rev. Rheumatol. 2015, 11, 45. 25266456 10.1038/nrrheum.2014.164 PMC4464690

[22] Z. Lin , Y. Xiong , W. Meng , Y. Hu , L. Chen , L. Chen , H. Xue , A. C. Panayi , W. Zhou , Y. Sun , F. Cao , G. Liu , L. Hu , C. Yan , X. Xie , C. Lin , K. Cai , Q. Feng , B. Mi , G. Liu , Bioact. Mater. 2022, 13, 300. 35224310 10.1016/j.bioactmat.2021.10.042 PMC8844834

[23] G. N. Duda , S. Geissler , S. Checa , S. Tsitsilonis , A. Petersen , K. Schmidt‐Bleek , Nat. Rev. Rheumatol. 2023, 19, 78. 36624263 10.1038/s41584-022-00887-0

[24] S. Shirazi , S. Ravindran , L. F. Cooper , Biomaterials 2022, 291, 121903. 36410109 10.1016/j.biomaterials.2022.121903 PMC10148651

[25] B. Tan , Q. Tang , Y. Zhong , Y. Wei , L. He , Y. Wu , J. Wu , J. Liao , Int. J. Oral Sci. 2021, 13, 9. 33727527 10.1038/s41368-021-00113-9 PMC7966790

[26] N. Yang , Y. Liu , Int J Med Sci 2021, 18, 3697. 34790042 10.7150/ijms.61080 PMC8579305

[27] K. Jiang , C. Luo , Y. M. Li , K. Wang , S. Huang , X. H. You , Y. Liu , E. Luo , J. Z. Xu , L. Zhang , Z. M. Li , Int. J. Biol. Macromol. 2024, 281, 136375. 39383912 10.1016/j.ijbiomac.2024.136375

[28] R. Dimitriou , E. Jones , D. McGonagle , P. V. Giannoudis , BMC Med. 2011, 9, 66. 21627784 10.1186/1741-7015-9-66 PMC3123714

[29] B. Li , P. Wang , J. Jiao , H. Wei , W. Xu , P. Zhou , Front. Immunol. 2022, 13, 824117. 35386705 10.3389/fimmu.2022.824117 PMC8977491

[30] Z. Lin , D. Shen , W. Zhou , Y. Zheng , T. Kong , X. Liu , S. Wu , P. K. Chu , Y. Zhao , J. Wu , K. M. C. Cheung , K. W. K. Yeung , Bioact. Mater. 2021, 6, 2315. 33553818 10.1016/j.bioactmat.2021.01.018 PMC7840811

[31] M. C. Walsh , N. Takegahara , H. Kim , Y. Choi , Nat. Rev. Rheumatol. 2018, 14, 146. 29323344 10.1038/nrrheum.2017.213 PMC5821527

[32] P. N. Soucacos , E. O. Johnson , G. Babis , Injury 2008, 39, S1. 10.1016/S0020-1383(08)70009-3 18804569

[33] W. Qiao , H. Xie , J. Fang , J. Shen , W. Li , D. Shen , J. Wu , S. Wu , X. Liu , Y. Zheng , K. M. C. Cheung , K. W. K. Yeung , Biomaterials 2021, 276, 121038. 34339925 10.1016/j.biomaterials.2021.121038

[34] A. Terashima , H. Takayanagi , Semin. Immunopathol. 2019, 41, 619. 31552472 10.1007/s00281-019-00755-2

[35] B. D. Sui , C. H. Hu , A. Q. Liu , C. X. Zheng , K. Xuan , Y. Jin , Biomaterials 2019, 196, 18. 29122279 10.1016/j.biomaterials.2017.10.046

[36] A. Salhotra , H. N. Shah , B. Levi , M. T. Longaker , Nat. Rev. Mol. Cell Biol. 2020, 21, 696. 32901139 10.1038/s41580-020-00279-w PMC7699981

[37] J. Wen , D. Cai , W. Gao , R. He , Y. Li , Y. Zhou , T. Klein , L. Xiao , Y. Xiao , Nanomaterials 2023, 13, 692. 36839060 10.3390/nano13040692 PMC9962115

[38] S. Vermeulen , Z. Tahmasebi Birgani , P. Habibovic , Biomaterials 2022, 283, 121431. 35231787 10.1016/j.biomaterials.2022.121431

[39] M. Majidinia , A. Sadeghpour , B. Yousefi , J. Cell. Physiol. 2018, 233, 2937. 28590066 10.1002/jcp.26042

[40] Y. Li , Z. Xu , J. Wang , X. Pei , J. Chen , Q. Wan , Int. J. Biol. Macromol. 2023, 230, 123246. 36649862 10.1016/j.ijbiomac.2023.123246

[41] N. van Gastel , G. Carmeliet , Nat. Metab. 2021, 3, 11. 33398192 10.1038/s42255-020-00321-3

[42] F. Wang , H. Yao , X. Wu , Y. Tang , Y. Bai , H. Chong , H. Pang , Chin. Chem. Lett. 2024, 35, 108821.

[43] L. Chen , Z. C. Yao , S. Q. Zhang , K. H. Tang , Q. M. Yang , Y. Z. Wang , B. H. Li , Y. J. Nie , X. B. Tian , L. Sun , Chin. Chem. Lett. 2023, 34, 107925.

[44] E. Kolaczkowska , P. Kubes , Nat. Rev. Immunol. 2013, 13, 159. 23435331 10.1038/nri3399

[45] J. E. Won , Y. S. Lee , J. H. Park , J. H. Lee , Y. S. Shin , C. H. Kim , J. C. Knowles , H. W. Kim , Biomaterials 2020, 227, 119548. 31670033 10.1016/j.biomaterials.2019.119548

[46] B. Cai , D. Lin , Y. Li , L. Wang , J. Xie , T. Dai , F. Liu , M. Tang , L. Tian , Y. Yuan , L. Kong , S. G. F. Shen , Adv. Sci. 2021, 8, 2100584. 10.1002/advs.202100584 PMC8498914 34382372

[47] R. Sridharan , A. R. Cameron , D. J. Kelly , C. J. Kearney , F. J. O'Brien , Mater. Today 2015, 18, 313.

[48] B. N. Brown , B. D. Ratner , S. B. Goodman , S. Amar , S. F. Badylak , Biomaterials 2012, 33, 3792. 22386919 10.1016/j.biomaterials.2012.02.034 PMC3727238

[49] D. Li , Z. Yang , X. Zhao , Y. Luo , W. Zhou , J. Xu , Z. Hou , P. Kang , M. Tian , Chem. Eng. J. 2022, 435, 134991.

[50] H. C. Bygd , K. D. Forsmark , K. M. Bratlie , Biomaterials 2015, 56, 187. 25934291 10.1016/j.biomaterials.2015.03.042

[51] D. R. Schmidt , W. J. Kao , Biomaterials 2007, 28, 371. 16978691 10.1016/j.biomaterials.2006.08.041

[52] M. M. Alvarez , J. C. Liu , G. Trujillo‐de Santiago , B. H. Cha , A. Vishwakarma , A. M. Ghaemmaghami , A. Khademhosseini , J. Control. Release 2016, 240, 349. 26778695 10.1016/j.jconrel.2016.01.026 PMC4945478

[53] J. Li , X. Jiang , H. Li , M. Gelinsky , Z. Gu , Adv. Mater. 2021, 33, 2004172. 10.1002/adma.202004172 PMC9245340 33565154

[54] B. M Delavary , W. M. van der Veer , M. van Egmond , F. B. Niessen , R. H. Beelen , Immunobiology 2011, 216, 753. 21281986 10.1016/j.imbio.2011.01.001

[55] M. L. Novak , T. J. Koh , J. Leukoc. Biol. 2013, 93, 875. 23505314 10.1189/jlb.1012512 PMC3656331

[56] D. Kwon , B. G. Cha , Y. Cho , J. Min , E. B. Park , S. J. Kang , J. Kim , Nano Lett. 2017, 17, 2747. 28422506 10.1021/acs.nanolett.6b04130

[57] O. R. Mahon , D. C. Browe , T. Gonzalez‐Fernandez , P. Pitacco , I. T. Whelan , S. Von Euw , C. Hobbs , V. Nicolosi , K. T. Cunningham , K. H. G. Mills , D. J. Kelly , A. Dunne , Biomaterials 2020, 239, 119833. 32062479 10.1016/j.biomaterials.2020.119833

[58] D. Huang , K. Xu , X. Huang , N. Lin , Y. Ye , S. Lin , J. Zhang , J. Shao , S. Chen , M. Shi , X. Zhou , P. Lin , Y. Xue , C. Yu , X. Yu , Z. Ye , K. Cheng , Small 2022, 18, 2203680. 10.1002/smll.202203680 36031402

[59] J. Pajarinen , T. Lin , E. Gibon , Y. Kohno , M. Maruyama , K. Nathan , L. Lu , Z. Yao , S. B. Goodman , Biomaterials 2019, 196, 80. 29329642 10.1016/j.biomaterials.2017.12.025 PMC6028312

[60] K. L. Spiller , R. R. Anfang , K. J. Spiller , J. Ng , K. R. Nakazawa , J. W. Daulton , G. Vunjak‐Novakovic , Biomaterials 2014, 35, 4477. 24589361 10.1016/j.biomaterials.2014.02.012 PMC4000280

[61] Y. J. Huang , K. C. Hung , H. S. Hung , S. H. Hsu , ACS Appl. Mater. Interfaces 2018, 10, 19436. 29775050 10.1021/acsami.8b04718

[62] X. Bai , D. Chen , Y. Dai , S. Liang , B. Song , J. Guo , B. Dai , D. Zhang , L. Feng , Nanomedicine 2021, 38, 102457. 34400295 10.1016/j.nano.2021.102457

[63] J. Ping , C. Zhou , Y. Dong , X. Wu , X. Huang , B. Sun , B. Zeng , F. Xu , W. Liang , Mol. Immunol. 2021, 138, 110. 34392109 10.1016/j.molimm.2021.08.003

[64] Y. Niu , Z. Wang , Y. Shi , L. Dong , C. Wang , Bioact. Mater. 2021, 6, 244. 32913932 10.1016/j.bioactmat.2020.08.012 PMC7451865

[65] T. A. Wynn , K. M. Vannella , Immunity 2016, 44, 450. 26982353 10.1016/j.immuni.2016.02.015 PMC4794754

[66] L. Xia , T. Wu , L. Chen , P. Mei , L. Liu , R. Li , M. Shu , Z. Huan , C. Wu , B. Fang , Adv. Healthcare Mater. 2023, 12, 2302054. 10.1002/adhm.202302054 37842937

[67] A. Das , M. Sinha , S. Datta , M. Abas , S. Chaffee , C. K. Sen , S. Roy , Am J Pathol 2015, 185, 2596. 26118749 10.1016/j.ajpath.2015.06.001 PMC4607753

[68] K. L. Spiller , S. Nassiri , C. E. Witherel , R. R. Anfang , J. Ng , K. R. Nakazawa , T. Yu , G. Vunjak‐Novakovic , Biomaterials 2015, 37, 194. 25453950 10.1016/j.biomaterials.2014.10.017 PMC4312192

[69] X. Zhang , J. Y. Chen , X. Pei , L. X. Yang , L. Wang , L. N. Chen , G. M. Yang , X. B. Pei , Q. B. Wan , J. Wang , Chin. Chem. Lett. 2024, 35, 108889.

[70] C. Qin , H. Zhang , L. Chen , M. Zhang , J. Ma , H. Zhuang , Z. Huan , Y. Xiao , C. Wu , Adv. Healthcare Mater. 2023, 12, 2201923. 10.1002/adhm.202201923 36748277

[71] S. Liu , L. Yao , Y. Wang , Y. Li , Y. Jia , Y. Yang , N. Li , Y. Hu , D. Kong , X. Dong , K. Wang , M. Zhu , Bioact. Mater. 2023, 21, 464. 36185748 10.1016/j.bioactmat.2022.08.018 PMC9486249

[72] T. D. Smith , R. R. Nagalla , E. Y. Chen , W. F. Liu , Adv. Drug Deliv. Rev. 2017, 114, 193. 28449872 10.1016/j.addr.2017.04.012

[73] H. Kim , S. Y. Wang , G. Kwak , Y. Yang , I. C. Kwon , S. H. Kim , Adv. Sci. 2019, 6, 1900513. 10.1002/advs.201900513 PMC6794619 31637157

[74] A. Stahl , D. Hao , J. Barrera , D. Henn , S. Lin , S. Moeinzadeh , S. Kim , W. Maloney , G. Gurtner , A. Wang , Y. P. Yang , Bioact. Mater. 2023, 19, 167. 35510174 10.1016/j.bioactmat.2022.04.004 PMC9034314

[75] E. Vergadi , E. Ieronymaki , K. Lyroni , K. Vaporidi , C. Tsatsanis , J. Immunol. 2017, 198, 1006. 28115590 10.4049/jimmunol.1601515

[76] E. Bosch‐Rue , L. Diez‐Tercero , J. O. Buitrago , E. Castro , R. A. Perez , Acta Biomater. 2023, 166, 14. 37302735 10.1016/j.actbio.2023.06.001

[77] S. Bolamperti , I. Villa , A. Rubinacci , Bone Res. 2022, 10, 48. 35851054 10.1038/s41413-022-00219-8 PMC9293977

[78] Y. Xu , Y. Yang , Z. Hua , S. Li , Z. Yang , Q. Liu , G. Fu , P. Ji , Q. Wu , Biomaterials 2021, 275, 120890. 34130144 10.1016/j.biomaterials.2021.120890

[79] Y. Shin , Y. Won , J. I. Yang , J. S. Chun , Cell Death Dis. 2019, 10, 47. 30718470 10.1038/s41419-018-1284-4 PMC6362050

[80] T. Ono , T. Nakashima , Histochem. Cell Biol. 2018, 149, 325. 29392395 10.1007/s00418-018-1636-2

[81] H. Takayanagi , J. Bone Miner. Metab. 2021, 39, 13. 33385253 10.1007/s00774-020-01191-1

[82] H. Takayanagi , Nat. Rev. Rheumatol. 2015, 11, 74. 25561367 10.1038/nrrheum.2014.219

[83] M. Wu , Y. Zhang , P. Wu , F. Chen , Z. Yang , S. Zhang , L. Xiao , L. Cai , C. Zhang , Y. Chen , Z. Deng , NPJ Regen. Med. 2022, 7, 29. 35562356 10.1038/s41536-022-00224-9 PMC9106696

[84] T. Li , M. Peng , Z. Yang , X. Zhou , Y. Deng , C. Jiang , M. Xiao , J. Wang , Acta Biomater. 2018, 71, 96. 29549051 10.1016/j.actbio.2018.03.012

[85] P. Leucht , S. Lee , N. Yim , Biomaterials 2019, 196, 46. 29573821 10.1016/j.biomaterials.2018.03.029

[86] Z. Chen , Y. Liu , B. Sun , H. Li , J. Dong , L. Zhang , L. Wang , P. Wang , Y. Zhao , C. Chen , Small 2014, 10, 2362. 24619705 10.1002/smll.201302825

[87] L. Liu , H. Chen , X. Zhao , Q. Han , Y. Xu , Y. Liu , A. Zhang , Y. Li , W. Zhang , B. Chen , J. Wang , Mater. Today Bio 2025, 30, 101410. 10.1016/j.mtbio.2024.101410 PMC11731593 39811613

[88] L.‐B. Jiang , S.‐L. Ding , W. Ding , D.‐H. Su , F.‐X. Zhang , T.‐W. Zhang , X.‐F. Yin , L. Xiao , Y.‐L. Li , F.‐L. Yuan , J. Dong , Chem. Eng. J. 2021, 418, 129323.

[89] M. M. Martino , K. Maruyama , G. A. Kuhn , T. Satoh , O. Takeuchi , R. Muller , S. Akira , Nat. Commun. 2016, 7, 11051. 27001940 10.1038/ncomms11051 PMC4804175

[90] T. D. Zaveri , J. S. Lewis , N. V. Dolgova , M. J. Clare‐Salzler , B. G. Keselowsky , Biomaterials 2014, 35, 3504. 24462356 10.1016/j.biomaterials.2014.01.007 PMC3970928

[91] Z. Xu , L. Wu , Y. Tang , K. Xi , J. Tang , Y. Xu , J. Xu , J. Lu , K. Guo , Y. Gu , L. Chen , Adv. Healthcare Mater. 2023, 12, 2201661. 10.1002/adhm.202201661 PMC11469314 36189833

[92] L. Liu , Y. Shang , C. Li , Y. Jiao , Y. Qiu , C. Wang , Y. Wu , Q. Zhang , F. Wang , Z. Yang , L. Wang , Adv. Healthcare Mater. 2021, 10, 2101195. 10.1002/adhm.202101195 34350724

[93] J. O. Abaricia , A. H. Shah , M. Chaubal , K. M. Hotchkiss , R. Olivares‐Navarrete , Biomaterials 2020, 243, 119920. 32179303 10.1016/j.biomaterials.2020.119920 PMC7191325

[94] Q. L. Ma , L. Z. Zhao , R. R. Liu , B. Q. Jin , W. Song , Y. Wang , Y. S. Zhang , L. H. Chen , Y. M. Zhang , Biomaterials 2014, 35, 9853. 25201737 10.1016/j.biomaterials.2014.08.025

[95] R. S. B. Lee , S. M. Hamlet , H. J. Moon , S. Ivanovski , Biomaterials 2021, 267, 120464. 33130322 10.1016/j.biomaterials.2020.120464

[96] R. Whitaker , B. Hernaez‐Estrada , R. M. Hernandez , E. Santos‐Vizcaino , K. L. Spiller , Chem. Rev. 2021, 121, 11305. 34415742 10.1021/acs.chemrev.0c00895

[97] M. Li , J. Wu , W. Geng , P. Gao , Y. Yang , X. Li , K. Xu , Q. Liao , K. Cai , Bioact. Mater. 2024, 31, 355. 37663618 10.1016/j.bioactmat.2023.08.017 PMC10474585

[98] D. K. Patel , S. D. Dutta , J. Hexiu , K. Ganguly , K. T. Lim , Carbohydr. Polym. 2022, 281, 119077. 35074128 10.1016/j.carbpol.2021.119077

[99] T. L. Lopez‐Silva , D. G. Leach , A. Azares , I. C. Li , D. G. Woodside , J. D. Hartgerink , Biomaterials 2020, 231, 119667. 31855625 10.1016/j.biomaterials.2019.119667 PMC7049098

[100] C. Y. Wang , Z. X. Qin , Y. Wei , J. X. Hao , Y. F. Zhu , F. Zhao , K. Jiao , H. Ehrlich , F. R. Tay , L. N. Niu , Acta Biomater. 2023, 162, 32. 36967055 10.1016/j.actbio.2023.03.031

[101] J. Lee , H. Byun , S. K. Madhurakkat Perikamana , S. Lee , H. Shin , Adv. Healthcare Mater. 2019, 8, 1801106. 10.1002/adhm.201801106 30328293

[102] X. Li , Z. Li , P. Wang , G. Lu , L. Tong , Q. Liu , Y. Chen , J. Lin , E. Luo , J. Liang , Q. Jiang , Y. Fan , X. Zhang , Y. Sun , Adv. Funct. Mater. 2023, 33, 2212738.

[103] J. Zhu , Z. Li , Y. Zou , G. Lu , A. Ronca , U. D'Amora , J. Liang , Y. Fan , X. Zhang , Y. Sun , J. Leath. Sci. Eng. 2022, 4, 30.

[104] X. Bai , S. Y. Lü , Z. Cao , C. M. Gao , H. G. Duan , X. B. Xu , L. Sun , N. N. Gao , C. Feng , M. Z. Liu , Chem. Eng. J. 2016, 288, 546.

[105] S. Liu , L. Zhang , Z. Li , F. Gao , Q. Zhang , A. Bianco , H. Liu , S. Ge , B. Ma , Adv. Funct. Mater. 2023, 34, 2306534.

[106] K. Sadtler , M. T. Wolf , S. Ganguly , C. A. Moad , L. Chung , S. Majumdar , F. Housseau , D. M. Pardoll , J. H. Elisseeff , Biomaterials 2019, 192, 405. 30500722 10.1016/j.biomaterials.2018.11.002

[107] A. K. Refai , M. Textor , D. M. Brunette , J. D. Waterfield , J. Biomed. Mater. Res. A 2004, 70, 194. 15227664 10.1002/jbm.a.30075

[108] S. Franz , S. Rammelt , D. Scharnweber , J. C. Simon , Biomaterials 2011, 32, 6692. 21715002 10.1016/j.biomaterials.2011.05.078

[109] L. Chung , D. R. Maestas Jr. , F. Housseau , J. H. Elisseeff , Adv. Drug Deliv. Rev. 2017, 114, 184. 28712923 10.1016/j.addr.2017.07.006

[110] R. Liu , S. Chen , P. Huang , G. Liu , P. Luo , Z. Li , Y. Xiao , Z. Chen , Z. Chen , Adv. Funct. Mater. 2020, 30, 1910672.

[111] D. Hachim , S. T. LoPresti , C. C. Yates , B. N. Brown , Biomaterials 2017, 112, 95. 27760399 10.1016/j.biomaterials.2016.10.019 PMC5121003

[112] S. S. Jin , D. Q. He , D. Luo , Y. Wang , M. Yu , B. Guan , Y. Fu , Z. X. Li , T. Zhang , Y. H. Zhou , C. Y. Wang , Y. Liu , ACS Nano 2019, 13, 6581. 31125522 10.1021/acsnano.9b00489

[113] L. Ou , X. Tan , S. Qiao , J. Wu , Y. Su , W. Xie , N. Jin , J. He , R. Luo , X. Lai , W. Liu , Y. Zhang , F. Zhao , J. Liu , Y. Kang , L. Shao , ACS Nano 2023, 17, 18669. 37768738 10.1021/acsnano.3c03857

[114] L. Bai , Y. Liu , Z. Du , Z. Weng , W. Yao , X. Zhang , X. Huang , X. Yao , R. Crawford , R. Hang , D. Huang , B. Tang , Y. Xiao , Acta Biomater. 2018, 76, 344. 29908975 10.1016/j.actbio.2018.06.023

[115] K. Wang , W. D. Hou , X. Wang , C. Han , I. Vuletic , N. Su , W. X. Zhang , Q. S. Ren , L. Chen , Y. Luo , Biomaterials 2016, 102, 249. 27344368 10.1016/j.biomaterials.2016.06.028

[116] L. Burroughs , M. H. Amer , M. Vassey , B. Koch , G. P. Figueredo , B. Mukonoweshuro , P. Mikulskis , A. Vasilevich , S. Vermeulen , I. L. Dryden , D. A. Winkler , A. M. Ghaemmaghami , F. Rose , J. de Boer , M. R. Alexander , Biomaterials 2021, 271, 120740. 33714019 10.1016/j.biomaterials.2021.120740

[117] Y. Yu , X. Shen , Z. Luo , Y. Hu , M. Li , P. Ma , Q. Ran , L. Dai , Y. He , K. Cai , Biomaterials 2018, 167, 44. 29554480 10.1016/j.biomaterials.2018.03.024

[118] Z. Chen , A. Bachhuka , S. Han , F. Wei , S. Lu , R. M. Visalakshan , K. Vasilev , Y. Xiao , ACS Nano 2017, 11, 4494. 28414902 10.1021/acsnano.6b07808

[119] J. Huang , R. Li , J. Yang , M. Cai , Y. Lee , A. Wang , B. Cheng , Y. Wang , Bioact. Mater. 2021, 6, 3164. 33778196 10.1016/j.bioactmat.2021.02.023 PMC7970012

[120] X. Yu , Y. Wang , M. Zhang , H. Ma , C. Feng , B. Zhang , X. Wang , B. Ma , Q. Yao , C. Wu , Acta Biomater. 2023, 156, 222. 36100177 10.1016/j.actbio.2022.09.008

[121] E. Saino , M. L. Focarete , C. Gualandi , E. Emanuele , A. I. Cornaglia , M. Imbriani , L. Visai , Biomacromolecules 2011, 12, 1900. 21417396 10.1021/bm200248h

[122] C. Yang , C. Zhao , X. Wang , M. Shi , Y. Zhu , L. Jing , C. Wu , J. Chang , Nanoscale 2019, 11, 17699. 31545331 10.1039/c9nr05730g

[123] B. Zhang , F. Han , Y. Wang , Y. Sun , M. Zhang , X. Yu , C. Qin , H. Zhang , C. Wu , Adv. Sci. 2022, 9, 2200670. 10.1002/advs.202200670 PMC9218778 35478383

[124] Q. Zhang , J. W. Hwang , J. H. Oh , C. H. Park , S. H. Chung , Y. S. Lee , J. H. Baek , H. M. Ryoo , K. M. Woo , Biomaterials 2017, 149, 77. 29017079 10.1016/j.biomaterials.2017.10.007

[125] D. O. Costa , P. D. Prowse , T. Chrones , S. M. Sims , D. W. Hamilton , A. S. Rizkalla , S. J. Dixon , Biomaterials 2013, 34, 7215. 23830579 10.1016/j.biomaterials.2013.06.014

[126] Y. Zheng , X. Chen , Q. Zhang , L. Yang , Q. Chen , Z. Chen , Y. Wang , D. Wu , Adv. Healthcare Mater. 2024, 13, 2302640. 10.1002/adhm.202302640 37924329

[127] S. Camarero‐Espinosa , M. Carlos‐Oliveira , H. Liu , J. F. Mano , N. Bouvy , L. Moroni , Adv. Healthcare Mater. 2022, 11, 2101415. 10.1002/adhm.202101415 PMC11468864 34719861

[128] S. W. Choi , Y. Zhang , M. R. Macewan , Y. Xia , Adv. Healthcare Mater. 2013, 2, 145. 10.1002/adhm.201200106 PMC3541475 23184495

[129] A. Petersen , A. Princ , G. Korus , A. Ellinghaus , H. Leemhuis , A. Herrera , A. Klaumunzer , S. Schreivogel , A. Woloszyk , K. Schmidt‐Bleek , S. Geissler , I. Heschel , G. N. Duda , Nat. Commun. 2018, 9, 4430. 30361486 10.1038/s41467-018-06504-7 PMC6202397

[130] Y. Yin , X. T. He , J. Wang , R. X. Wu , X. Y. Xu , Y. L. Hong , B. M. Tian , F. M. Chen , Appl. Mater. Today 2020, 18, 100466.

[131] Z. Chen , S. Ni , S. Han , R. Crawford , S. Lu , F. Wei , J. Chang , C. Wu , Y. Xiao , Nanoscale 2017, 9, 706. 27959374 10.1039/c6nr06421c

[132] K. Garg , N. A. Pullen , C. A. Oskeritzian , J. J. Ryan , G. L. Bowlin , Biomaterials 2013, 34, 4439. 23515178 10.1016/j.biomaterials.2013.02.065 PMC3623371

[133] L. Zhu , D. Luo , Y. Liu , Int J Oral Sci 2020, 12, 6. 32024822 10.1038/s41368-020-0073-y PMC7002518

[134] W. Li , F. Dai , S. Zhang , F. Xu , Z. Xu , S. Liao , L. Zeng , L. Song , F. Ai , ACS Appl. Mater. Interfaces 2022, 14, 20693. 35500207 10.1021/acsami.2c02001

[135] X. Liu , M. Chen , J. Luo , H. Zhao , X. Zhou , Q. Gu , H. Yang , X. Zhu , W. Cui , Q. Shi , Biomaterials 2021, 276, 121037. 34325336 10.1016/j.biomaterials.2021.121037

[136] H. M. Rostam , S. Singh , F. Salazar , P. Magennis , A. Hook , T. Singh , N. E. Vrana , M. R. Alexander , A. M. Ghaemmaghami , Immunobiology 2016, 221, 1237. 27349596 10.1016/j.imbio.2016.06.010

[137] K. M. Hotchkiss , G. B. Reddy , S. L. Hyzy , Z. Schwartz , B. D. Boyan , R. Olivares‐Navarrete , Acta Biomater. 2016, 31, 425. 26675126 10.1016/j.actbio.2015.12.003 PMC4728000

[138] J. Vlacic‐Zischke , S. M. Hamlet , T. Friis , M. S. Tonetti , S. Ivanovski , Biomaterials 2011, 32, 665. 20933273 10.1016/j.biomaterials.2010.09.025

[139] L. Lv , Y. Xie , K. Li , T. Hu , X. Lu , Y. Cao , X. Zheng , Adv. Healthcare Mater. 2018, 7, 1800675. 10.1002/adhm.201800675 30106513

[140] R. Sridharan , B. Cavanagh , A. R. Cameron , D. J. Kelly , F. J. O'Brien , Acta Biomater. 2019, 89, 47. 30826478 10.1016/j.actbio.2019.02.048

[141] T. Okamoto , Y. Takagi , E. Kawamoto , E. J. Park , H. Usuda , K. Wada , M. Shimaoka , Exp. Cell Res. 2018, 367, 264. 29627321 10.1016/j.yexcr.2018.04.005

[142] A. K. Blakney , M. D. Swartzlander , S. J. Bryant , J. Biomed. Mater. Res. A 2012, 100, 1375. 22407522 10.1002/jbm.a.34104 PMC3339197

[143] M. Li , X. Chu , D. Wang , L. Jian , L. Liu , M. Yao , D. Zhang , Y. Zheng , X. Liu , Y. Zhang , F. Peng , Biomaterials 2022, 282, 121408. 35189460 10.1016/j.biomaterials.2022.121408

[144] M. Bartneck , H. A. Keul , S. Singh , K. Czaja , J. Bornemann , M. Bockstaller , M. Moeller , G. Zwadlo‐Klarwasser , J. Groll , ACS Nano 2010, 4, 3073. 20507158 10.1021/nn100262h

[145] W. Zhang , F. Zhao , D. Huang , X. Fu , X. Li , X. Chen , ACS Appl. Mater. Interfaces 2016, 8, 30747. 27779382 10.1021/acsami.6b10378

[146] Z. Zhong , X. Wu , Y. Wang , M. Li , Y. Li , X. Liu , X. Zhang , Z. Lan , J. Wang , Y. Du , S. Zhang , Bioact. Mater. 2022, 10, 195. 34901539 10.1016/j.bioactmat.2021.09.013 PMC8636740

[147] M. Shi , L. Xia , Z. Chen , F. Lv , H. Zhu , F. Wei , S. Han , J. Chang , Y. Xiao , C. Wu , Biomaterials 2017, 144, 176. 28837959 10.1016/j.biomaterials.2017.08.027

[148] H. Liu , M. Lin , X. Liu , Y. Zhang , Y. Luo , Y. Pang , H. Chen , D. Zhu , X. Zhong , S. Ma , Y. Zhao , Q. Yang , X. Zhang , Bioact. Mater. 2020, 5, 844. 32637748 10.1016/j.bioactmat.2020.06.005 PMC7327760

[149] L. Yang , I. Ullah , K. Yu , W. Zhang , J. Zhou , T. Sun , L. Shi , S. Yao , K. Chen , X. Zhang , X. Guo , Biofabrication 2021, 13, 035007. 10.1088/1758-5090/abcf8d 33260162

[150] F. Zhao , B. Lei , X. Li , Y. Mo , R. Wang , D. Chen , X. Chen , Biomaterials 2018, 178, 36. 29908343 10.1016/j.biomaterials.2018.06.004

[151] S. Li , L. Zhang , C. Liu , J. Kim , K. Su , T. Chen , L. Zhao , X. Lu , H. Zhang , Y. Cui , X. Cui , F. Yuan , Bioact. Mater. 2023, 23, 101. 36406252 10.1016/j.bioactmat.2022.10.021 PMC9664355

[152] N. H. Lee , M. S. Kang , T. H. Kim , D. S. Yoon , N. Mandakhbayar , S. B. Jo , H. S. Kim , J. C. Knowles , J. H. Lee , H. W. Kim , Biomaterials 2021, 276, 121025. 34298444 10.1016/j.biomaterials.2021.121025

[153] J. Sugimoto , A. M. Romani , A. M. Valentin‐Torres , A. A. Luciano , C. M. Ramirez Kitchen , N. Funderburg , S. Mesiano , H. B. Bernstein , J. Immunol. 2012, 188, 6338. 22611240 10.4049/jimmunol.1101765 PMC3884513

[154] X. Li , B. Dai , J. Guo , Y. Zhu , J. Xu , S. Xu , Z. Yao , L. Chang , Y. Li , X. He , D. H. K. Chow , S. Zhang , H. Yao , W. Tong , T. Ngai , L. Qin , ACS Nano 2022, 16, 18071. 36108267 10.1021/acsnano.2c04747

[155] T. Qi , J. Weng , F. Yu , W. Zhang , G. Li , H. Qin , Z. Tan , H. Zeng , Biol. Trace Elem. Res. 2021, 199, 559. 32449009 10.1007/s12011-020-02183-y

[156] Z. W. Zheng , Y. H. Chen , B. Guo , Y. Wang , W. Liu , J. Sun , X. S. Wang , Chem. Eng. J. 2020, 396, 125241.

[157] W. Qiao , K. H. M. Wong , J. Shen , W. Wang , J. Wu , J. Li , Z. Lin , Z. Chen , J. P. Matinlinna , Y. Zheng , S. Wu , X. Liu , K. P. Lai , Z. Chen , Y. W. Lam , K. M. C. Cheung , K. W. K. Yeung , Nat. Commun. 2021, 12, 2885. 34001887 10.1038/s41467-021-23005-2 PMC8128914

[158] Z. Zhao , G. Li , H. Ruan , K. Chen , Z. Cai , G. Lu , R. Li , L. Deng , M. Cai , W. Cui , ACS Nano 2021, 15, 13041. 34342981 10.1021/acsnano.1c02147

[159] J. Li , C. Deng , W. Liang , F. Kang , Y. Bai , B. Ma , C. Wu , S. Dong , Bioact. Mater. 2021, 6, 3839. 33898880 10.1016/j.bioactmat.2021.03.039 PMC8050801

[160] M. Shi , Z. Chen , S. Farnaghi , T. Friis , X. Mao , Y. Xiao , C. Wu , Acta Biomater. 2016, 30, 334. 26596565 10.1016/j.actbio.2015.11.033

[161] M. L. Zhou , X. L. Wu , J. X. Luo , G. Z. Yang , Y. Z. Lu , S. H. Lin , F. Jiang , W. J. Zhang , X. Q. Jiang , Chem. Eng. J. 2021, 422, 130147.

[162] A. Jacobs , G. Renaudin , C. Forestier , J. M. Nedelec , S. Descamps , Acta Biomater. 2020, 117, 21. 33007487 10.1016/j.actbio.2020.09.044

[163] F. Suska , C. Gretzer , M. Esposito , L. Emanuelsson , A. Wennerberg , P. Tengvall , P. Thomsen , Biomaterials 2005, 26, 519. 15276360 10.1016/j.biomaterials.2004.02.066

[164] R. Lin , C. Deng , X. Li , Y. Liu , M. Zhang , C. Qin , Q. Yao , L. Wang , C. Wu , Theranostics 2019, 9, 6300. 31534552 10.7150/thno.36120 PMC6735521

[165] M. He , H. Wang , Q. Han , X. Shi , S. He , J. Sun , Z. Zhu , X. Gan , Y. Deng , Biomaterials 2023, 303, 122355. 37948855 10.1016/j.biomaterials.2023.122355

[166] D. W. Zhao , C. M. Du , K. Q. Zuo , Y. X. Zhao , X. Q. Xu , Y. B. Li , S. Tian , H. R. Yang , Y. P. Lu , L. Cheng , G. Y. Xiao , Adv. Healthcare Mater. 2023, 12, 2202537. 10.1002/adhm.202202537 36528867

[167] W. Liu , J. Li , M. Cheng , Q. Wang , K. W. K. Yeung , P. K. Chu , X. Zhang , Adv. Sci. 2018, 5, 1800749. 10.1002/advs.201800749 PMC6193167 30356934

[168] A. Lao , J. Wu , D. Li , A. Shen , Y. Li , Y. Zhuang , K. Lin , J. Wu , J. Liu , Small 2023, 19, 2206919. 10.1002/smll.202206919 37183293

[169] L. Zheng , Z. Zhuang , Y. Li , T. Shi , K. Fu , W. Yan , L. Zhang , P. Wang , L. Li , Q. Jiang , Bioact. Mater. 2022, 14, 250. 35310348 10.1016/j.bioactmat.2021.11.012 PMC8897644

[170] Y. Kong , F. Liu , B. J. Ma , W. H. Wang , L. Li , X. Y. Xu , Z. Y. Sun , H. R. Yang , Y. H. Sang , D. Li , G. Li , C. Liu , S. H. Wang , H. Liu , Nano Res. 2022, 15, 1153.

[171] C. Yang , W. Wang , K. Zhu , W. Liu , Y. Luo , X. Yuan , J. Wang , T. Cheng , X. Zhang , Int. J. Nanomed. 2019, 14, 7475. 10.2147/IJN.S210834 PMC6750619 31571859

[172] E. L. de Souza Cruz , F. J. de Souza Jr. , L. M. de Menezes , F. M. Tuji , J. T. Carneiro Jr. , Sci. Rep. 2020, 10, 12690. 32728040 10.1038/s41598-020-68932-0 PMC7391678

[173] K. Zheng , W. Niu , B. Lei , A. R. Boccaccini , Acta Biomater. 2021, 133, 168. 34418539 10.1016/j.actbio.2021.08.023

[174] Y. Huang , C. Wu , X. Zhang , J. Chang , K. Dai , Acta Biomater. 2018, 66, 81. 28864248 10.1016/j.actbio.2017.08.044

[175] Y. Zhou , Z. Hu , W. Jin , H. Wu , M. Zuo , C. Shao , Y. Lan , Y. Shi , R. Tang , Z. Chen , Z. Xie , J. Shi , Adv. Healthcare Mater. 2023, 12, 2201548. 10.1002/adhm.202201548 36867636

[176] X. Sun , Z. Ma , X. Zhao , W. Jin , C. Zhang , J. Ma , L. Qiang , W. Wang , Q. Deng , H. Yang , J. Zhao , Q. Liang , X. Zhou , T. Li , J. Wang , Bioact. Mater. 2021, 6, 757. 33024897 10.1016/j.bioactmat.2020.08.030 PMC7522044

[177] X. Q. Hao , X. W. Zhang , Y. Hu , C. X. Ren , C. W. Liu , L. Wang , Y. J. Zhou , S. S. Wang , H. Y. Luo , G. X. Yan , X. Wang , X. M. Wang , F. L. Ren , C. Shi , W. L. Song , H. C. Sun , Chin. Chem. Lett. 2023, 34, 107965.

[178] D. M. Vasconcelos , R. M. Goncalves , C. R. Almeida , I. O. Pereira , M. I. Oliveira , N. Neves , A. M. Silva , A. C. Ribeiro , C. Cunha , A. R. Almeida , C. C. Ribeiro , A. M. Gil , E. Seebach , K. L. Kynast , W. Richter , M. Lamghari , S. G. Santos , M. A. Barbosa , Biomaterials 2016, 111, 163. 27728815 10.1016/j.biomaterials.2016.10.004

[179] M. Zou , J. Sun , Z. Xiang , Adv. Healthcare Mater. 2021, 10, 2001502. 10.1002/adhm.202001502 33464711

[180] Q. Chen , J. Li , F. Han , Q. Meng , H. Wang , Q. Wei , Z. Li , F. Li , E. Xie , X. Qin , S. Chen , W. Wang , C. Liu , B. Li , F. Han , Adv. Funct. Mater. 2022, 32, 2201067.

[181] Q. X. Chen , J. Y. Li , F. Han , Q. C. Meng , H. Wang , W. Qiang , Z. X. Li , F. F. Li , E. Xie , X. Y. Qin , S. Chen , W. S. Wang , C. Y. Liu , B. Li , F. X. Han , Adv. Funct. Mater. 2022, 32, 2201067.

[182] Y. Liu , M. Sun , T. Wang , X. Chen , H. Wang , View 2020, 2, 20200069.

[183] A. Geraili , M. Xing , K. Mequanint , View 2021, 2, 20200126.

[184] P. F. Wei , Z. Y. Yuan , W. Jing , Y. Q. Huang , Q. Cai , B. B. Guan , Z. H. Liu , X. Zhang , J. P. Mao , D. F. Chen , X. P. Yang , Chem. Eng. J. 2019, 368, 577.

[185] C. Mu , Y. Hu , L. Huang , X. Shen , M. Li , L. Li , H. Gu , Y. Yu , Z. Xia , K. Cai , Mater. Sci. Eng. C Mater. Biol. Appl. 2018, 82, 345. 29025668 10.1016/j.msec.2017.08.056

[186] Y. Cui , T. Zhu , D. Li , Z. Li , Y. Leng , X. Ji , H. Liu , D. Wu , J. Ding , Adv. Healthcare Mater. 2019, 8, 1901073. 10.1002/adhm.201901073 31693315

[187] R. Bosco , M. Iafisco , A. Tampieri , J. A. Jansen , S. C. G. Leeuwenburgh , J. J. J. P. van den Beucken , Appl. Surf. Sci. 2015, 328, 516.

[188] Y. Zhao , H. Kang , X. Wu , P. Zhuang , R. Tu , T. Goto , F. Li , H. Dai , Adv. Healthcare Mater. 2023, 12, 2203099. 10.1002/adhm.202203099 36780559

[189] Q. Y. Zhang , J. Tan , K. Huang , R. Nie , Z. Y. Feng , C. Y. Zou , Q. J. Li , J. Chen , N. Sheng , B. Q. Qin , Z. P. Gu , L. M. Liu , H. Q. Xie , Carbohydr. Polym. 2023, 305, 120546. 36737196 10.1016/j.carbpol.2023.120546

[190] P. C. Tseng , S. M. Hou , R. J. Chen , H. W. Peng , C. F. Hsieh , M. L. Kuo , M. L. Yen , J. Bone Miner. Res. 2011, 26, 2552. 21713995 10.1002/jbmr.460

[191] X. Han , J. Shen , S. Chen , Z. Cai , Y. Zhu , W. Yi , K. Li , W. Cai , B. Tao , W. Cui , D. Bai , Biomaterials 2023, 295, 122057. 36805244 10.1016/j.biomaterials.2023.122057

[192] L. Li , Q. Li , L. Gui , Y. Deng , L. Wang , J. Jiao , Y. Hu , X. Lan , J. Hou , Y. Li , D. Lu , Bioact. Mater. 2023, 19, 24. 35415312 10.1016/j.bioactmat.2022.03.037 PMC8980440

[193] S. Xiao , J. Wei , S. Jin , X. Xia , L. Yuan , Q. Zou , Y. Zuo , J. Li , Y. Li , Adv. Funct. Mater. 2022, 33.

[194] Y. Liu , Z. Yang , L. Wang , L. Sun , B. Y. S. Kim , W. Jiang , Y. Yuan , C. Liu , Adv. Sci. 2021, 8, 2100143. 10.1002/advs.202100143 PMC8188258 34105266

[195] L. Ouyang , B. Chen , X. Liu , D. Wang , Y. Li , Y. Liao , K. W. K. Yeung , X. Liu , Bioact. Mater. 2023, 21, 520. 36185735 10.1016/j.bioactmat.2022.09.005 PMC9508162

[196] B. Li , R. Huang , J. Ye , L. Liu , L. Qin , J. H. Zhou , Y. F. Zheng , S. L. Wu , Y. Han , Chem. Eng. J. 2021, 403, 126323.

[197] X. Qi , E. Cai , Y. Xiang , C. Zhang , X. Ge , J. Wang , Y. Lan , H. Xu , R. Hu , J. Shen , Adv. Mater. 2023, 35, 2306632. 10.1002/adma.202306632 37803944

[198] Z. He , C. Sun , Y. Ma , X. Chen , Y. Wang , K. Chen , F. Xie , Y. Zhang , Y. Yuan , C. Liu , Adv. Mater. 2024, 36, 2306552. 10.1002/adma.202306552 37848015

[199] Y. Wang , J. Wang , R. Gao , X. Liu , Z. Feng , C. Zhang , P. Huang , A. Dong , D. Kong , W. Wang , Biomaterials 2022, 285, 121538. 35504180 10.1016/j.biomaterials.2022.121538

[200] L. Wu , Y. Kim , G. M. Seon , S. H. Choi , H. C. Park , G. Son , S. M. Kim , B. S. Lim , H. C. Yang , Biomaterials 2021, 279, 121239. 34753037 10.1016/j.biomaterials.2021.121239

[201] L. Wang , L. Chen , J. Wang , L. Wang , C. Gao , B. Li , Y. Wang , J. Wu , C. Quan , Chin. Chem. Lett. 2022, 33, 1956.

[202] H. Egusa , Y. Kaneda , Y. Akashi , Y. Hamada , T. Matsumoto , M. Saeki , D. K. Thakor , Y. Tabata , N. Matsuura , H. Yatani , Biomaterials 2009, 30, 4676. 19520427 10.1016/j.biomaterials.2009.05.032

[203] S. Ma , C. Wang , Y. Dong , W. Jing , P. Wei , C. Peng , Z. Liu , B. Zhao , Y. Wang , ACS Appl. Mater. Interfaces 2022, 14, 38525. 35973165 10.1021/acsami.2c10242

[204] S. Nadine , I. Fernandes , S. G. Patricio , C. R. Correia , J. F. Mano , Adv. Healthcare Mater. 2022, 11, 2200651. 10.1002/adhm.202200651 35904030

[205] N. Su , C. Villicana , D. Barati , P. Freeman , Y. Luo , F. Yang , Adv. Mater. 2023, 35, 2208781. 10.1002/adma.202208781 PMC10057912 36560890

[206] X. Huang , X. Xiong , J. Liu , Z. Zhao , X. Cen , Life Sci. 2020, 254, 117809. 32428598 10.1016/j.lfs.2020.117809

[207] D. Su , H. I. Tsai , Z. Xu , F. Yan , Y. Wu , Y. Xiao , X. Liu , Y. Wu , S. Parvanian , W. Zhu , J. E. Eriksson , D. Wang , H. Zhu , H. Chen , F. Cheng , J. Extracell. Ves. 2019, 9, 1709262. 10.1080/20013078.2019.1709262 PMC7580831 33133428

[208] A. Liu , S. Jin , C. Fu , S. Cui , T. Zhang , L. Zhu , Y. Wang , S. G. F. Shen , N. Jiang , Y. Liu , Int. J. Oral Sci. 2020, 12, 33. 33257654 10.1038/s41368-020-00100-6 PMC7705747

[209] Y. Chen , Y. Wu , L. Guo , S. Yuan , J. Sun , K. Zhao , J. Wang , R. An , J. Nanobiotechnol. 2023, 21, 98. 10.1186/s12951-023-01855-w PMC10029245 36941678

[210] M. Kang , C. C. Huang , Y. Lu , S. Shirazi , P. Gajendrareddy , S. Ravindran , L. F. Cooper , Bone 2020, 141, 115627. 32891867 10.1016/j.bone.2020.115627 PMC8107826

[211] S. C. Tao , X. R. Li , W. J. Wei , Z. Y. Wei , C. R. Zhang , F. Wang , H. Dawes , S. C. Guo , Biomaterials 2022, 283, 121465. 35286850 10.1016/j.biomaterials.2022.121465

[212] Y. K. Zhou , C. S. Han , Z. L. Zhu , P. Chen , Y. M. Wang , S. Lin , L. J. Chen , Z. M. Zhuang , Y. H. Zhou , R. L. Yang , Bioact. Mater. 2024, 31, 192. 37593496 10.1016/j.bioactmat.2023.08.006 PMC10429289

[213] B. Pan , Z. Zhang , X. Wu , G. Xian , X. Hu , M. Gu , L. Zheng , X. Li , L. Long , W. Chen , P. Sheng , Bioact. Mater. 2023, 26, 181. 36911207 10.1016/j.bioactmat.2023.02.028 PMC9999169

[214] Y. Zhang , M. Huo , Y. Wang , L. Xiao , J. Wu , Y. Ma , D. Zhang , X. Lang , X. Wang , J. Biol. Eng. 2022, 16, 22. 35996115 10.1186/s13036-022-00301-z PMC9394013

[215] Y. Kang , C. Xu , L. Meng , X. Dong , M. Qi , D. Jiang , Bioact Mater 2022, 18, 26. 35387167 10.1016/j.bioactmat.2022.02.012 PMC8961306

[216] J. J. Deng , X. Wang , W. H. Zhang , L. Y. Sun , X. X. Han , X. Q. Tong , L. M. Yu , J. D. Ding , L. Yu , Y. H. Liu , Adv. Funct. Mater. 2023, 33, 2211664.

[217] S. Chen , H. Wang , D. Liu , J. Bai , H. J. Haugen , B. Li , H. Yan , Bioact. Mater. 2023, 25, 176. 36817825 10.1016/j.bioactmat.2023.01.022 PMC9932297

[218] C. Hu , L. Yang , Y. Wang , MedComm – Biomater. Appl. 2022, 1, 23. 10.1002/mba2.30 PMC10983815 38562247

[219] P. Tian , L. Zhao , J. Kim , X. Li , C. Liu , X. Cui , T. Liang , Y. Du , X. Chen , H. Pan , Bioact. Mater. 2023, 26, 231. 36936808 10.1016/j.bioactmat.2023.02.023 PMC10020664

[220] C. Montoya , Y. Du , A. L. Gianforcaro , S. Orrego , M. Yang , P. I. Lelkes , Bone Res. 2021, 9, 12. 33574225 10.1038/s41413-020-00131-z PMC7878740

[221] Z. Li , Y. Zhou , T. Li , J. Zhang , H. Tian , View 2021, 3, 20200112.

[222] F. Zhang , M. Lv , S. Wang , M. Li , Y. Wang , C. Hu , W. Hu , X. Wang , X. Wang , Z. Liu , Z. Fan , J. Du , Y. Sun , Bioact. Mater. 2024, 31, 231. 37637084 10.1016/j.bioactmat.2023.08.008 PMC10450354

[223] Q. Song , D. Wang , H. Li , Z. Wang , S. Sun , Z. Wang , Y. Liu , S. Lin , G. Li , S. Zhang , P. Zhang , Bioact. Mater. 2024, 32, 304. 37876555 10.1016/j.bioactmat.2023.10.007 PMC10590728

[224] X. Sun , Y. Gao , Z. Li , J. He , Y. Wu , Biomaterials 2023, 295, 122051. 36812842 10.1016/j.biomaterials.2023.122051

[225] Y. Zhu , Q. Yang , M. Yang , X. Zhan , F. Lan , J. He , Z. Gu , Y. Wu , ACS Nano 2017, 11, 3690. 28314099 10.1021/acsnano.6b08193

[226] S. Hao , J. Meng , Y. Zhang , J. Liu , X. Nie , F. Wu , Y. Yang , C. Wang , N. Gu , H. Xu , Biomaterials 2017, 140, 16. 28623721 10.1016/j.biomaterials.2017.06.013

[227] S. Li , C. Wei , Curr. Stem Cell Res. Ther. 2020, 15, 428. 31893995 10.2174/1574888X15666200101122505

[228] Z. Liu , X. Wan , Z. L. Wang , L. Li , Adv. Mater. 2021, 33, 2007429. 10.1002/adma.202007429 34117803

[229] T. Vinikoor , G. K. Dzidotor , T. T. Le , Y. Liu , H. M. Kan , S. Barui , M. T. Chorsi , E. J. Curry , E. Reinhardt , H. Wang , P. Singh , M. A. Merriman , E. D'Orio , J. Park , S. Xiao , J. H. Chapman , F. Lin , C. S. Truong , S. Prasadh , L. Chuba , S. Killoh , S. W. Lee , Q. Wu , R. M. Chidambaram , K. W. H. Lo , C. T. Laurencin , T. D. Nguyen , Nat. Commun. 2023, 14, 6257. 37802985 10.1038/s41467-023-41594-y PMC10558537

[230] Z. C. Hu , J. Q. Lu , T. W. Zhang , H. F. Liang , H. Yuan , D. H. Su , W. Ding , R. X. Lian , Y. X. Ge , B. Liang , J. Dong , X. G. Zhou , L. B. Jiang , Bioact. Mater. 2023, 22, 1. 36203961 10.1016/j.bioactmat.2022.08.025 PMC9513113

[231] H. Wu , H. Dong , Z. Tang , Y. Chen , Y. Liu , M. Wang , X. Wei , N. Wang , S. Bao , D. Yu , Z. Wu , Z. Yang , X. Li , Z. Guo , L. Shi , Biomaterials 2023, 293, 121990. 36586147 10.1016/j.biomaterials.2022.121990

[232] J. Sun , D. Zhao , Y. Wang , P. Chen , C. Xu , H. Lei , K. Wo , J. Zhang , J. Wang , C. Yang , B. Su , Z. Jin , Z. Luo , L. Chen , ACS Nano 2023, 17, 22830. 37943709 10.1021/acsnano.3c07607

[233] X. Dai , B. C. Heng , Y. Bai , F. You , X. Sun , Y. Li , Z. Tang , M. Xu , X. Zhang , X. Deng , Bioact. Mater. 2021, 6, 2029. 33474514 10.1016/j.bioactmat.2020.12.020 PMC7787955

[234] H. Wang , R. T. Morales , X. Cui , J. Huang , W. Qian , J. Tong , W. Chen , Adv. Healthcare Mater. 2019, 8, 1801234. 10.1002/adhm.201801234 PMC6392032 30537061

[235] Y. L. Yu , J. J. Wu , C. C. Lin , X. Qin , F. R. Tay , L. Miao , B. L. Tao , Y. Jiao , Mil. Med. Res. 2023, 10, 21. 37143145 10.1186/s40779-023-00454-y PMC10158155

[236] A. Sun , X. Y. He , X. Ji , D. R. Hu , M. Pan , L. H. Zhang , Z. Y. Qian , Chin. Chem. Lett. 2021, 32, 2117.

[237] K. Xu , K. Li , Y. He , X. Li , C. Lin , J. Wu , S. Liu , Y. Ding , Y. Yang , S. Gou , P. Liu , K. Cai , Adv. Healthcare Mater. 2023, 12, 2300494. 10.1002/adhm.202300494 36929688

[238] M. Wu , H. Liu , D. Li , Y. Zhu , P. Wu , Z. Chen , F. Chen , Y. Chen , Z. Deng , L. Cai , Adv. Sci. 2024, 11, 2304641. 10.1002/advs.202304641 PMC10787108 37933988

[239] M. Wu , H. Liu , Y. Zhu , F. Chen , Z. Chen , L. Guo , P. Wu , G. Li , C. Zhang , R. Wei , L. Cai , Small 2023, 19, 2300111. 10.1002/smll.202300111 37191242

[240] L. Tong , Q. Liao , Y. Zhao , H. Huang , A. Gao , W. Zhang , X. Gao , W. Wei , M. Guan , P. K. Chu , H. Wang , Biomaterials 2019, 193, 1. 30550998 10.1016/j.biomaterials.2018.12.008

[241] L. Kuang , J. Huang , Y. Liu , X. Li , Y. Yuan , C. Liu , Adv. Funct. Mater. 2021, 31, 2105383.

[242] Z. Yuan , J. Wu , Z. Fu , S. Meng , L. Dai , K. Cai , Adv. Funct. Mater. 2022, 32, 2200374.

[243] Y. Pan , Y. Xiao , Y. Hao , K. Shi , M. Pan , Z. Y. Qian , Chin. Chem. Lett. 2022, 33, 2486.

[244] X. Ji , H. Shao , X. Li , M. W. Ullah , G. Luo , Z. Xu , L. Ma , X. He , Z. Lei , Q. Li , X. Jiang , G. Yang , Y. Zhang , Biomaterials 2022, 285, 121530. 35504181 10.1016/j.biomaterials.2022.121530

[245] X. Ji , X. Yuan , L. Ma , B. Bi , H. Zhu , Z. Lei , W. Liu , H. Pu , J. Jiang , X. Jiang , Y. Zhang , J. Xiao , Theranostics 2020, 10, 725. 31903147 10.7150/thno.39167 PMC6929983

[246] Z. Li , H. Wang , K. Zhang , B. Yang , X. Xie , Z. Yang , L. Kong , P. Shi , Y. Zhang , Y. P. Ho , Z. Y. Zhang , G. Li , L. Bian , Bioact Mater 2022, 13, 9. 35224288 10.1016/j.bioactmat.2021.11.004 PMC8844702

[247] G. Cai , L. Ren , J. Yu , S. Jiang , G. Liu , S. Wu , B. Cheng , W. Li , J. Xia , Adv. Sci. 2024, 11, 2403786. 10.1002/advs.202403786 PMC11425865 38978324

[248] F. Zhang , Q. Hu , Y. Wei , W. Meng , R. Wang , J. Liu , Y. Nie , R. Luo , Y. Wang , B. Shen , Chem. Eng. J. 2022, 435, 134802.

[249] R. J. Crum , K. Hall , C. P. Molina , G. S. Hussey , E. Graham , H. Li , S. F. Badylak , NPJ Regen. Med. 2022, 7, 13. 35110573 10.1038/s41536-022-00208-9 PMC8810774

[250] Y. Xie , L. Zhang , Q. Xiong , Y. Gao , W. Ge , P. Tang , Bone Res. 2019, 7, 25. 31646015 10.1038/s41413-019-0066-7 PMC6804735

[251] Y. Yang , L. Guo , Z. Wang , P. Liu , X. Liu , J. Ding , W. Zhou , Biomaterials 2021, 264, 120390. 32980634 10.1016/j.biomaterials.2020.120390

[252] K. Zhou , C. Yang , K. Shi , Y. Liu , D. Hu , X. He , Y. Yang , B. Chu , J. Peng , Z. Zhou , Z. Qian , Biomaterials 2023, 295, 122036. 36804660 10.1016/j.biomaterials.2023.122036

[253] M. Jeong , Y. Jung , J. Yoon , J. Kang , S. H. Lee , W. Back , H. Kim , M. J. Sailor , D. Kim , J. H. Park , ACS Nano 2022, 16, 16118. 36214219 10.1021/acsnano.2c04491

[254] H. Liang , B. Peng , C. Dong , L. Liu , J. Mao , S. Wei , X. Wang , H. Xu , J. Shen , H. Q. Mao , X. Gao , K. W. Leong , Y. Chen , Nat. Commun. 2018, 9, 4291. 30327464 10.1038/s41467-018-06603-5 PMC6191420

[255] X. Chen , L. Zhang , H. Zeng , W. Meng , G. Liu , W. Zhang , P. Zhao , Q. Zhang , M. Chen , J. Chen , Small 2023, 19, 2304610. 10.1002/smll.202304610 37632302

[256] R. Yang , L. Yan , T. Xu , K. Zhang , X. Lu , C. Xie , W. Fu , Biomaterials 2024, 311, 122706. 39032219 10.1016/j.biomaterials.2024.122706

[257] S. Koo , H. S. Sohn , T. H. Kim , S. Yang , S. Y. Jang , S. Ye , B. Choi , S. H. Kim , K. S. Park , H. M. Shin , O. K. Park , C. Kim , M. Kang , M. Soh , J. Yoo , D. Kim , N. Lee , B. S. Kim , Y. Jung , T. Hyeon , Nat. Nanotechnol. 2023, 18, 1502. 37884660 10.1038/s41565-023-01523-y

[258] I. R. Reid , E. O. Billington , Lancet 2022, 399, 1080. 35279261 10.1016/S0140-6736(21)02646-5

[259] R. X. Liu , R. H. Gu , Z. P. Li , Z. Q. Hao , Q. X. Hu , Z. Y. Li , X. G. Wang , W. Tang , X. H. Wang , Y. K. Zeng , Z. W. Li , Q. Dong , X. F. Zhu , D. Chen , K. W. Zhao , R. H. Zhang , Z. G. Zha , H. T. Zhang , Bone Res. 2023, 11, 56. 37884520 10.1038/s41413-023-00296-3 PMC10603047

[260] B. Sun , H. F. Wang , B. Xiao , H. C. Yan , H. Q. Wu , R. C. Zhang , Y. Zhang , W. Yuan , X. Wang , C. G. Shi , Chem. Eng. J. 2023, 476, 146743.

[261] G. Livshits , A. Kalinkovich , Life Sci. 2022, 306, 120847. 35908619 10.1016/j.lfs.2022.120847

[262] M. Chen , M. Li , Y. Wei , C. Xue , M. Chen , Y. Fei , L. Tan , Z. Luo , K. Cai , Y. Hu , Biomaterials 2022, 291, 121878. 36335716 10.1016/j.biomaterials.2022.121878

[263] C. Zhang , C. Song , Front. Pharmacol. 2020, 11, 607017. 33584284 10.3389/fphar.2020.607017 PMC7874063

[264] J. Li , L. Li , T. Wu , K. Shi , Z. Bei , M. Wang , B. Chu , K. Xu , M. Pan , Y. Li , X. Hu , L. Zhang , Y. Qu , Z. Qian , Small Methods 2024, 8, 2300843. 10.1002/smtd.202300843 37800985

[265] L. Bosca , M. Zeini , P. G. Traves , S. Hortelano , Toxicology 2005, 208, 249. 15691589 10.1016/j.tox.2004.11.035

[266] J. K. Lee , D. S. Kim , S. Y. Park , S. W. Baek , J. W. Jung , T. H. Kim , D. K. Han , Adv. Sci. 2023, 10, 2205336. 10.1002/advs.202205336 PMC9951336 36581472

[267] L. Che , Y. Wang , D. Sha , G. Li , Z. Wei , C. Liu , Y. Yuan , D. Song , Bioact. Mater. 2023, 19, 75. 35441117 10.1016/j.bioactmat.2022.03.023 PMC8990063

[268] J. Qian , H. Qin , E. Su , J. Hou , H. Zeng , T. Wang , D. Wang , G. Wan , Y. Chen , Bioact. Mater. 2025, 44, 46.

[269] R. Zhao , H. Qian , X. Zhu , X. Zhang , Z. Chen , X. Yang , Adv. Funct. Mater. 2024, 34, 2401566.

[270] Y. Zheng , Z. Zhang , Z. Fu , A. Fan , N. Song , Q. Wang , S. Fan , J. Xu , J. Xiang , X. Liu , ACS Nano 2024, 18, 26153. 10.1021/acsnano.4c07332 39269339

[271] Y. Huang , T. Chen , C. Ren , B. Bao , R. Huang , Y. Sun , C. Yu , Y. Yang , W. T. Wong , Q. Zeng , L. Jiang , T. Liu , Q. Lin , L. Zhu , Y. Liao , Adv. Mater. 2025, 37, 2501051. 10.1002/adma.202501051 39972948

[272] X. Yang , Y. Fan , J. Liang , R. Cao , B. Zhang , J. Li , Z. Li , S. He , N. Liu , J. Du , Y. Hu , ACS Nano 2024, 18, 22431. 39103298 10.1021/acsnano.4c07265

[273] X. T. He , X. Li , M. Zhang , B. M. Tian , L. J. Sun , C. S. Bi , D. K. Deng , H. Zhou , H. L. Qu , C. Wu , F. M. Chen , Biomaterials 2022, 283, 121439. 35247634 10.1016/j.biomaterials.2022.121439

[274] X. Zhang , N. Han , G. Li , H. Yang , Y. Cao , Z. Fan , F. Zhang , Int. J. Oral Sci. 2018, 10, 19. 29895944 10.1038/s41368-018-0020-3 PMC5997630

[275] Y. Li , L. Yang , Y. Hou , Z. Zhang , M. Chen , M. Wang , J. Liu , J. Wang , Z. Zhao , C. Xie , X. Lu , Bioact. Mater. 2022, 18, 213. 35387166 10.1016/j.bioactmat.2022.03.021 PMC8961429

[276] C. Ni , J. Zhou , N. Kong , T. Bian , Y. Zhang , X. Huang , Y. Xiao , W. Yang , F. Yan , Biomaterials 2019, 206, 115. 30933774 10.1016/j.biomaterials.2019.03.039

[277] X. T. He , X. Li , Y. Xia , Y. Yin , R. X. Wu , H. H. Sun , F. M. Chen , Acta Biomater. 2019, 88, 162. 30735811 10.1016/j.actbio.2019.02.004

[278] Z. Q. Liu , L. L. Shang , S. H. Ge , J. Dent. Sci. 2021, 16, 937. 34141108 10.1016/j.jds.2020.10.008 PMC8189879

[279] T. Kagioka , S. Itoh , M. T. Hue , M. Abe , M. Hayashi , Sci. Rep. 2023, 13, 7886. 37193735 10.1038/s41598-023-34700-z PMC10188564

[280] A. Sculean , I. L. Chapple , W. V. Giannobile , Periodontol 2000 2015, 68, 7. 25867976 10.1111/prd.12091 PMC4441284

[281] C. Wang , Q. Zhao , C. Chen , J. Li , J. Zhang , S. Qu , H. Tang , H. Zeng , Y. Zhang , Int. J. Oral Sci. 2023, 15, 19. 37198150 10.1038/s41368-023-00225-4 PMC10192316

[282] L. Li , H. Jiang , R. Chen , J. Zhou , Y. Xiao , Y. Zhang , F. Yan , Int. J. Oral Sci. 2020, 12, 13. 32350241 10.1038/s41368-020-0078-6 PMC7190824

[283] B. Zhu , J. Wu , T. Li , S. Liu , J. Guo , Y. Yu , X. Qiu , Y. Zhao , H. Peng , J. Zhang , L. Miao , H. Wei , Adv. Healthcare Mater. 2024, 13, 2302485. 10.1002/adhm.202302485 37902093

[284] B. Tian , X. Li , J. Zhang , M. Zhang , D. Gan , D. Deng , L. Sun , X. He , C. Wu , F. Chen , Int. J. Oral Sci. 2022, 14, 45. 36064833 10.1038/s41368-022-00195-z PMC9445063

[285] Y. He , M. Tian , X. Li , J. Hou , S. Chen , G. Yang , X. Liu , S. Zhou , Adv. Healthcare Mater. 2022, 11, 2102236. 10.1002/adhm.202102236 34779582

[286] J. Luo , H. Chen , G. Wang , J. Lyu , Y. Liu , S. Lin , M. Zhou , X. Jiang , Adv. Healthcare Mater. 2023, 12, 2301366. 10.1002/adhm.202301366 37515813

[287] Y. Xu , Y. Luo , Z. Weng , H. Xu , W. Zhang , Q. Li , H. Liu , L. Liu , Y. Wang , X. Liu , L. Liao , X. Wang , ACS Nano 2023, 17, 18732. 37768714 10.1021/acsnano.3c01940

[288] L. Zheng , B. Hu , D. Zhao , W. Liu , Q. Liu , Y. Huang , S. Ruan , Chin. Chem. Lett. 2024, 35, 108647.

[289] Y. Cui , S. Hong , Y. Xia , X. Li , X. He , X. Hu , Y. Li , X. Wang , K. Lin , L. Mao , Adv. Sci. 2023, 10, 2302029. 10.1002/advs.202302029 PMC10520618 37452425

[290] Z. Zhao , C. Wu , Y. Huangfu , Y. Zhang , J. Zhang , P. Huang , A. Dong , Y. Wang , J. Deng , W. Wang , Z. Feng , ACS Nano 2024, 18, 29507. 39401162 10.1021/acsnano.4c05677

[291] N. Yan , J. Xu , G. Liu , C. Ma , L. Bao , Y. Cong , Z. Wang , Y. Zhao , W. Xu , C. Chen , ACS Nano 2022, 16, 18253. 36288552 10.1021/acsnano.2c05923

[292] X. Chen , H. Huang , C. Guo , X. Zhu , J. Chen , J. Liang , R. Yang , D. Shao , F. Chen , B. Shi , C. Yang , K. W. Leong , L. Zhao , Adv. Funct. Mater. 2024, 35, 2409121.

[293] A. G. Gristina , M. Oga , L. X. Webb , C. D. Hobgood , Science 1985, 228, 990. 4001933 10.1126/science.4001933

[294] Y. Li , J. Li , Y. Zhong , Q. Zhang , Y. Wu , J. Huang , K. Pang , Y. Zhou , T. Xiao , Z. Wu , W. Sun , Biomaterials 2025, 313, 122762. 39178559 10.1016/j.biomaterials.2024.122762

[295] J. Guo , X. Shu , S. Yu , C. Guo , G. Shen , L. Chen , J. Zhou , J. Xiao , H. Guo , Y. Chen , Z. Zeng , P. Wang , J. Control. Release 2024, 376, 337. 39413850 10.1016/j.jconrel.2024.10.021

[296] G. Lu , G. Zhao , S. Wang , H. Li , Q. Yu , Q. Sun , B. Wang , L. Wei , Z. Fu , Z. Zhao , L. Yang , L. Deng , X. Zheng , M. Cai , M. Lu , Adv. Sci. 2024, 11, 2306964. 10.1002/advs.202306964 PMC10966557 38234236

[297] Z. Han , S. Lu , J. Cao , S. Sun , N. Yang , S. Cheng , X. Huang , J. Wu , J. Li , L. Cheng , Mater. Today 2025, 82, 32.

[298] H. Lv , M. Yang , Y. Yang , Z. Tang , Y. Guo , J. Zhou , Y. Gui , R. Huang , J. Cai , B. Yu , J. Yang , Y. Bao , Z. Zhang , D. Zhang , T. Hou , ACS Nano 2025, 19, 5253. 39886847 10.1021/acsnano.4c11956

[299] H. Lin , C. Yang , Y. Luo , M. Ge , H. Shen , X. Zhang , J. Shi , ACS Nano 2022, 16, 5943. 35316599 10.1021/acsnano.1c11132

[300] A. Vishwakarma , N. S. Bhise , M. B. Evangelista , J. Rouwkema , M. R. Dokmeci , A. M. Ghaemmaghami , N. E. Vrana , A. Khademhosseini , Trends Biotechnol. 2016, 34, 470. 27138899 10.1016/j.tibtech.2016.03.009

[301] Y. Zheng , X. Chen , Q. Zhang , L. Yang , Q. Chen , Z. Chen , Y. Wang , D. Wu , Adv. Healthcare Mater. 2024, 13, 2401816.

[302] J. Deng , X. Wang , W. Zhang , L. Sun , X. Han , X. Tong , L. Yu , J. Ding , L. Yu , Y. Liu , Adv. Funct. Mater. 2023, 33, 2211664.

